# Supplementary figures and images for: Effector target-guided engineering of an integrated domain expands the disease resistance profile of a rice NLR immune receptor
Source: eLife. 2023 May 18;12:e81123. doi: 10.7554/eLife.81123 (PMC10195085; doi:10.7554/eLife.81123)

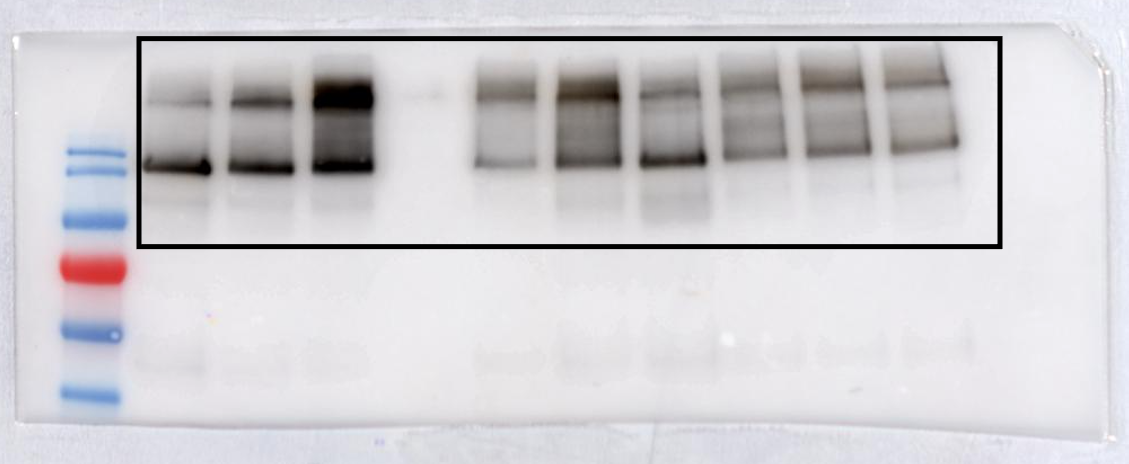

Supplement: Figure 1—source data 1. [file elife-81123-fig1-data1.tiff]

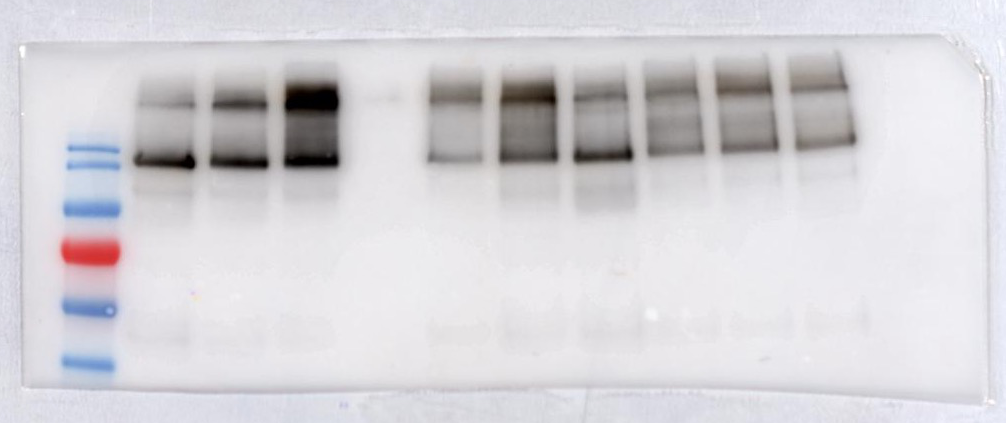

Supplement: Figure 1—source data 2. [file elife-81123-fig1-data2.tiff]

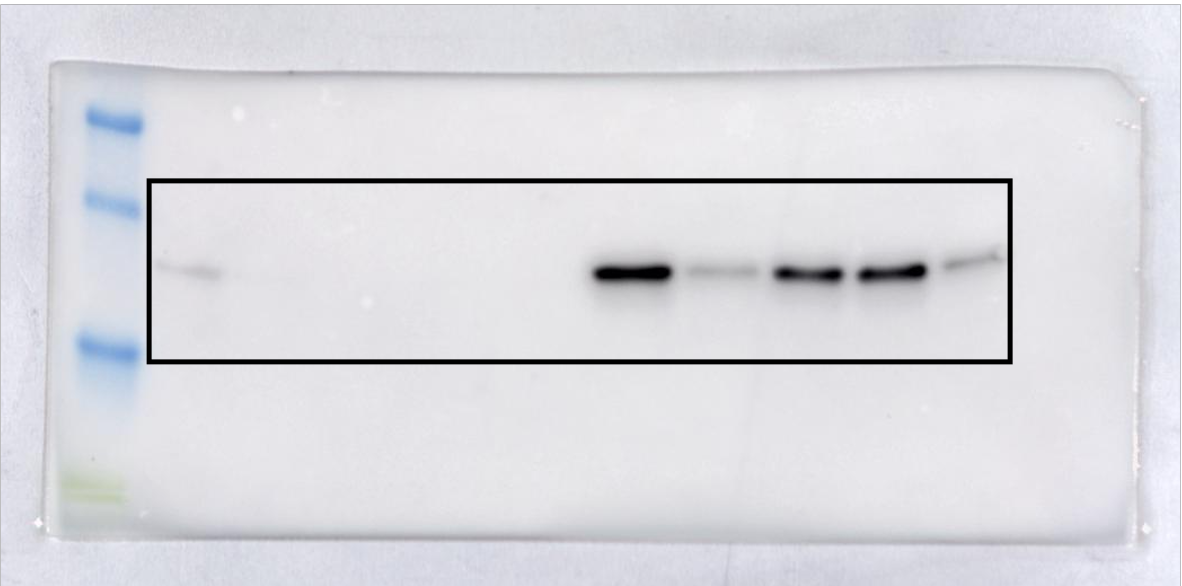

Supplement: Figure 1—source data 3. [file elife-81123-fig1-data3.tiff]

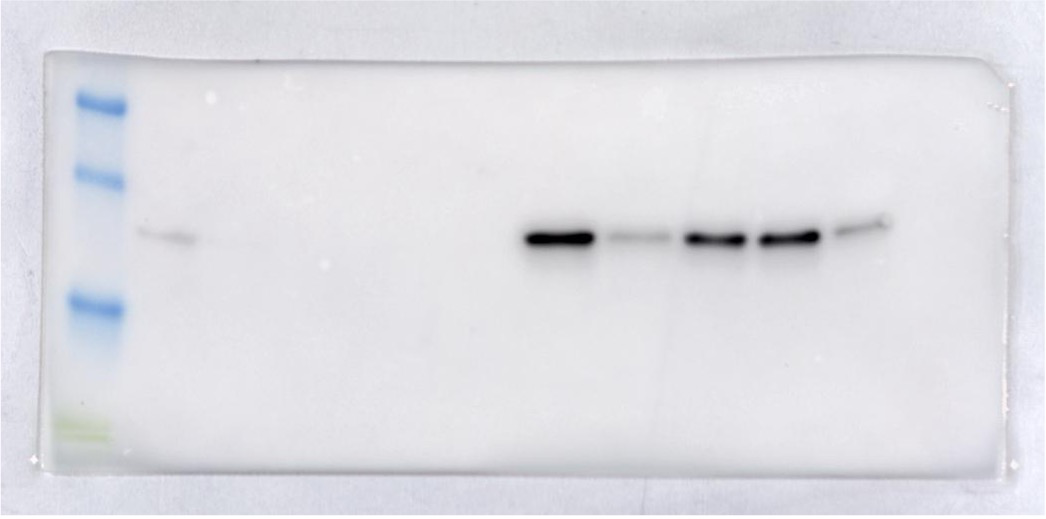

Supplement: Figure 1—source data 4. [file elife-81123-fig1-data4.tiff]

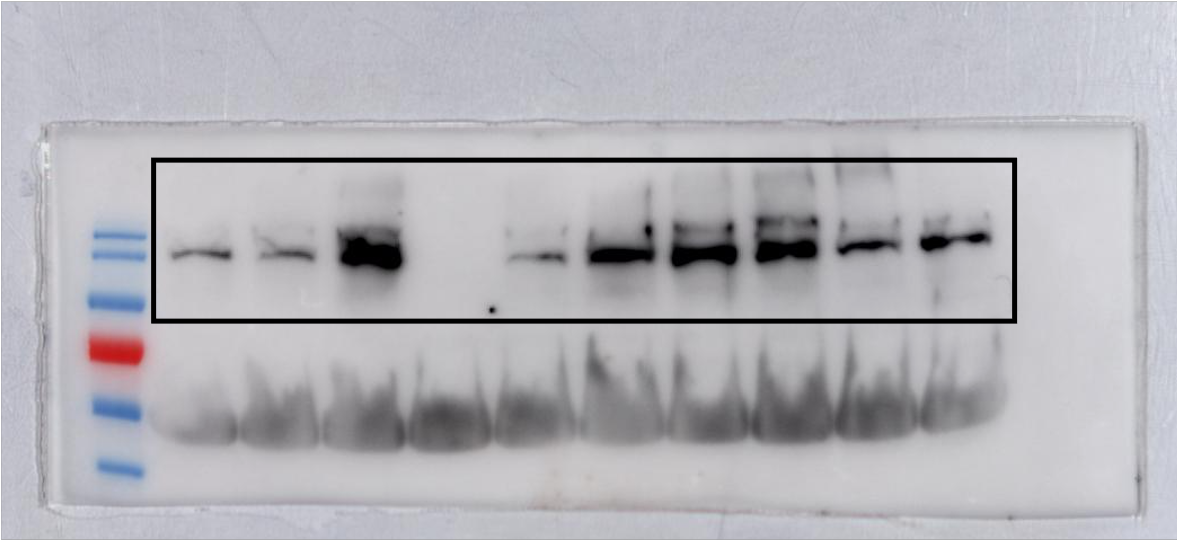

Supplement: Figure 1—source data 5. [file elife-81123-fig1-data5.tiff]

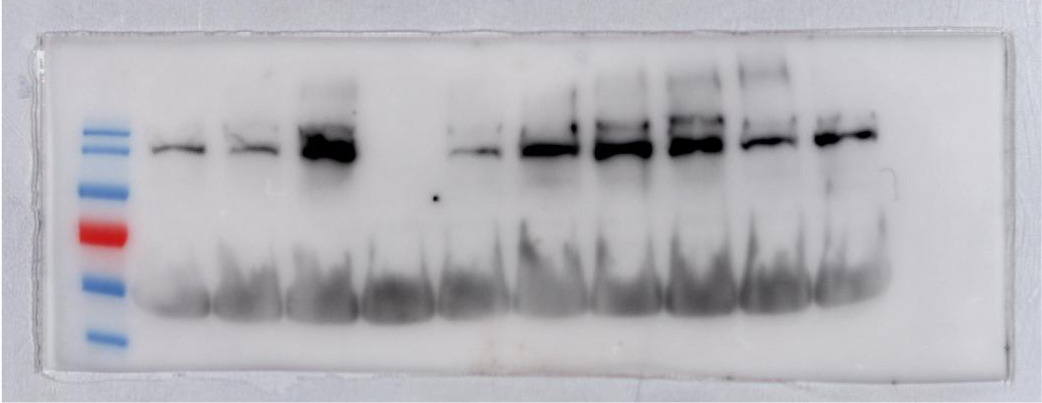

Supplement: Figure 1—source data 6. [file elife-81123-fig1-data6.tiff]

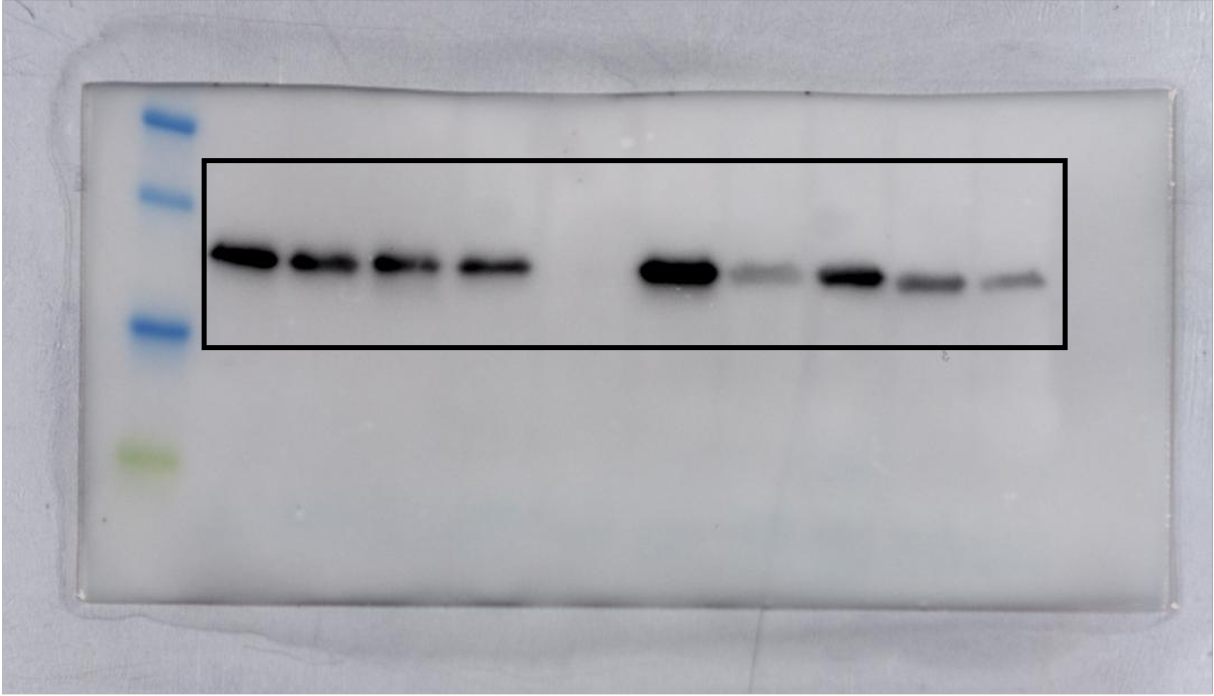

Supplement: Figure 1—source data 7. [file elife-81123-fig1-data7.tiff]

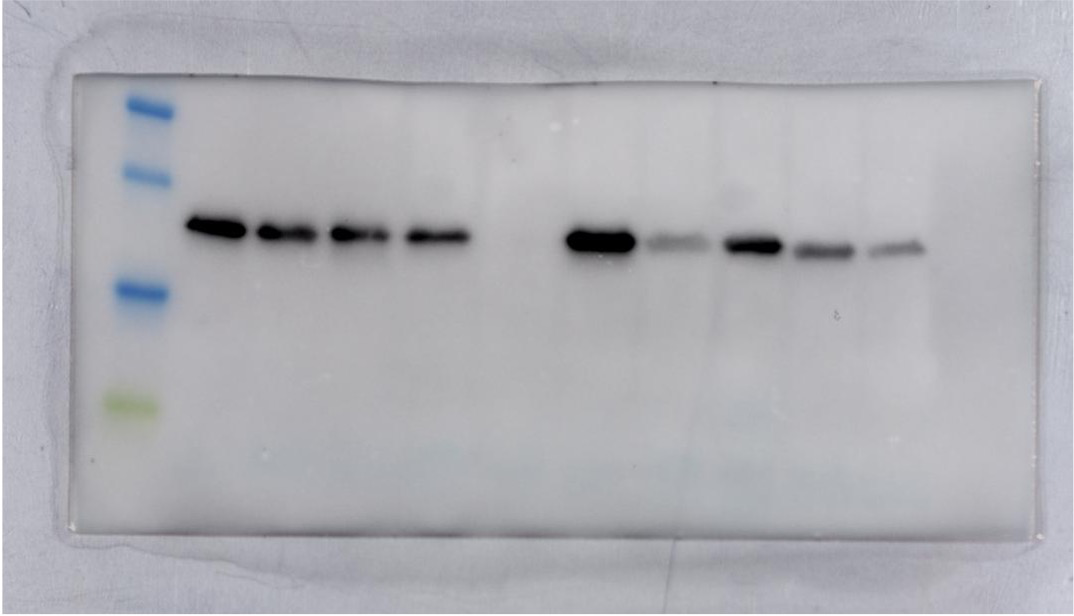

Supplement: Figure 1—source data 8. [file elife-81123-fig1-data8.tiff]

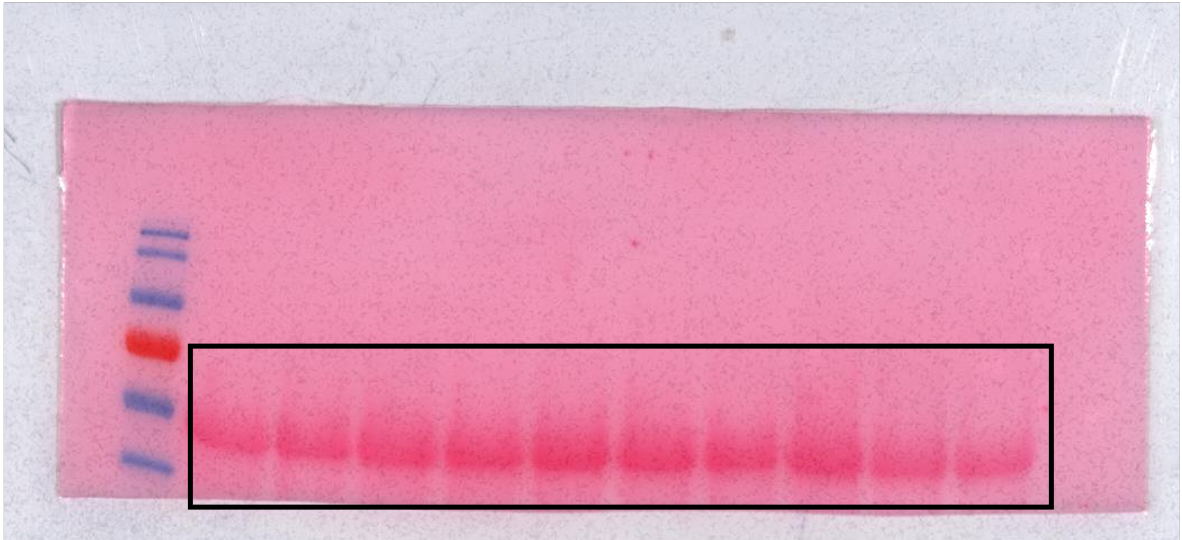

Supplement: Figure 1—source data 9. [file elife-81123-fig1-data9.tiff]

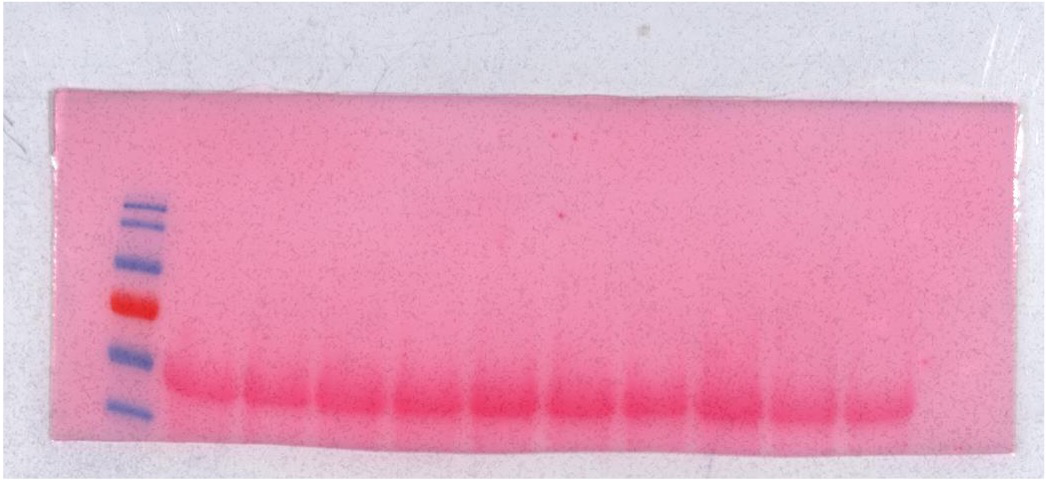

Supplement: Figure 1—source data 10. [file elife-81123-fig1-data10.tiff]

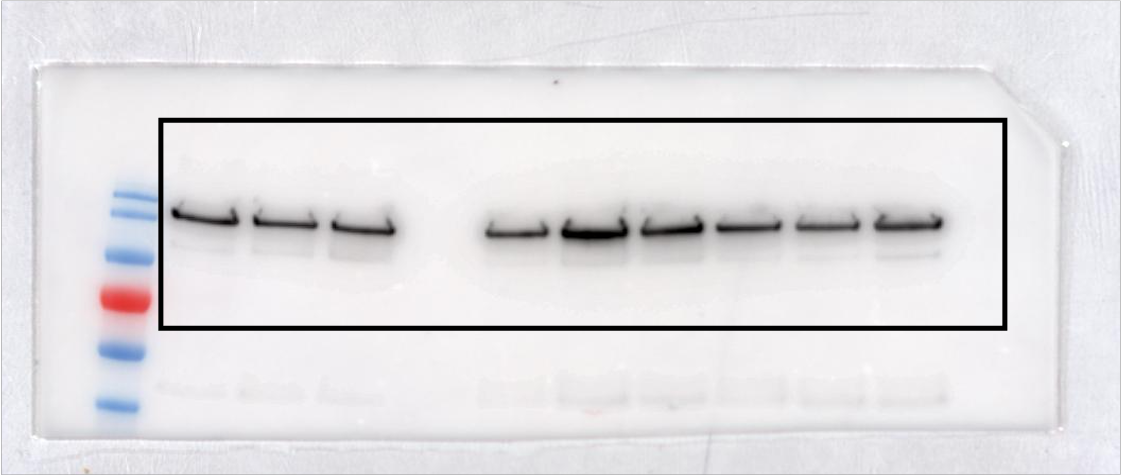

Supplement: Figure 1—figure supplement 3—source data 1. [file elife-81123-fig1-figsupp3-data1.tiff]

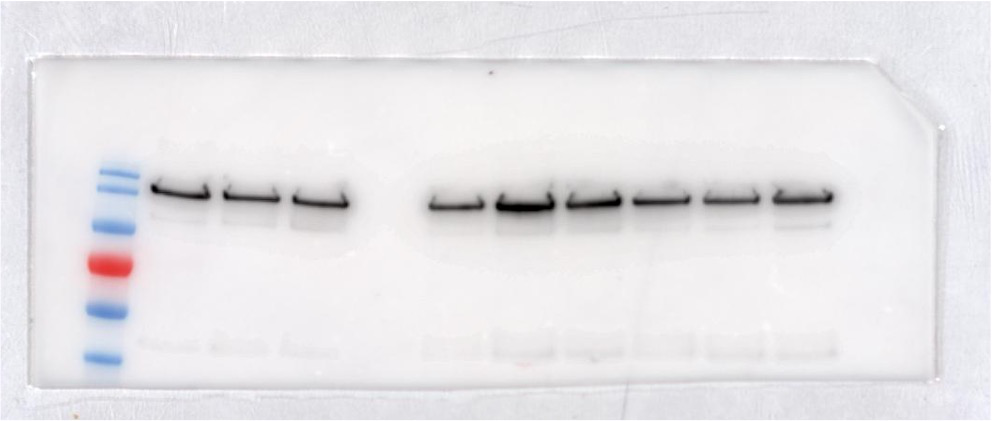

Supplement: Figure 1—figure supplement 3—source data 2. [file elife-81123-fig1-figsupp3-data2.tiff]

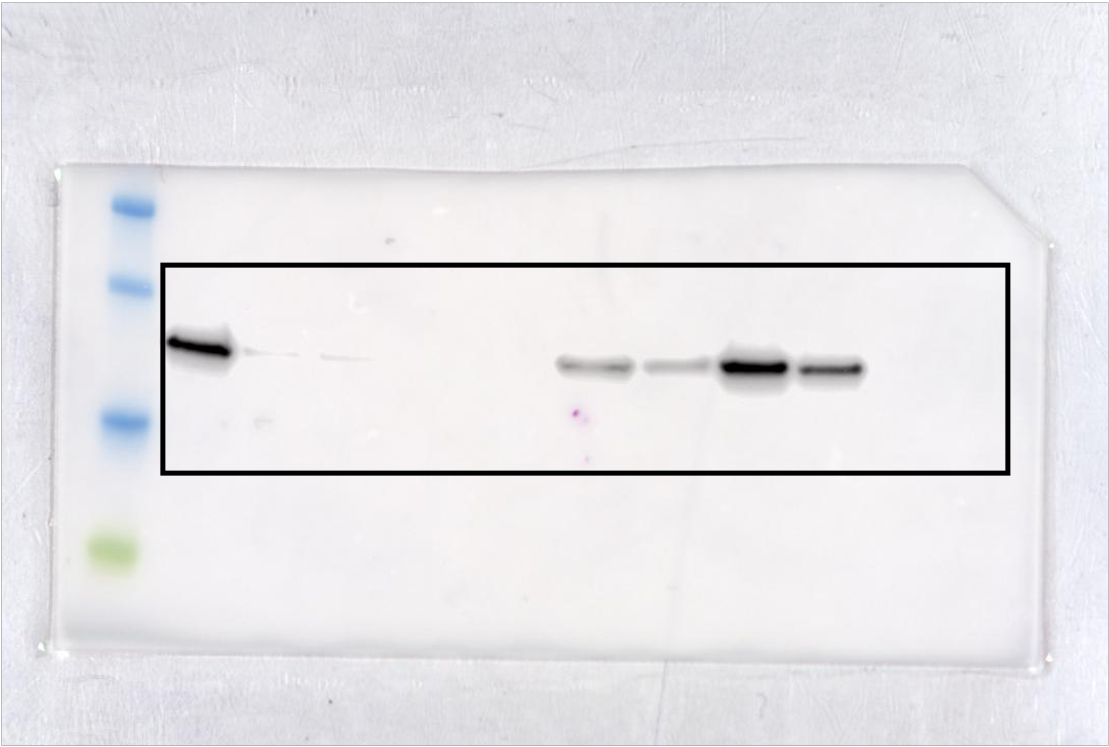

Supplement: Figure 1—figure supplement 3—source data 3. [file elife-81123-fig1-figsupp3-data3.tiff]

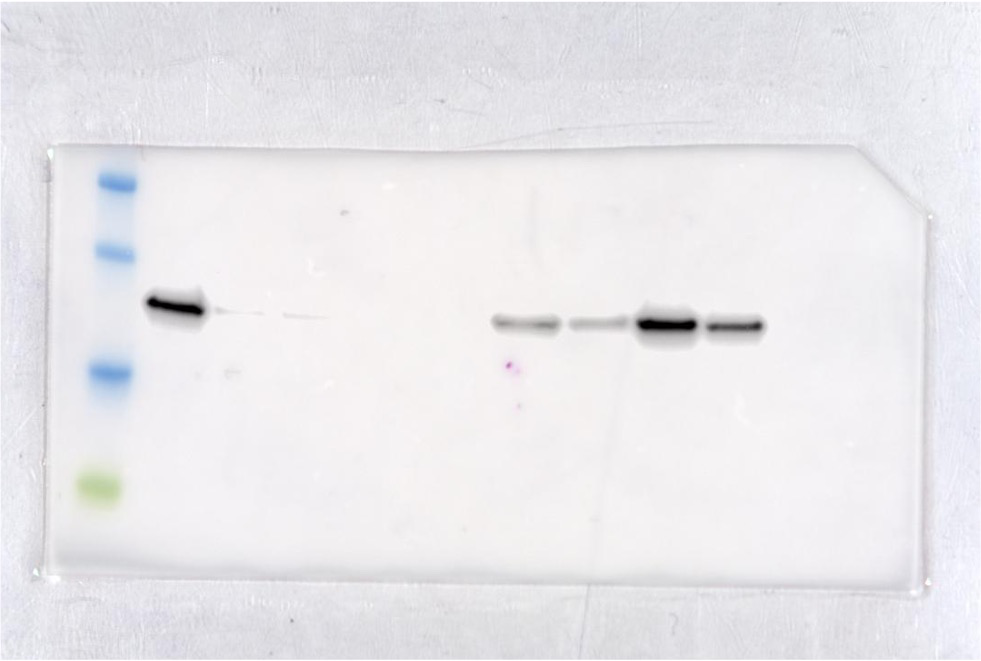

Supplement: Figure 1—figure supplement 3—source data 4. [file elife-81123-fig1-figsupp3-data4.tiff]

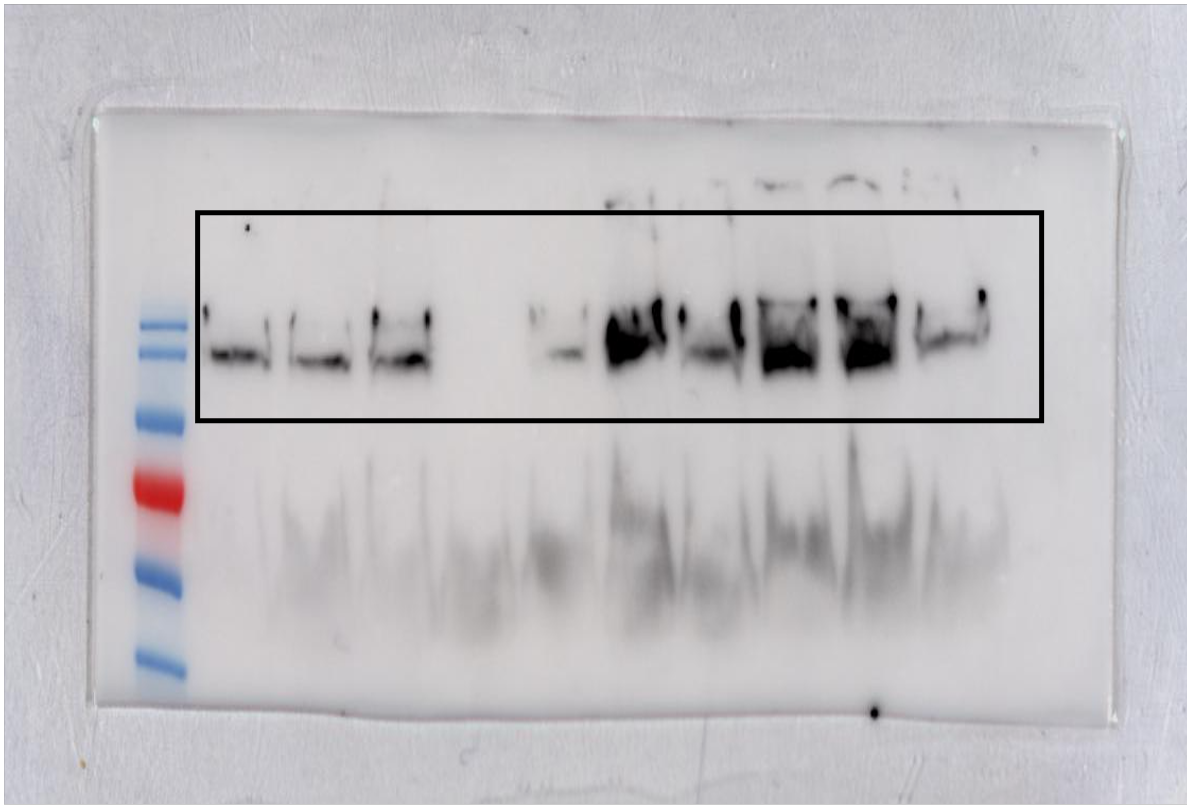

Supplement: Figure 1—figure supplement 3—source data 5. [file elife-81123-fig1-figsupp3-data5.tiff]

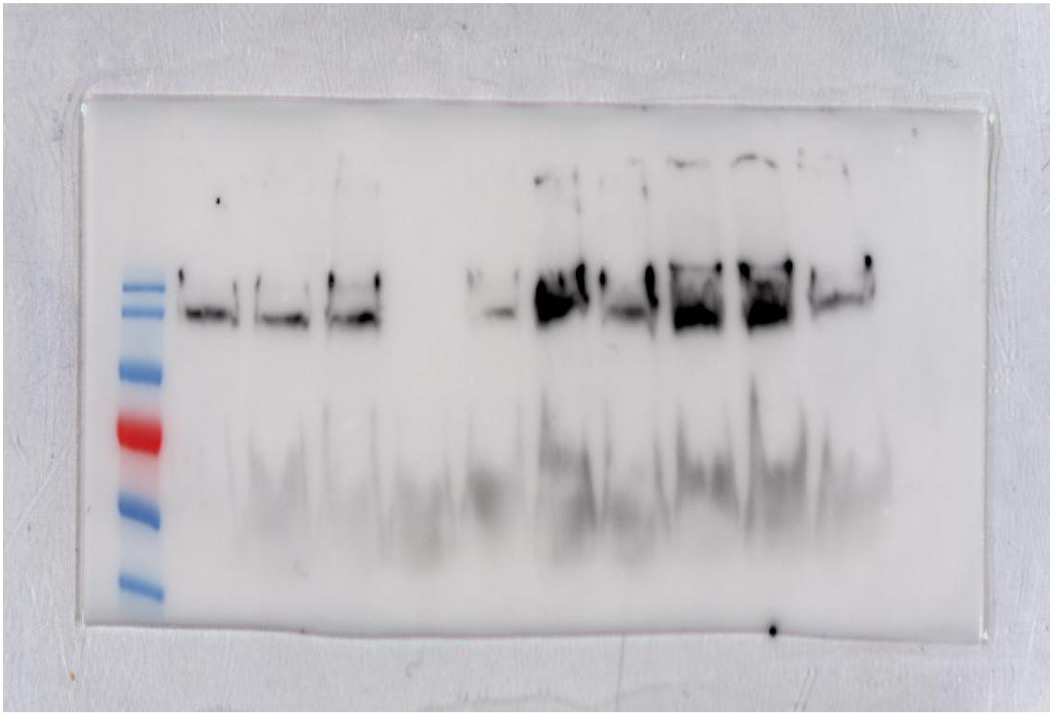

Supplement: Figure 1—figure supplement 3—source data 6. [file elife-81123-fig1-figsupp3-data6.tiff]

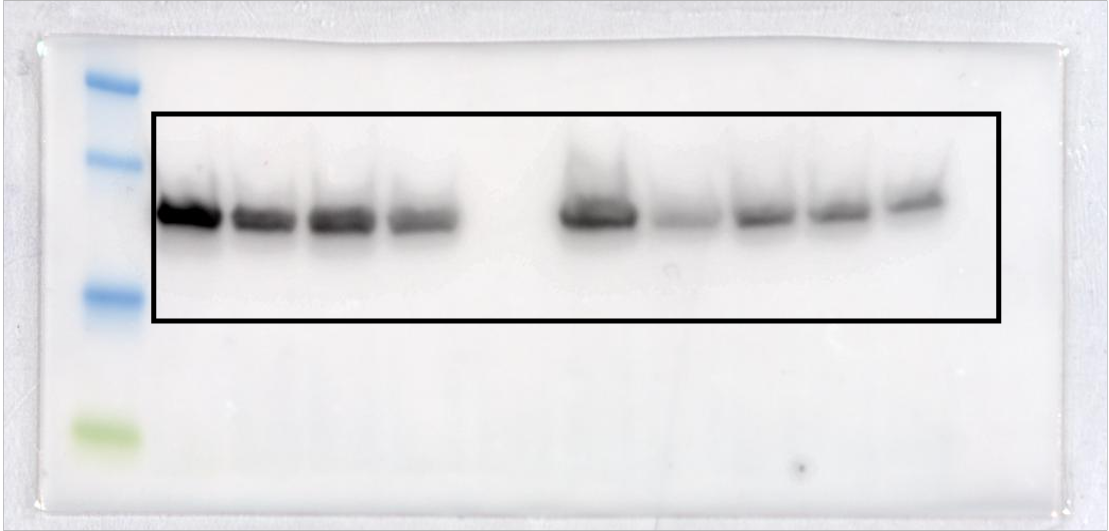

Supplement: Figure 1—figure supplement 3—source data 7. [file elife-81123-fig1-figsupp3-data7.tiff]

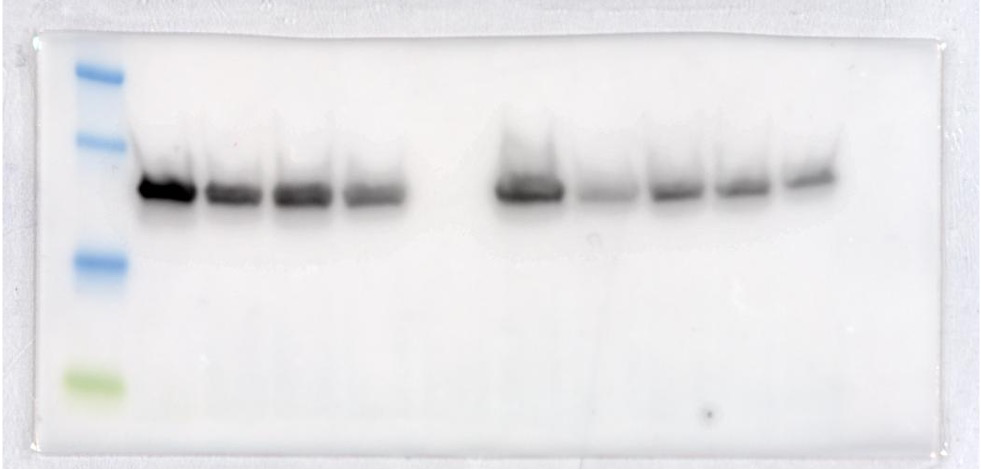

Supplement: Figure 1—figure supplement 3—source data 8. [file elife-81123-fig1-figsupp3-data8.tiff]

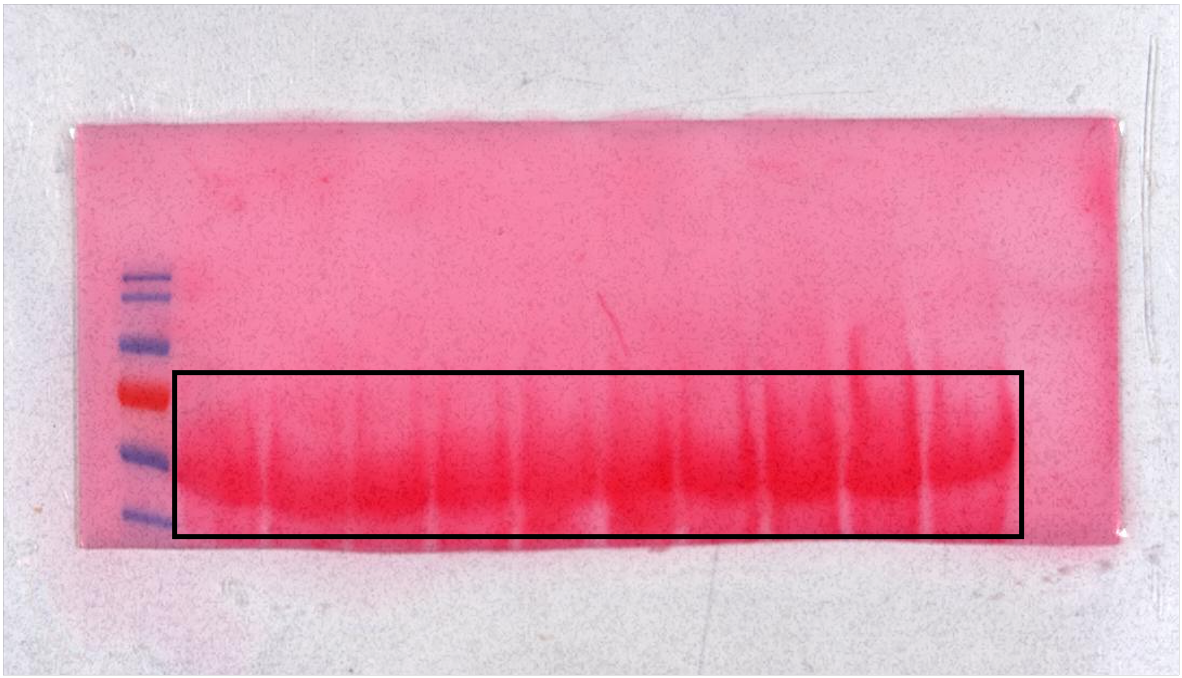

Supplement: Figure 1—figure supplement 3—source data 9. [file elife-81123-fig1-figsupp3-data9.tiff]

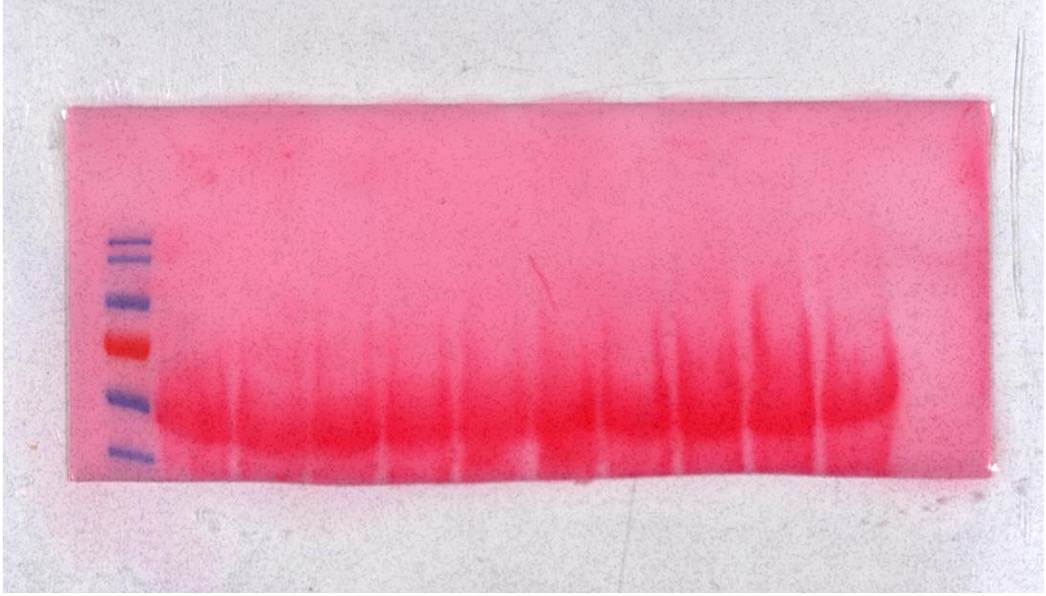

Supplement: Figure 1—figure supplement 3—source data 10. [file elife-81123-fig1-figsupp3-data10.tiff]

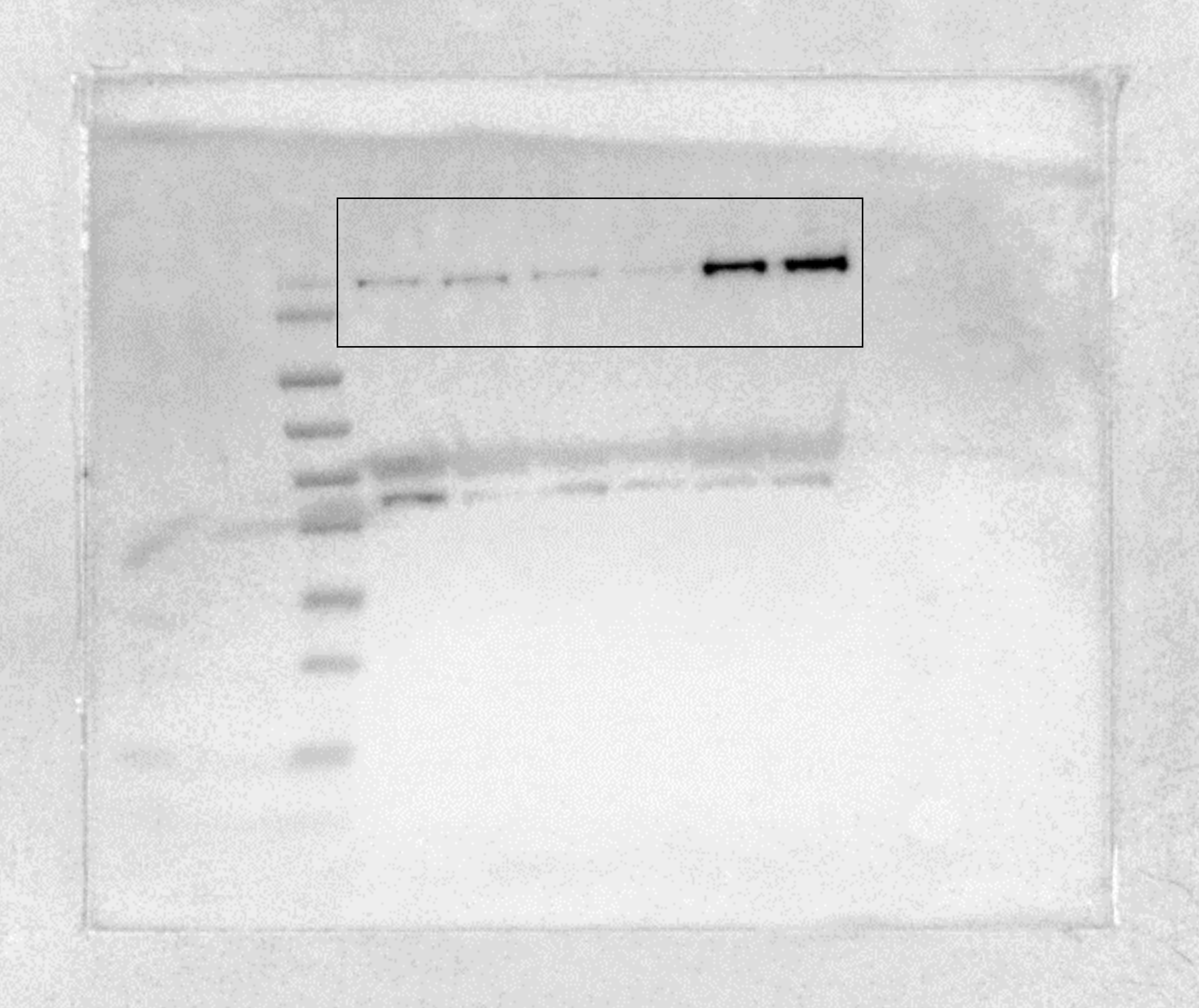

Supplement: Figure 1—figure supplement 7—source data 1. [file elife-81123-fig1-figsupp7-data1.tiff]

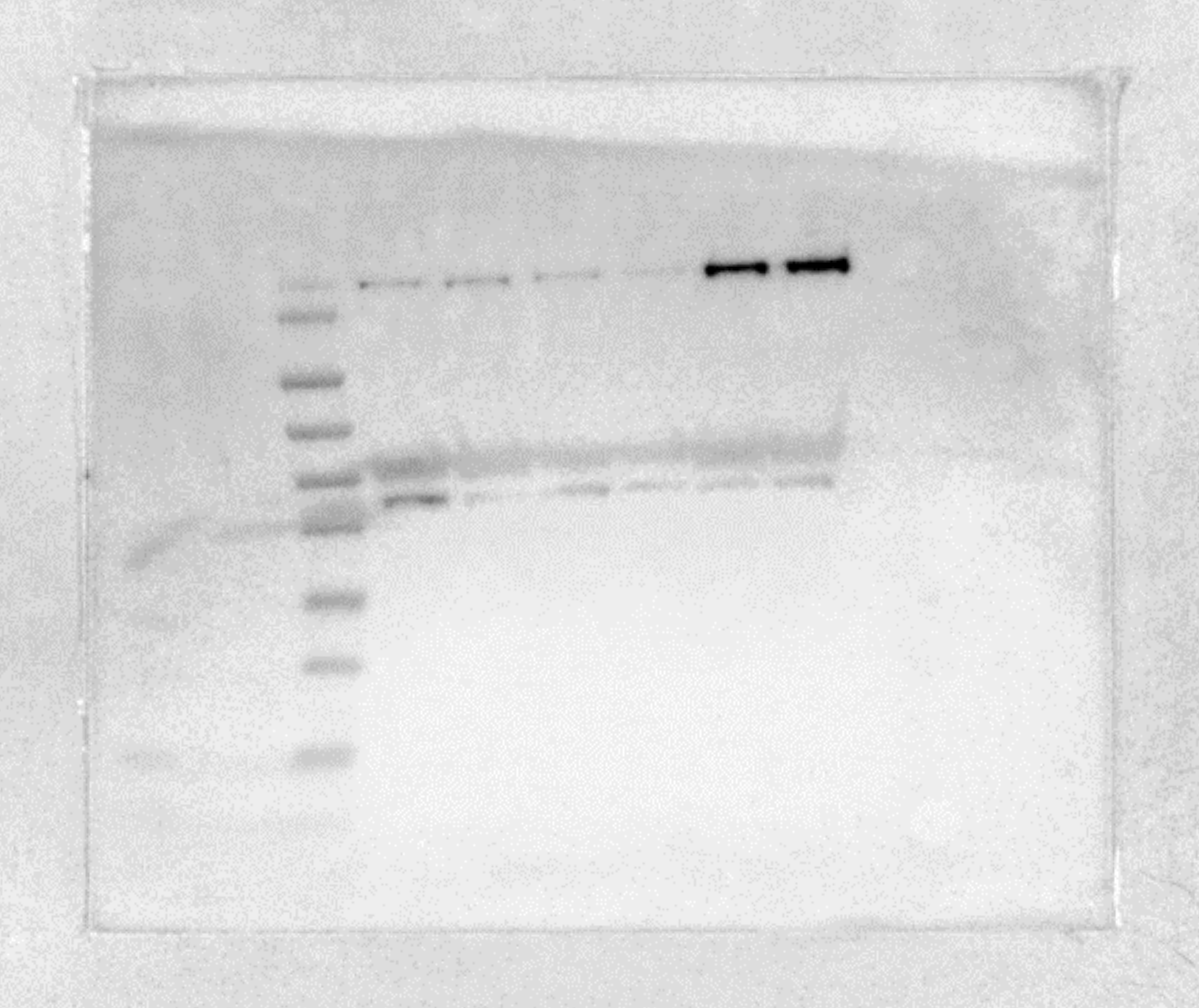

Supplement: Figure 1—figure supplement 7—source data 2. [file elife-81123-fig1-figsupp7-data2.tiff]

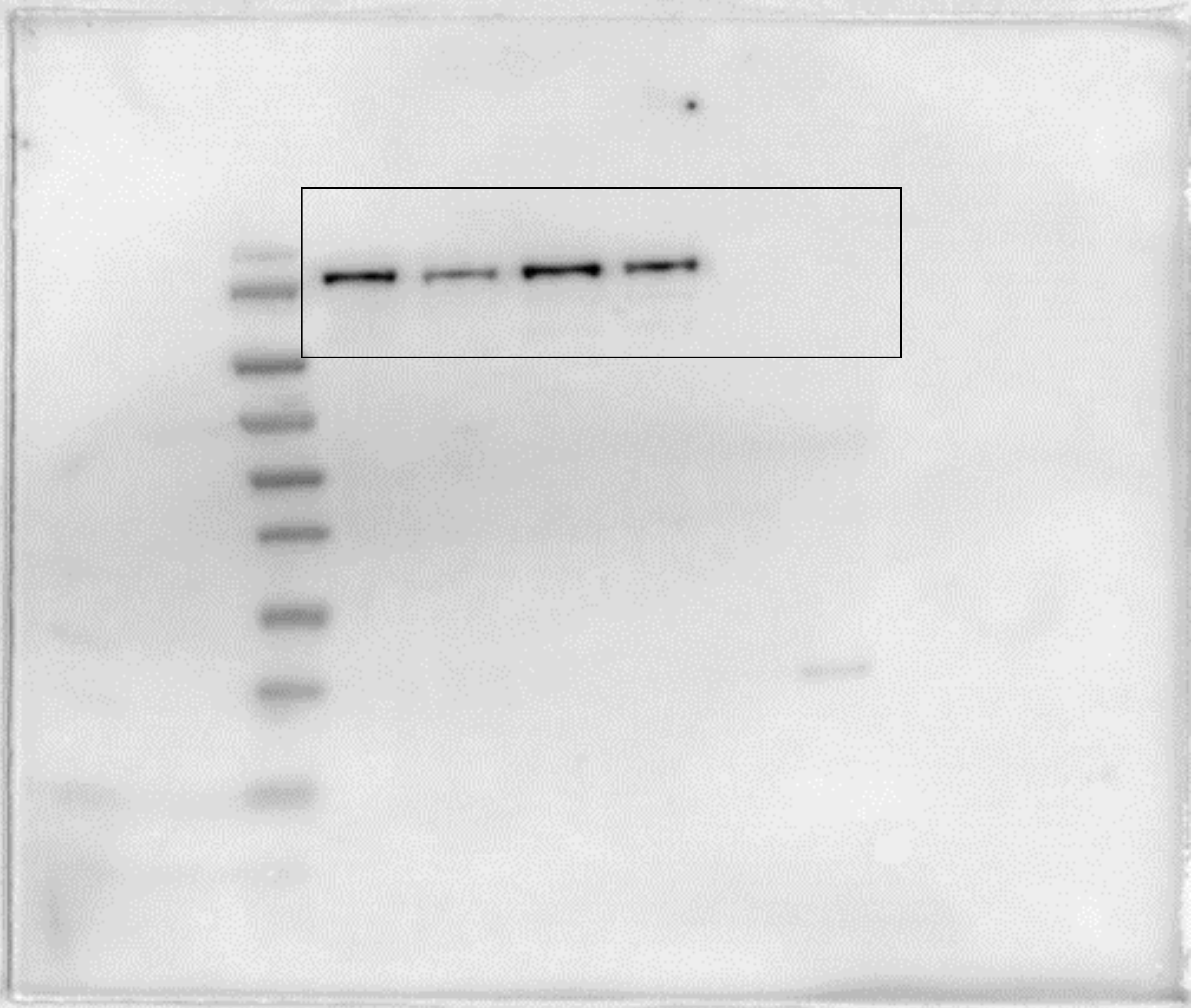

Supplement: Figure 1—figure supplement 7—source data 3. [file elife-81123-fig1-figsupp7-data3.tiff]

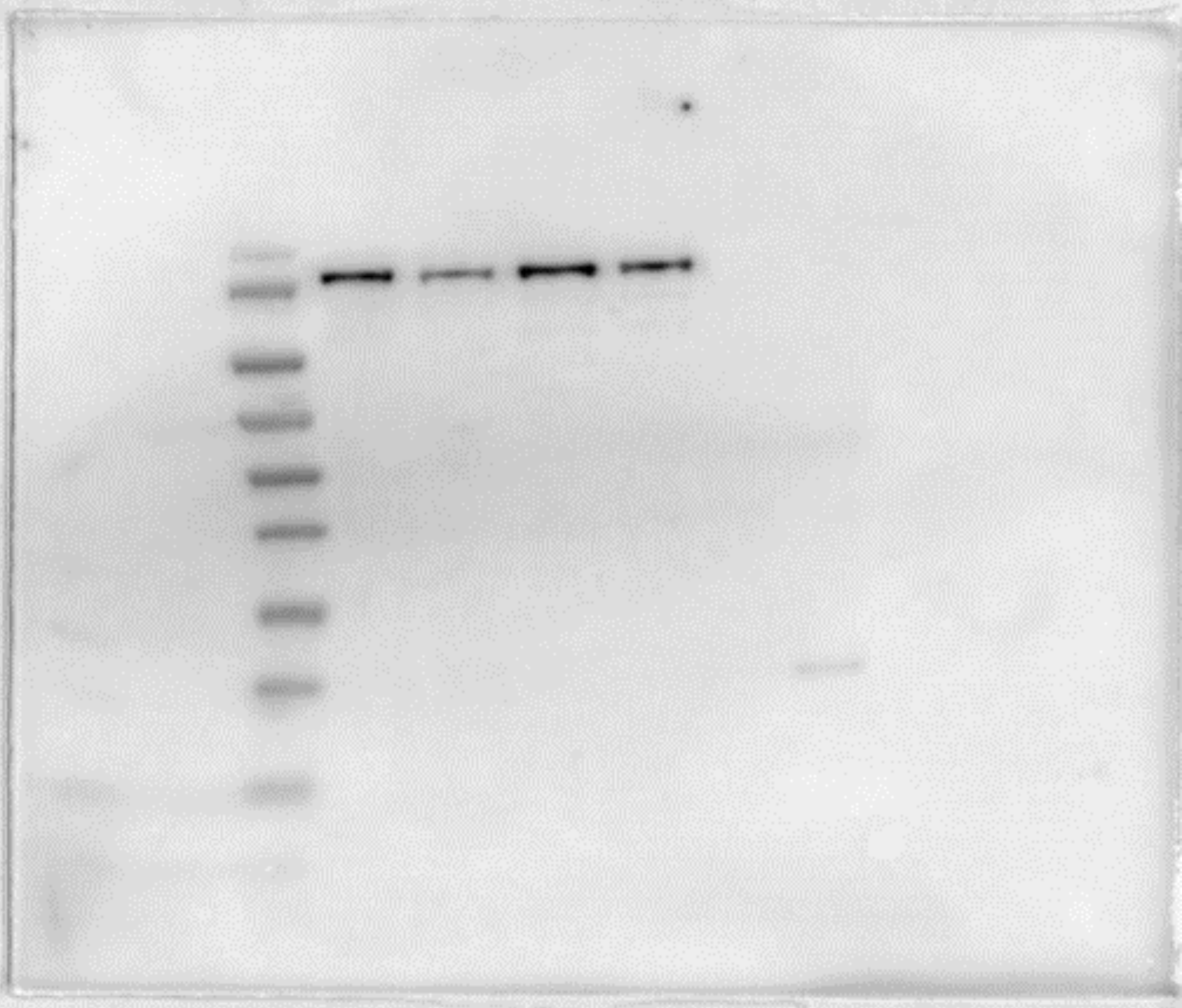

Supplement: Figure 1—figure supplement 7—source data 4. [file elife-81123-fig1-figsupp7-data4.tiff]

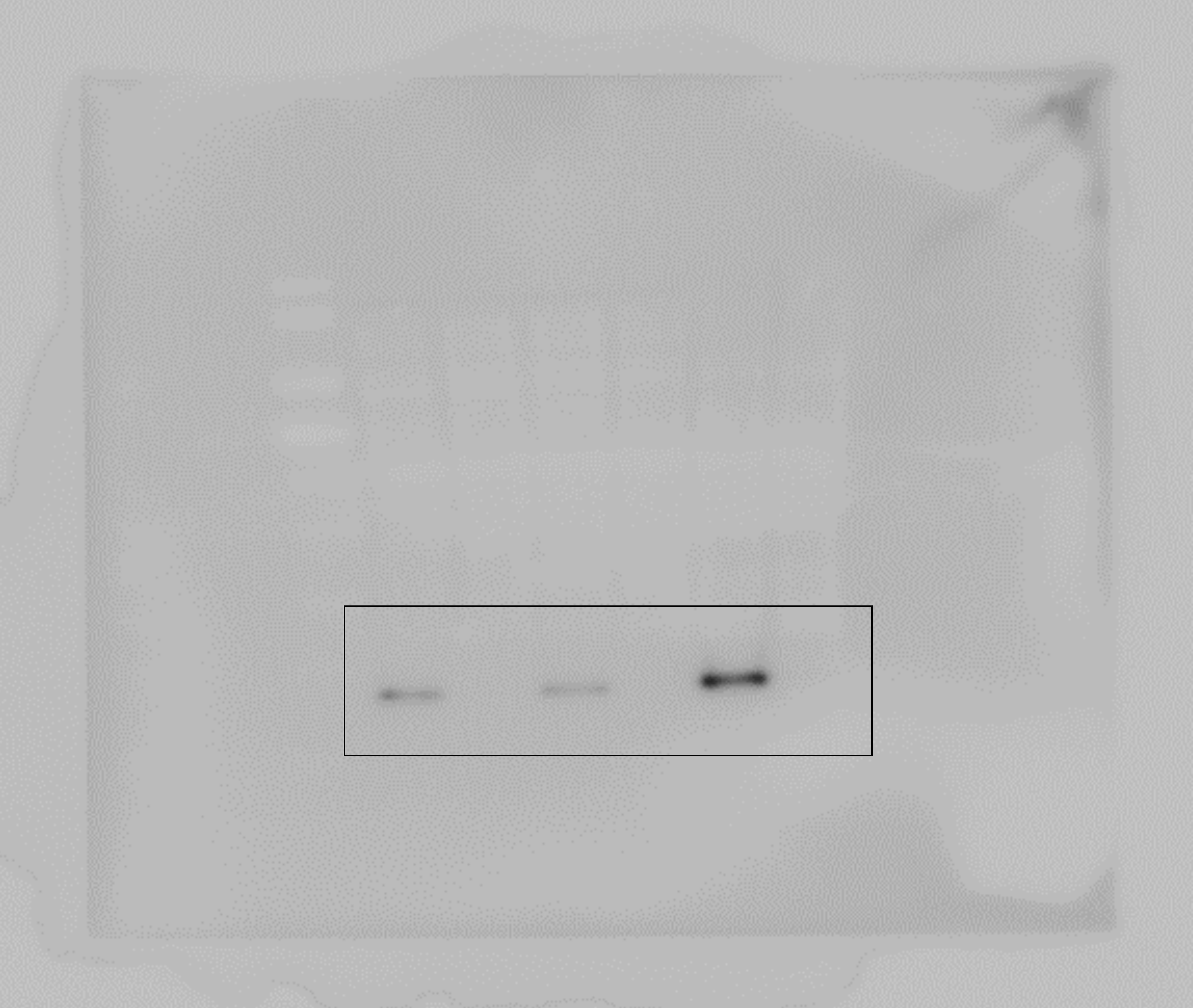

Supplement: Figure 1—figure supplement 7—source data 5. [file elife-81123-fig1-figsupp7-data5.tiff]

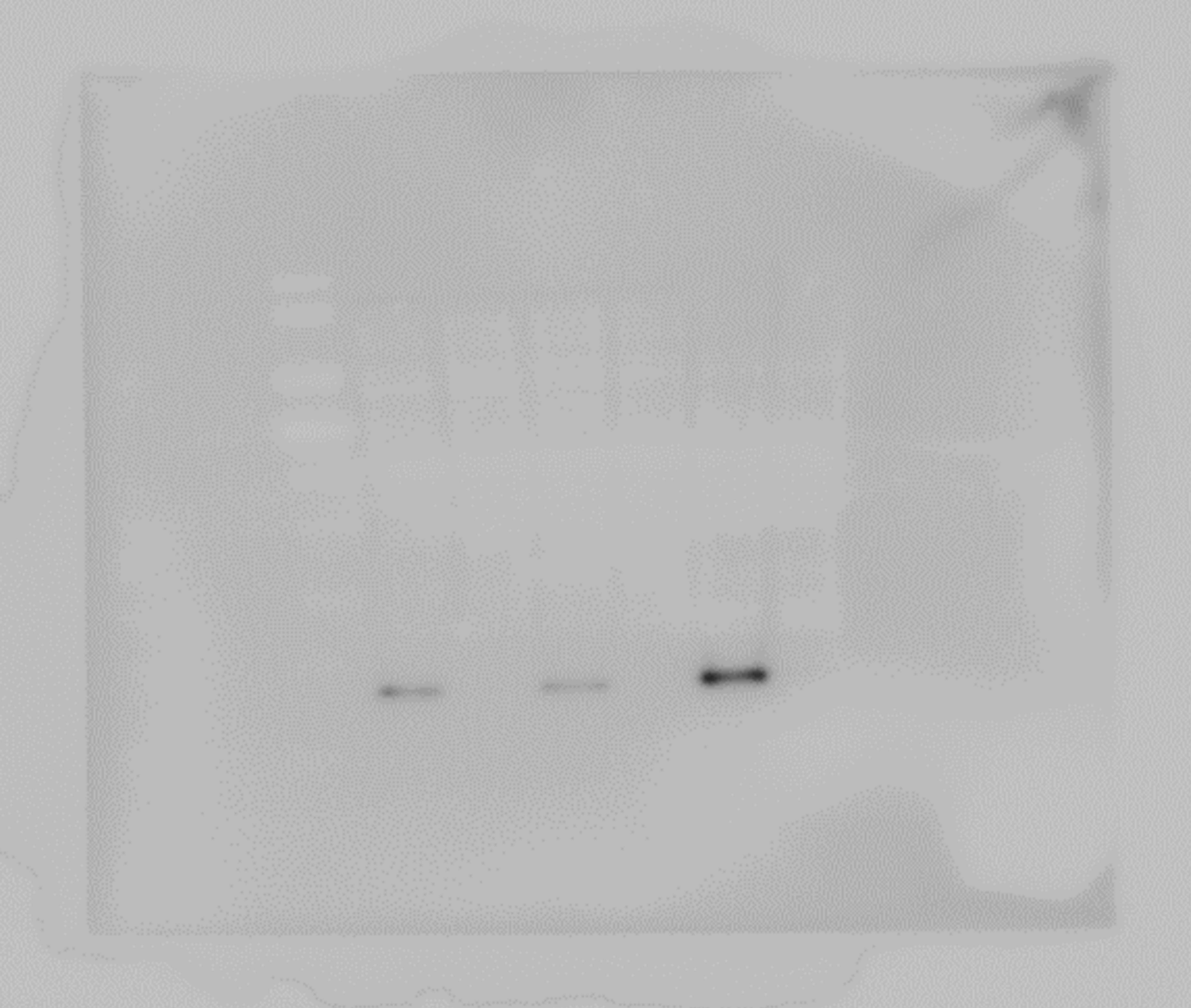

Supplement: Figure 1—figure supplement 7—source data 6. [file elife-81123-fig1-figsupp7-data6.tiff]

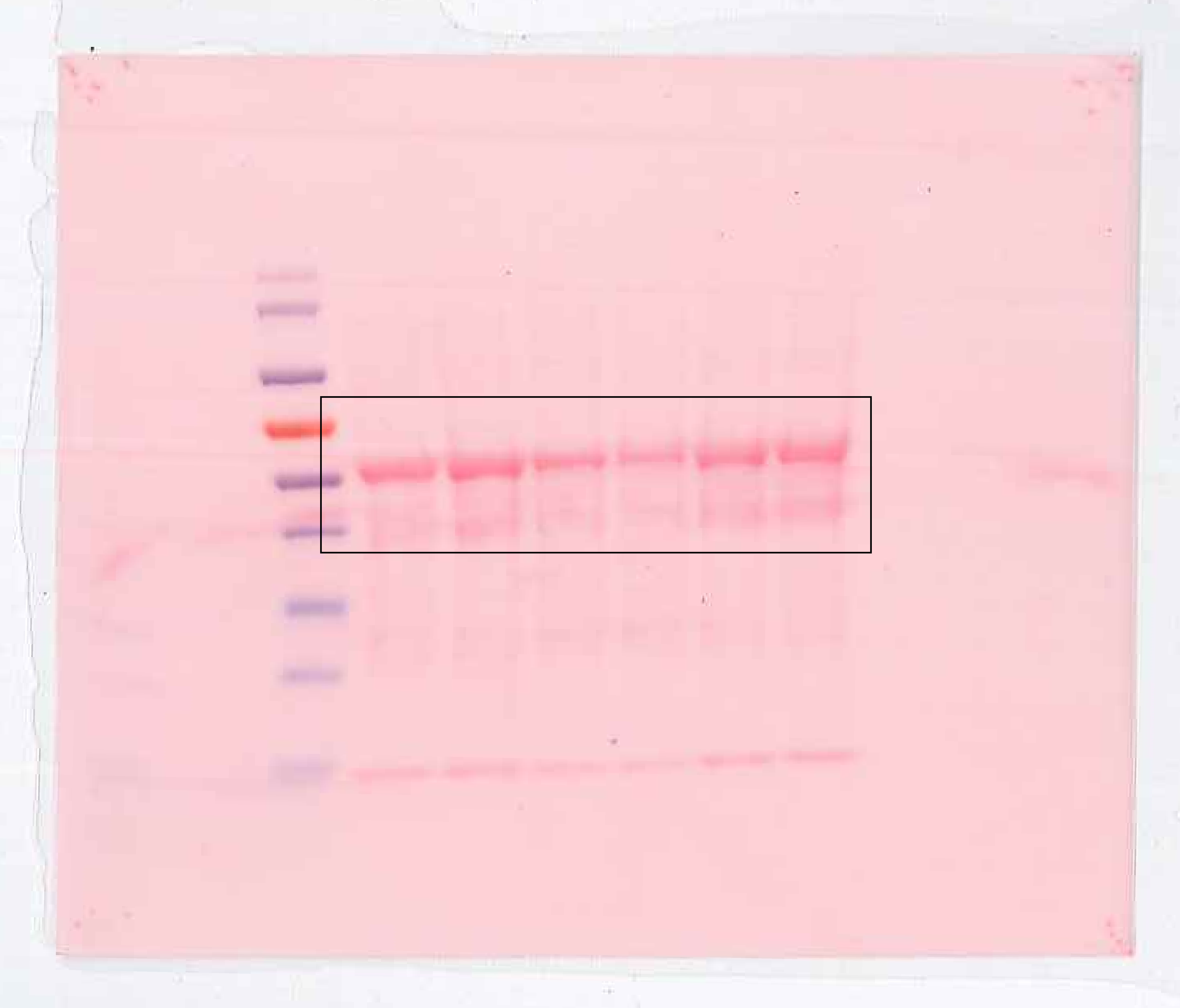

Supplement: Figure 1—figure supplement 7—source data 7. [file elife-81123-fig1-figsupp7-data7.tiff]

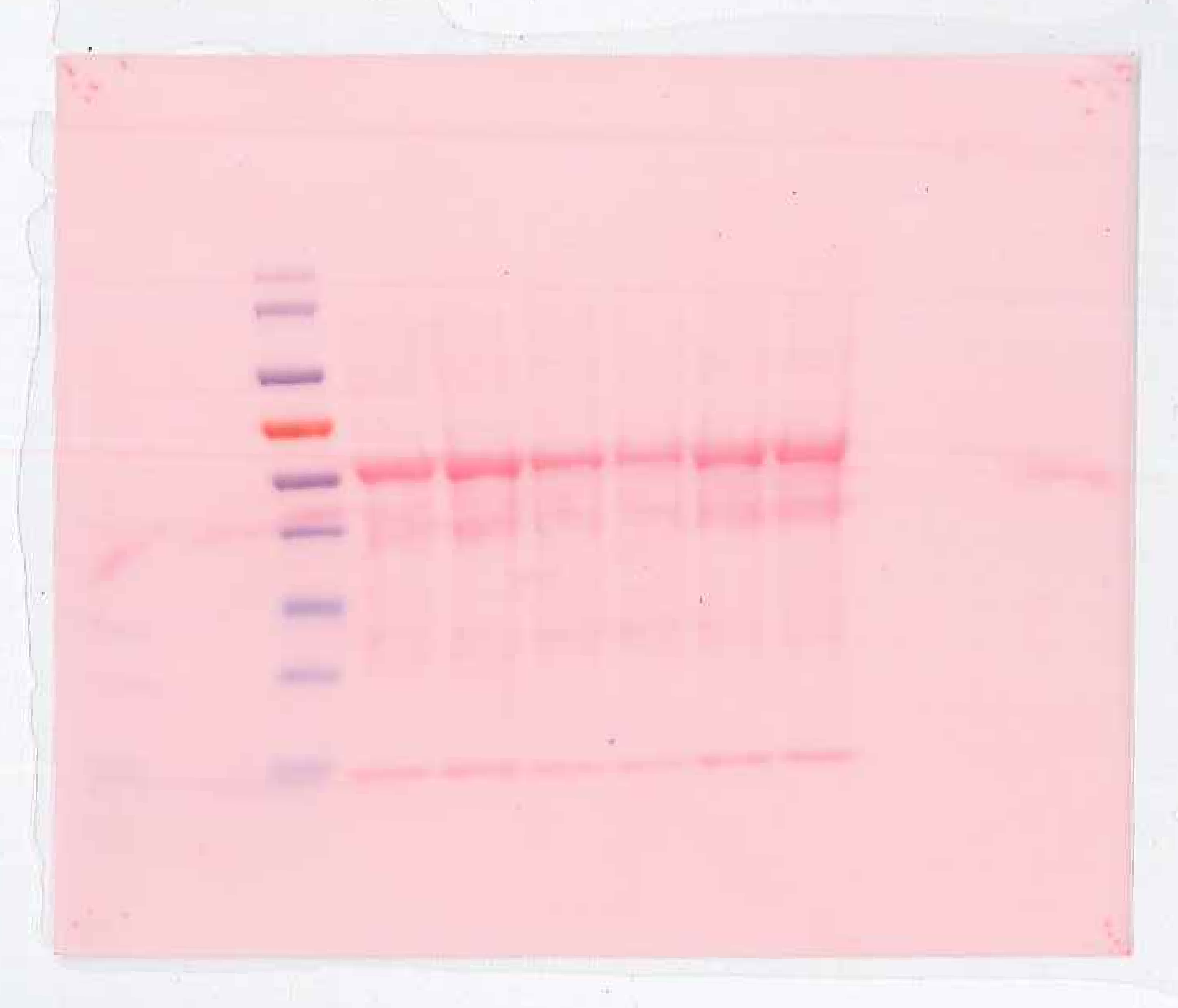

Supplement: Figure 1—figure supplement 7—source data 8. [file elife-81123-fig1-figsupp7-data8.tiff]

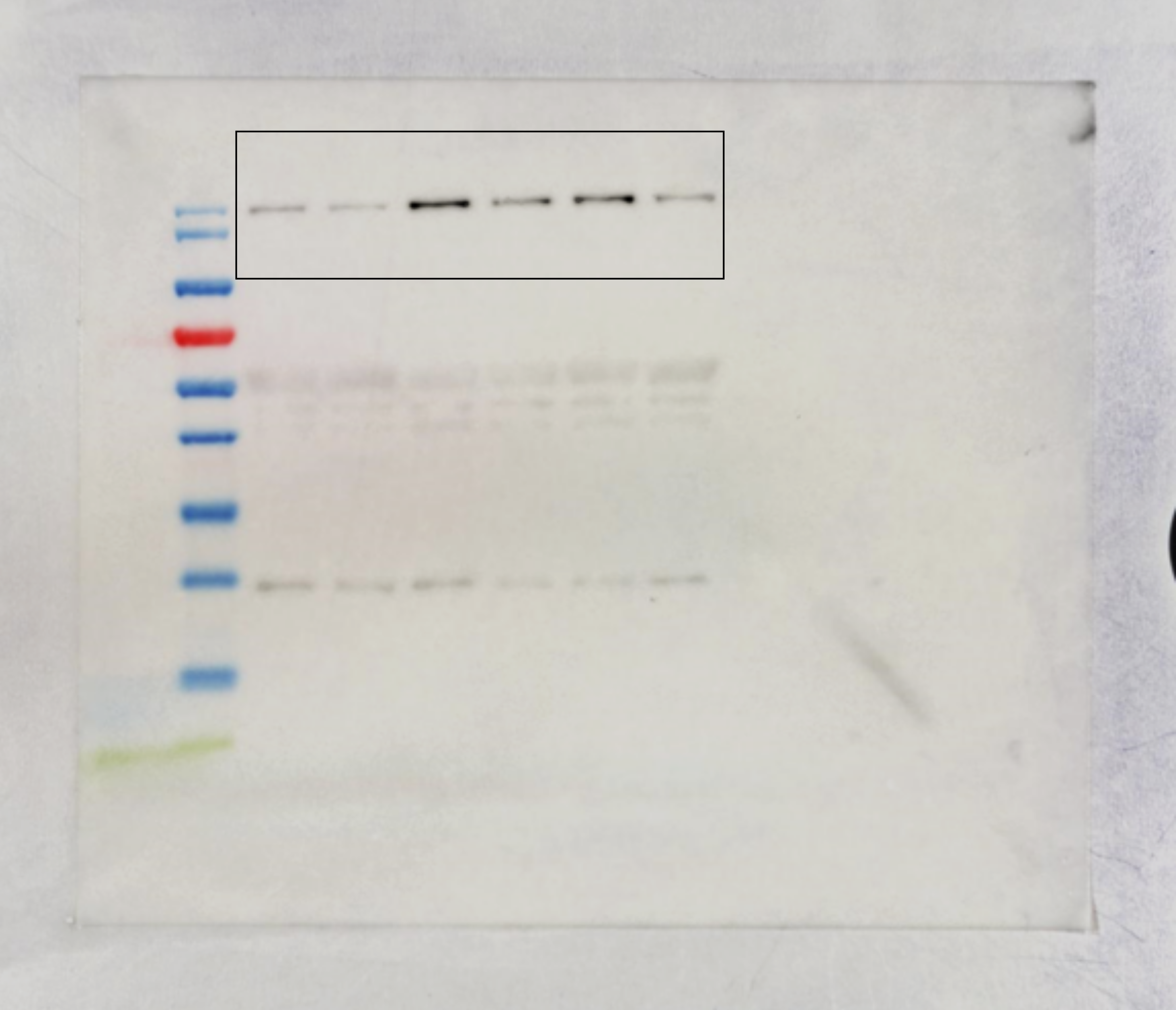

Supplement: Figure 1—figure supplement 7—source data 9. [file elife-81123-fig1-figsupp7-data9.tiff]

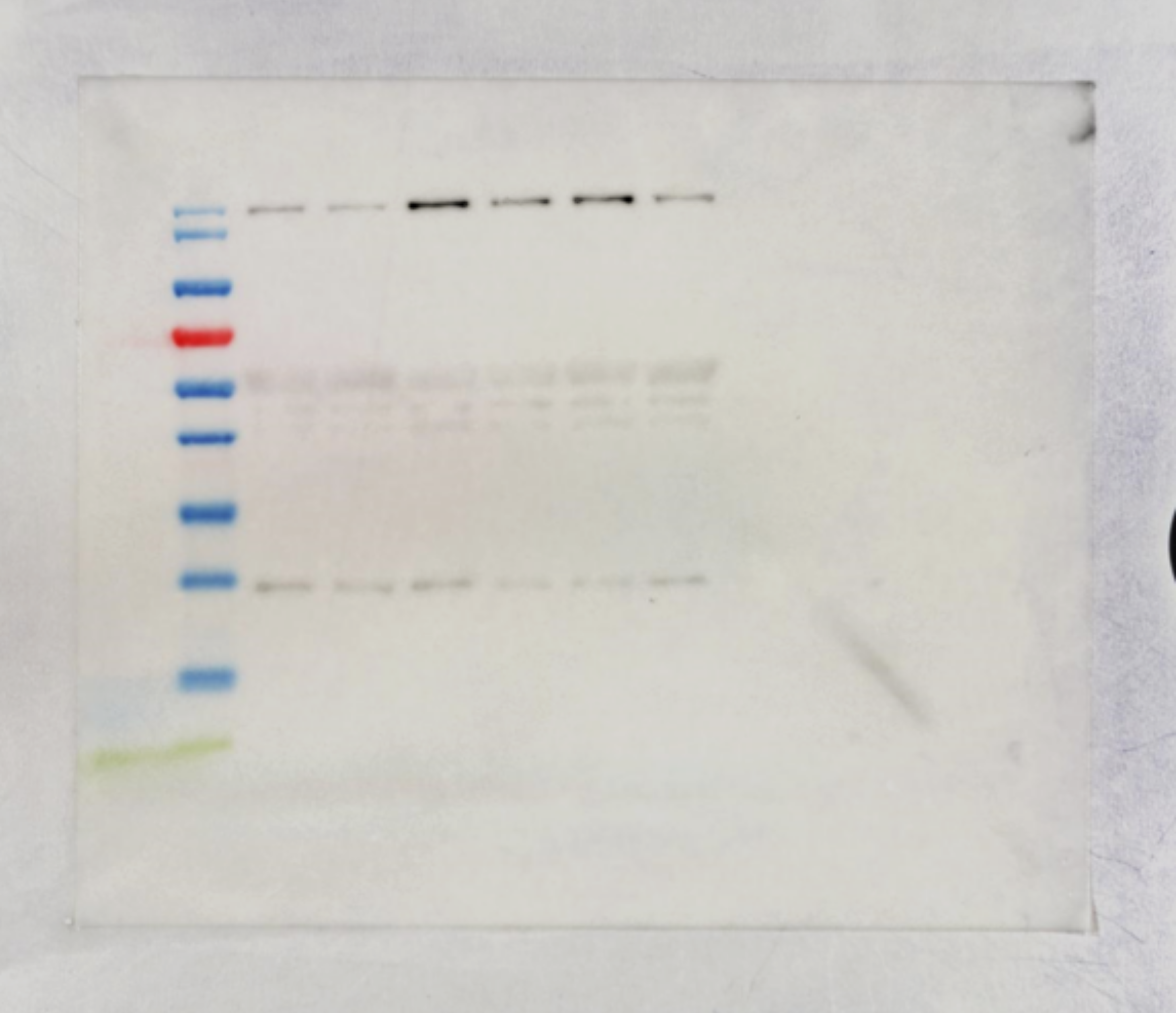

Supplement: Figure 1—figure supplement 7—source data 10. [file elife-81123-fig1-figsupp7-data10.tiff]

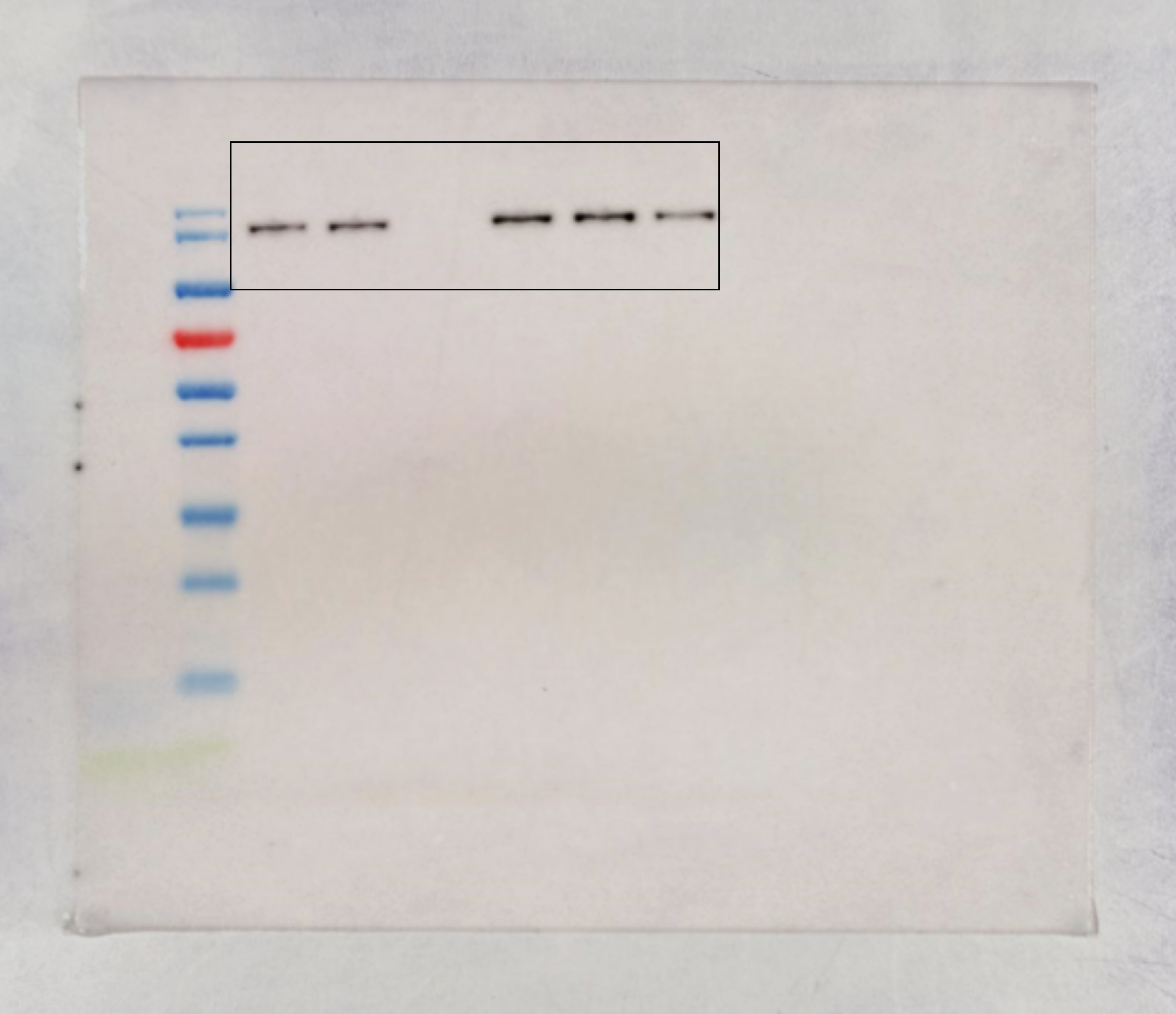

Supplement: Figure 1—figure supplement 7—source data 11. [file elife-81123-fig1-figsupp7-data11.tiff]

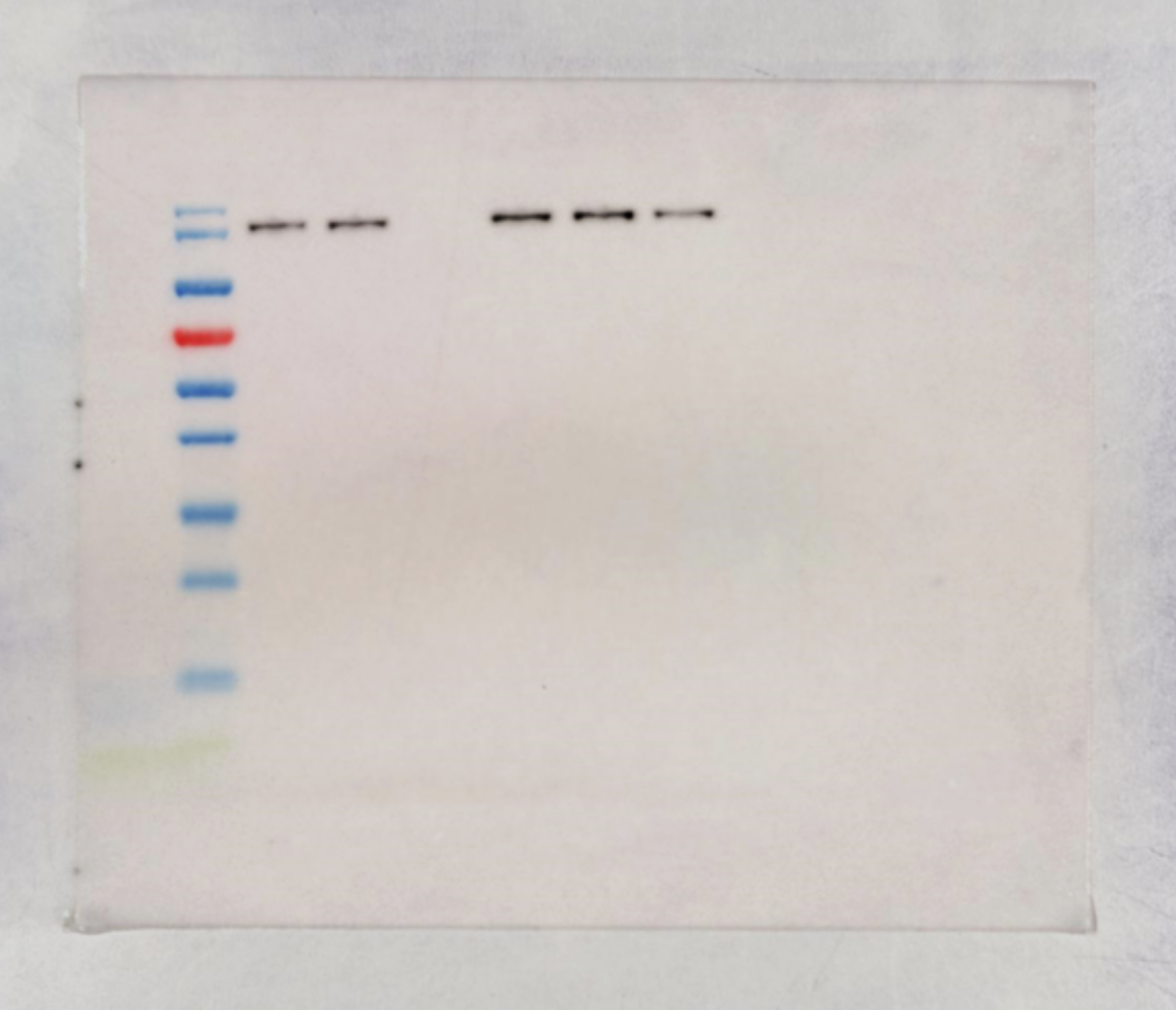

Supplement: Figure 1—figure supplement 7—source data 12. [file elife-81123-fig1-figsupp7-data12.tiff]

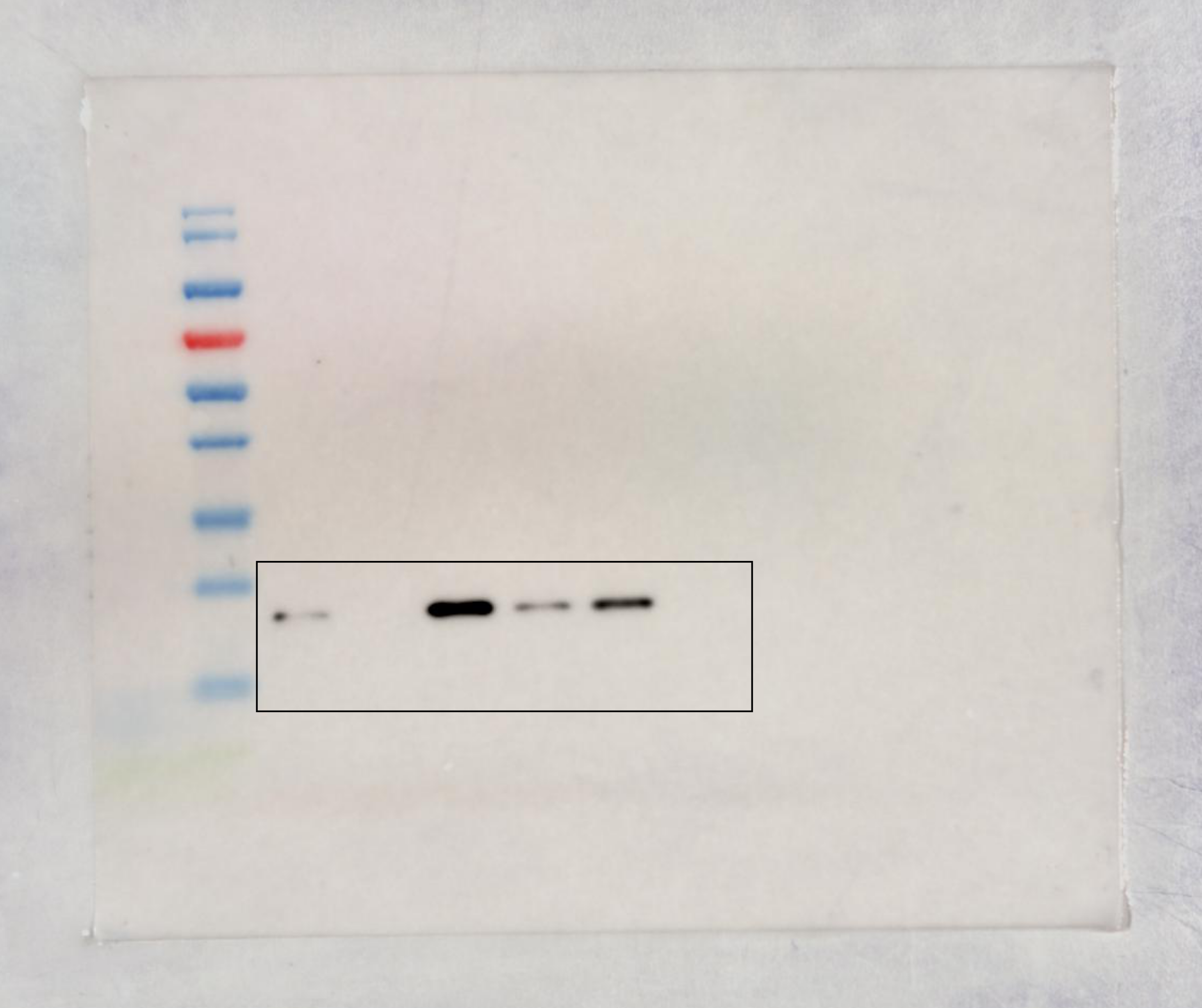

Supplement: Figure 1—figure supplement 7—source data 13. [file elife-81123-fig1-figsupp7-data13.tiff]

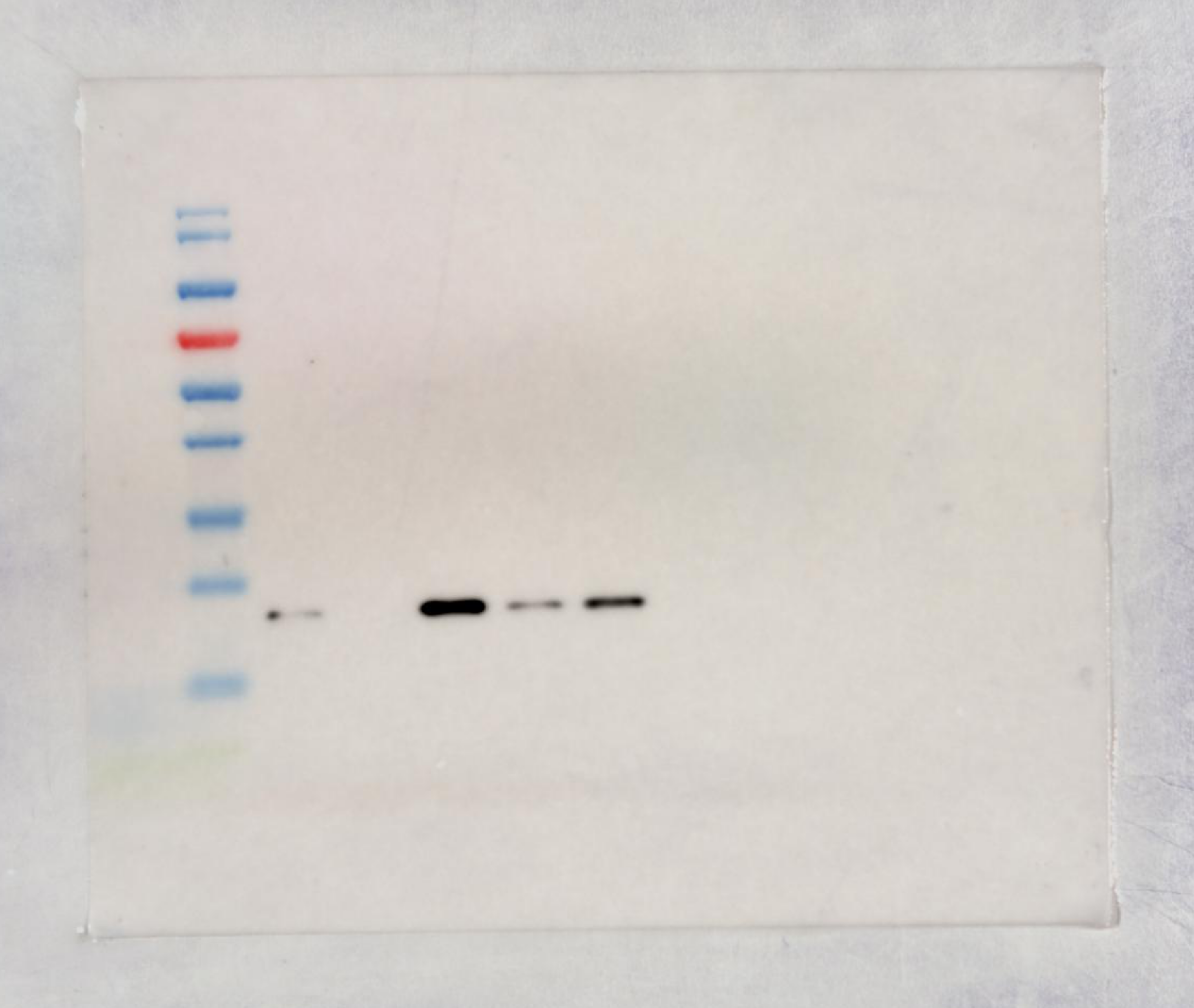

Supplement: Figure 1—figure supplement 7—source data 14. [file elife-81123-fig1-figsupp7-data14.tiff]

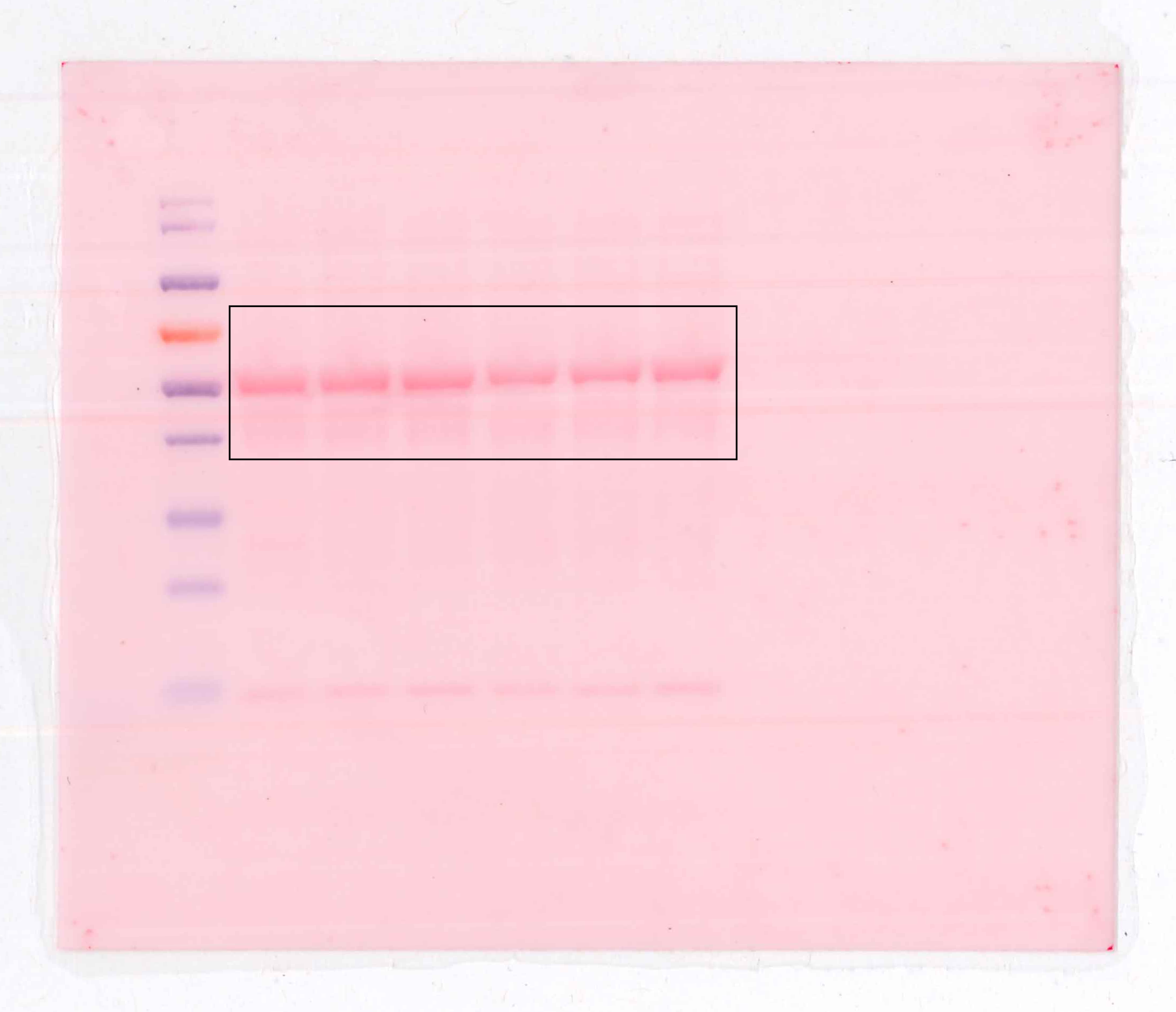

Supplement: Figure 1—figure supplement 7—source data 15. [file elife-81123-fig1-figsupp7-data15.tiff]

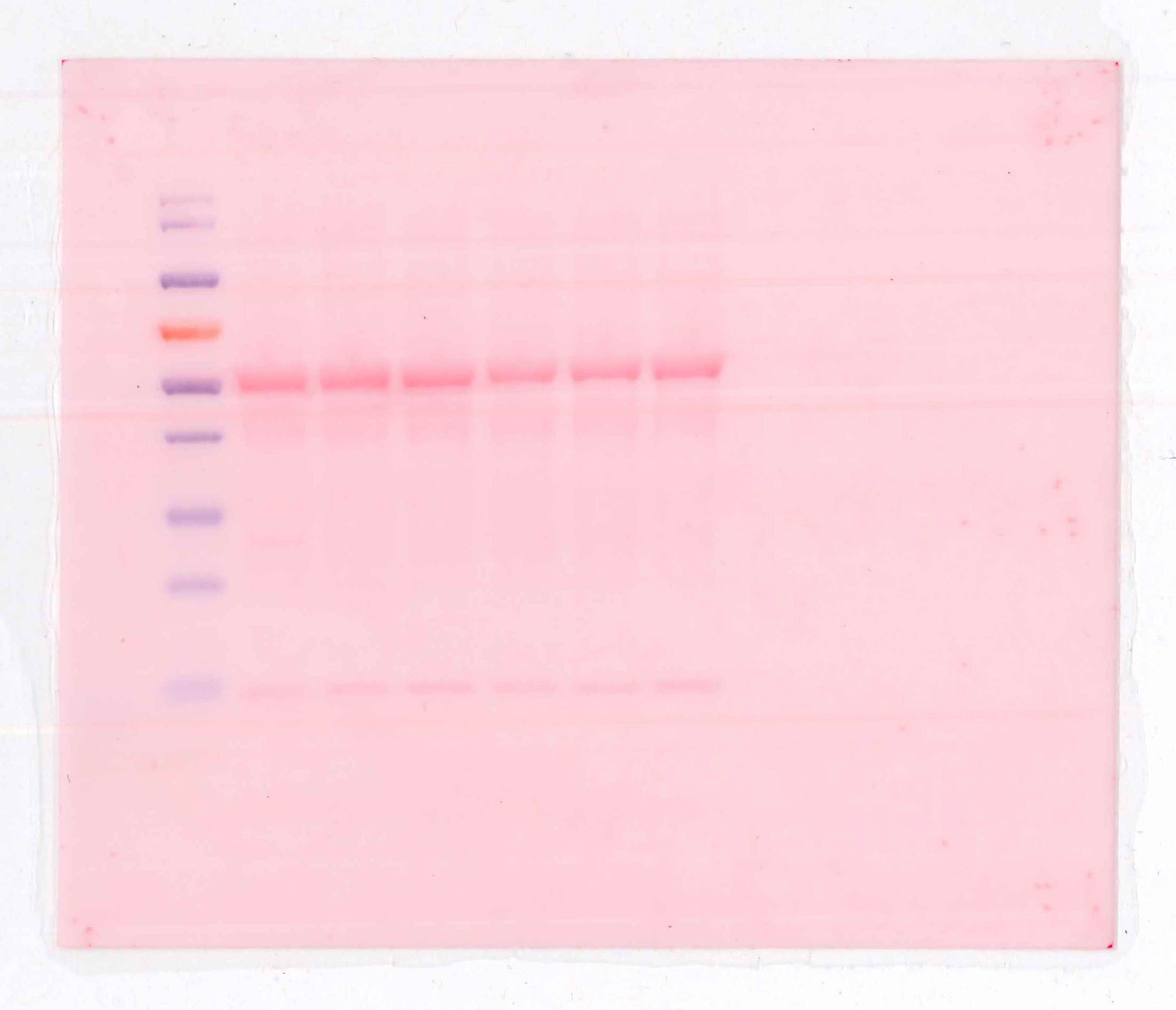

Supplement: Figure 1—figure supplement 7—source data 16. [file elife-81123-fig1-figsupp7-data16.tiff]

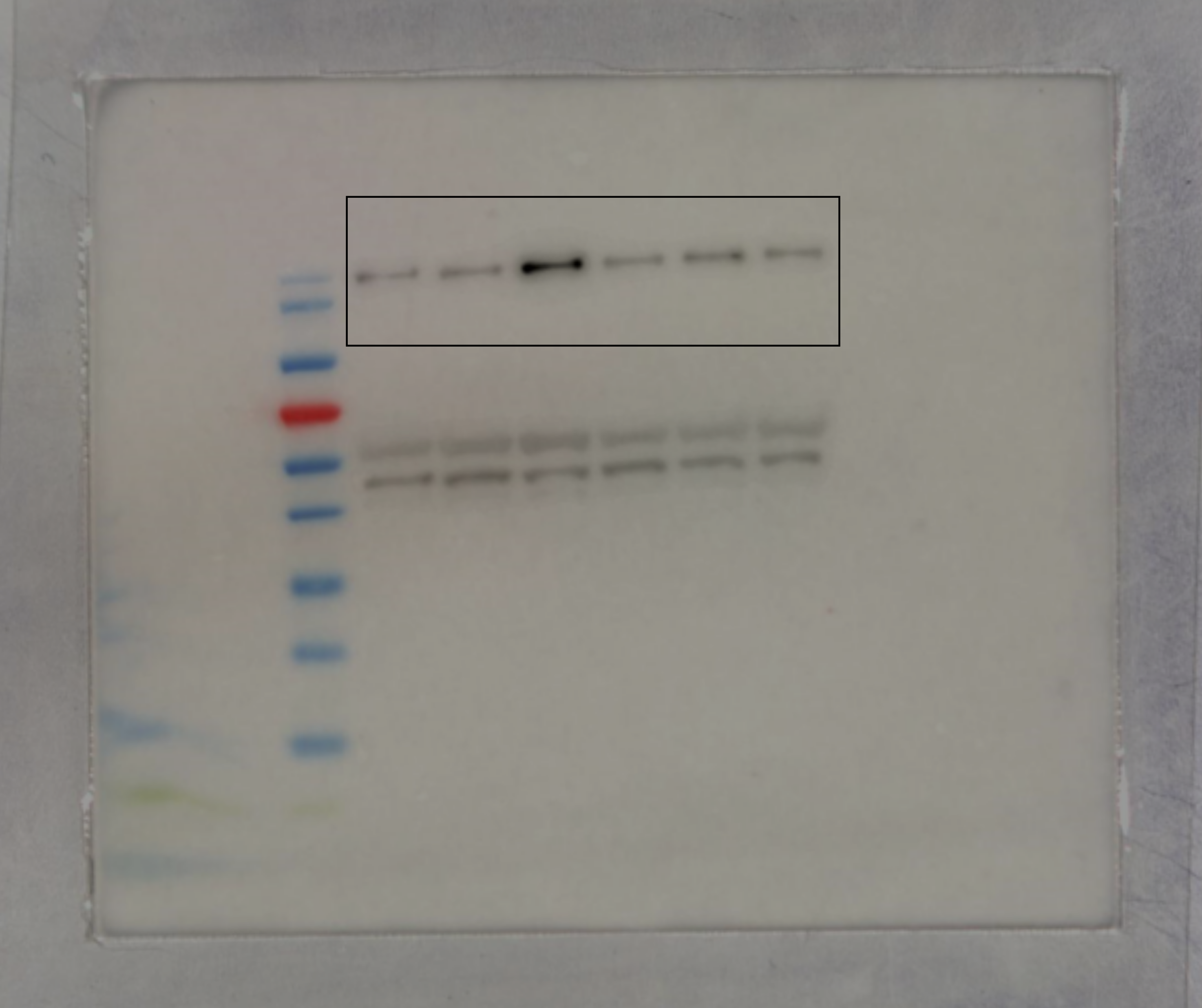

Supplement: Figure 1—figure supplement 7—source data 17. [file elife-81123-fig1-figsupp7-data17.tiff]

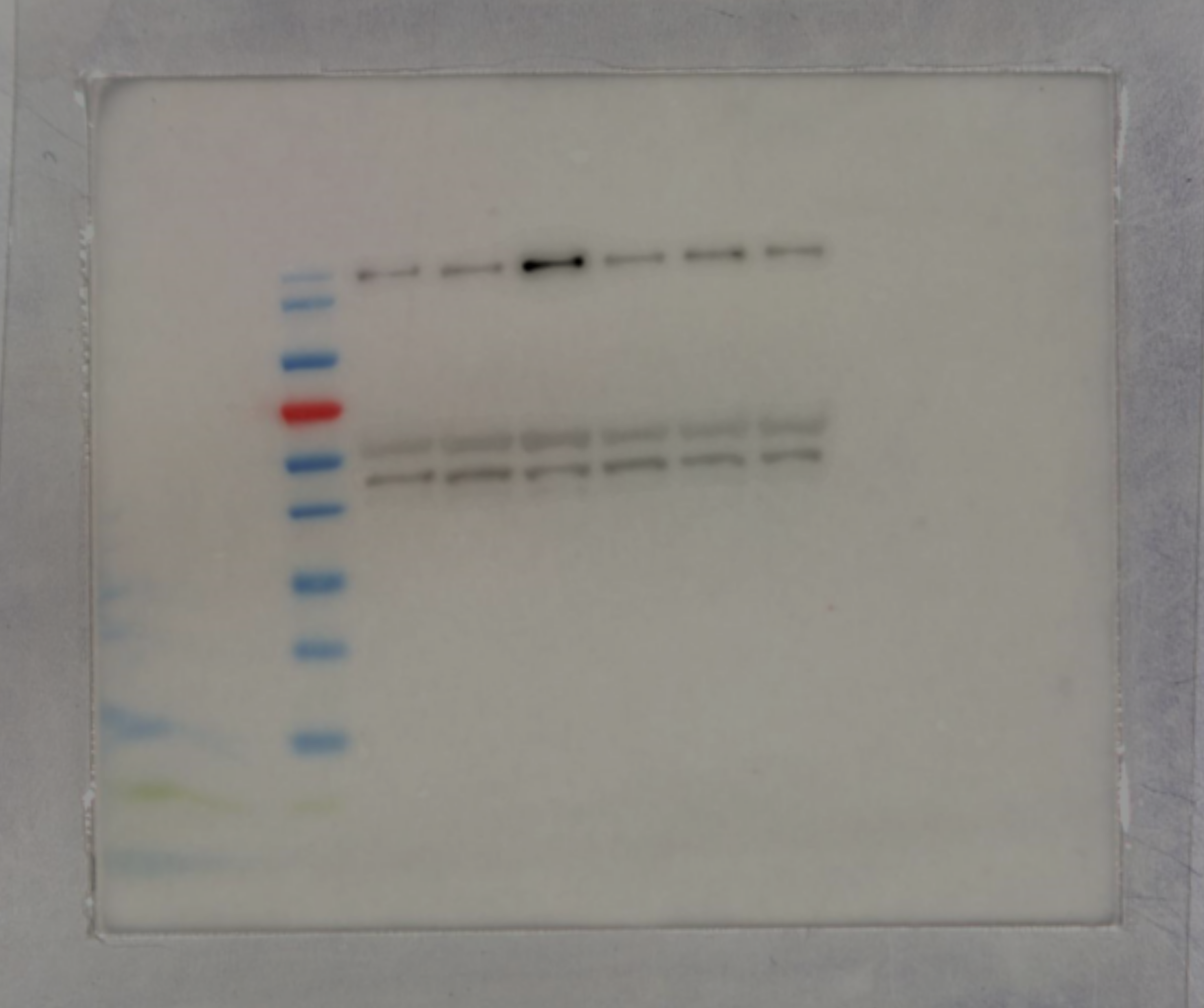

Supplement: Figure 1—figure supplement 7—source data 18. [file elife-81123-fig1-figsupp7-data18.tiff]

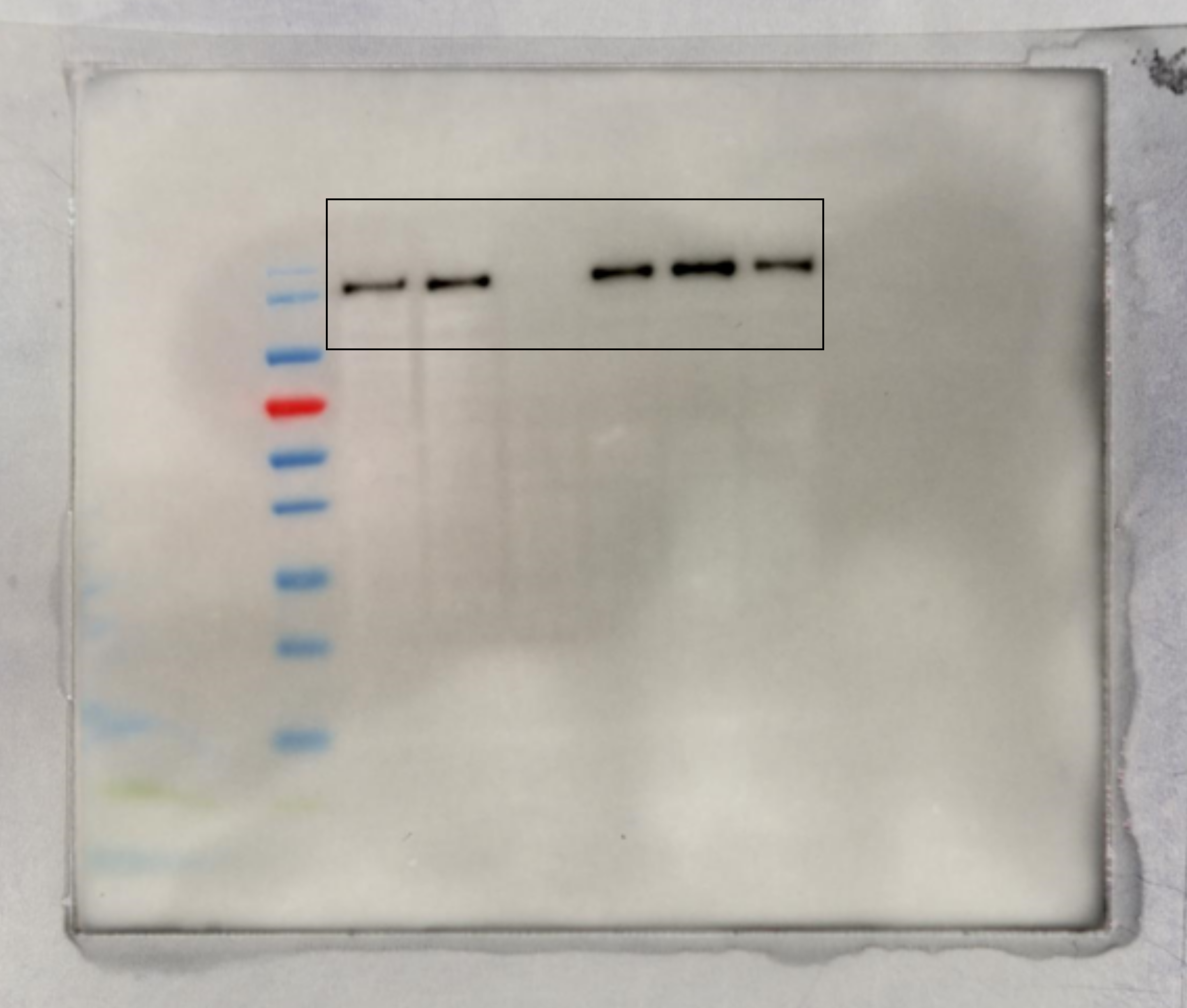

Supplement: Figure 1—figure supplement 7—source data 19. [file elife-81123-fig1-figsupp7-data19.tiff]

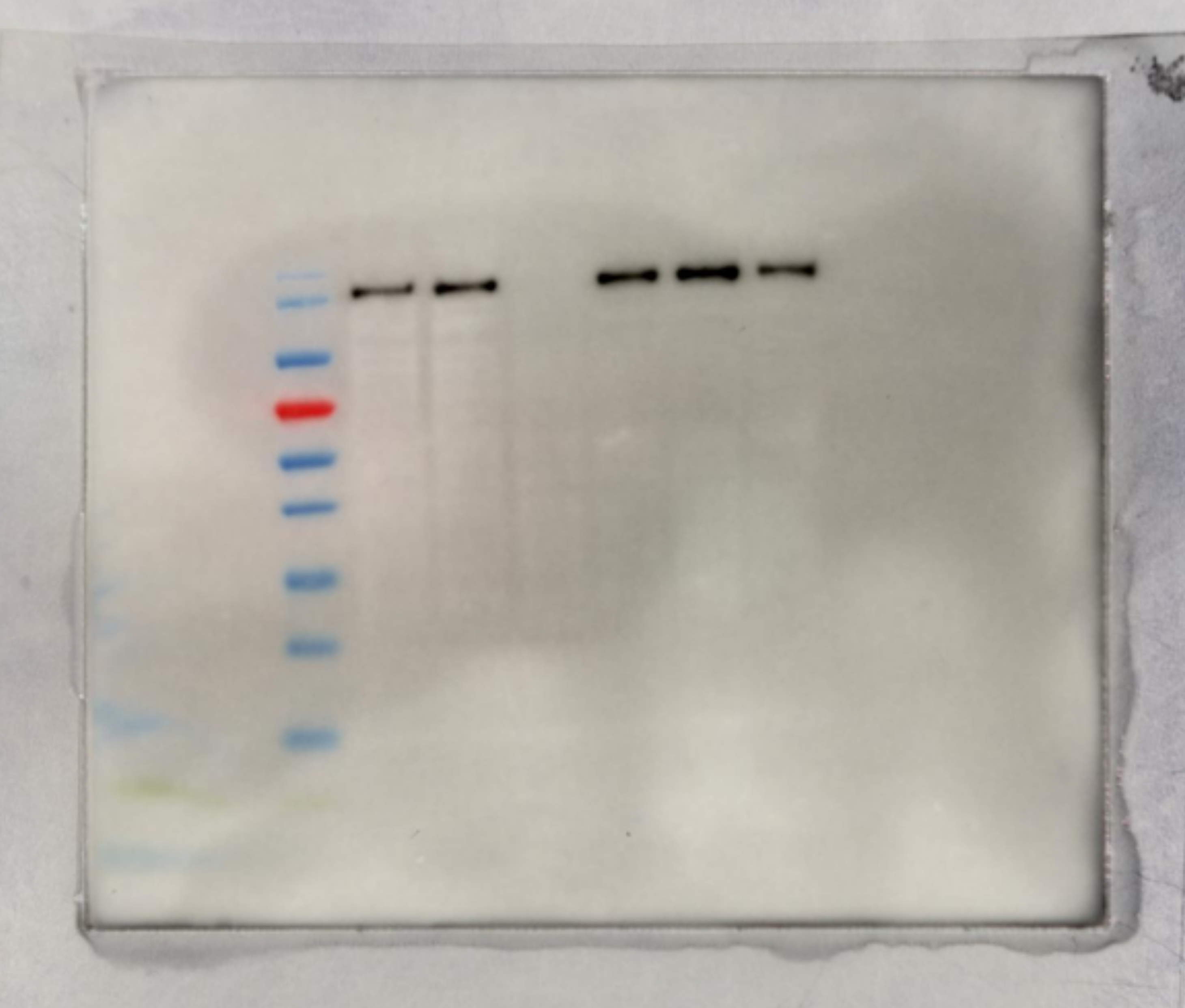

Supplement: Figure 1—figure supplement 7—source data 20. [file elife-81123-fig1-figsupp7-data20.tiff]

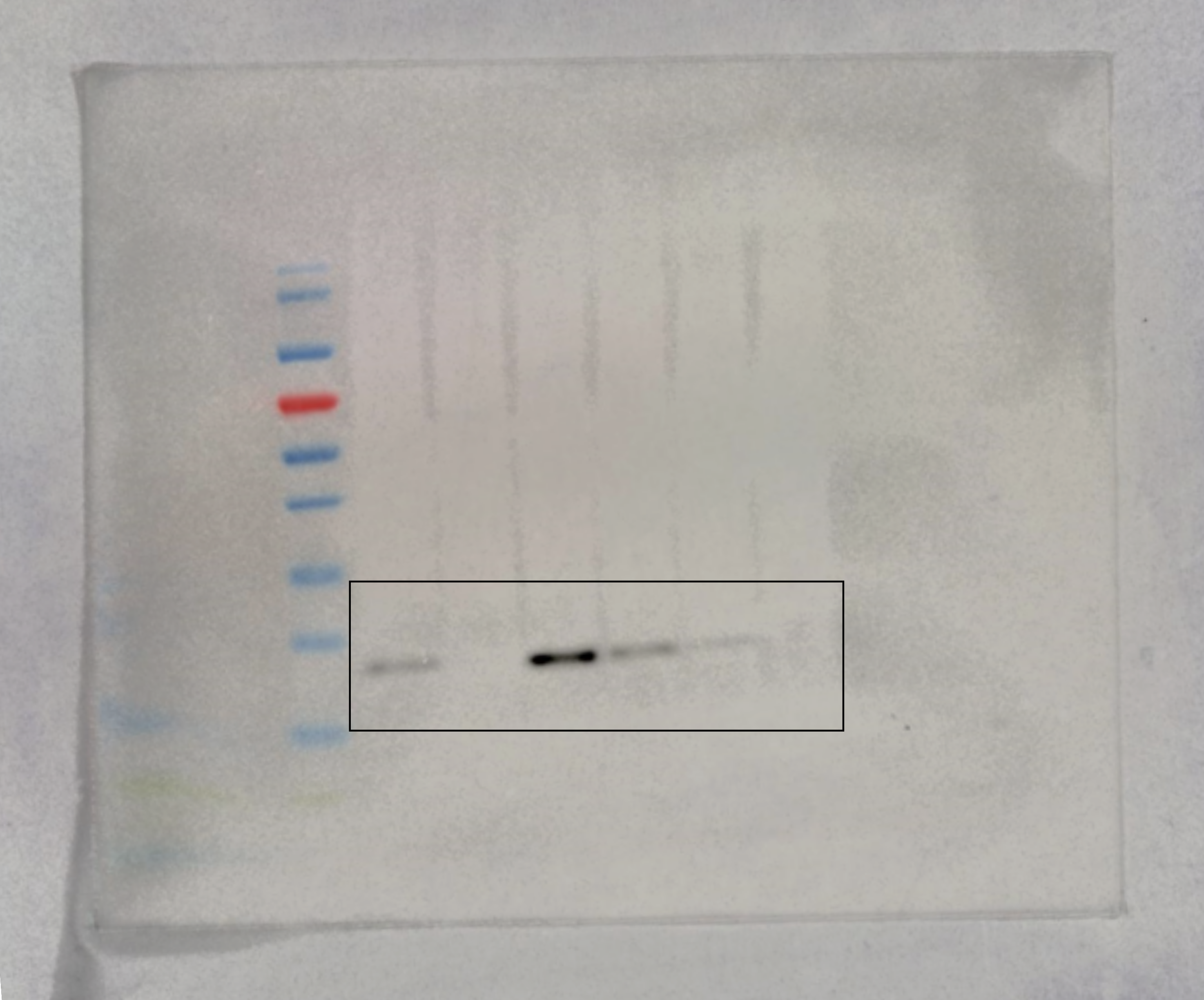

Supplement: Figure 1—figure supplement 7—source data 21. [file elife-81123-fig1-figsupp7-data21.tiff]

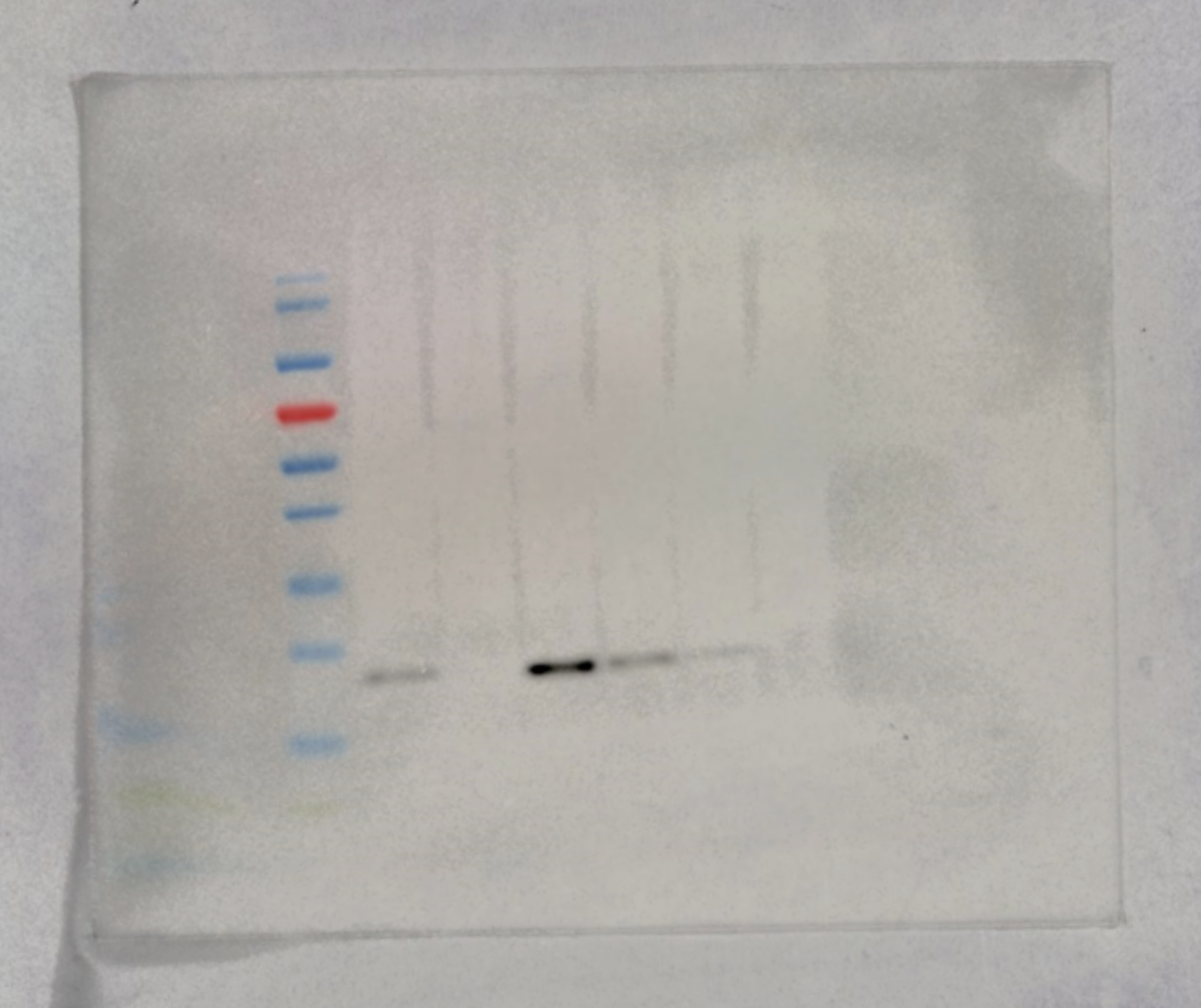

Supplement: Figure 1—figure supplement 7—source data 22. [file elife-81123-fig1-figsupp7-data22.tiff]

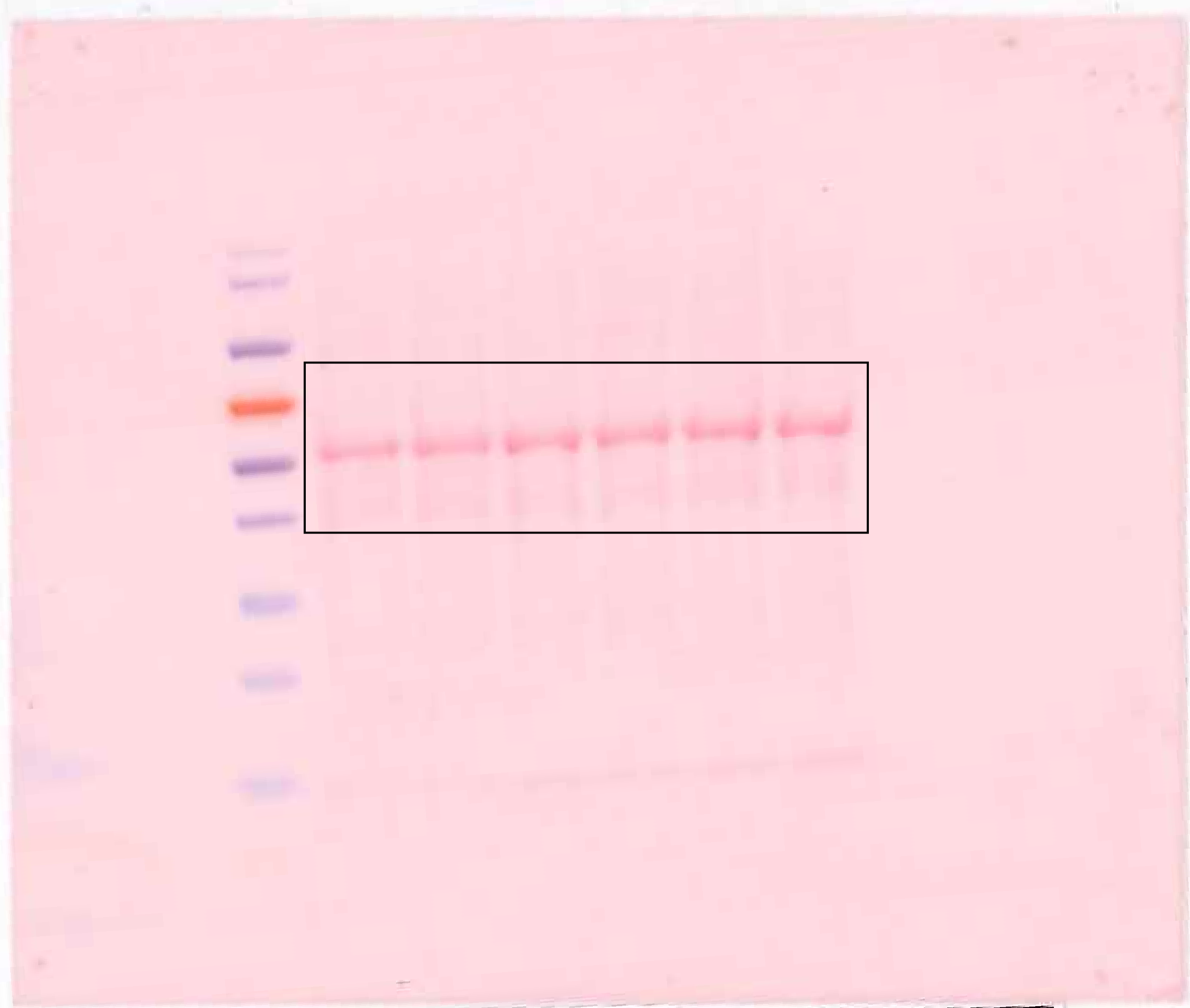

Supplement: Figure 1—figure supplement 7—source data 23. — Ponceau stain, with relevant bands labeled. [file elife-81123-fig1-figsupp7-data23.tiff]

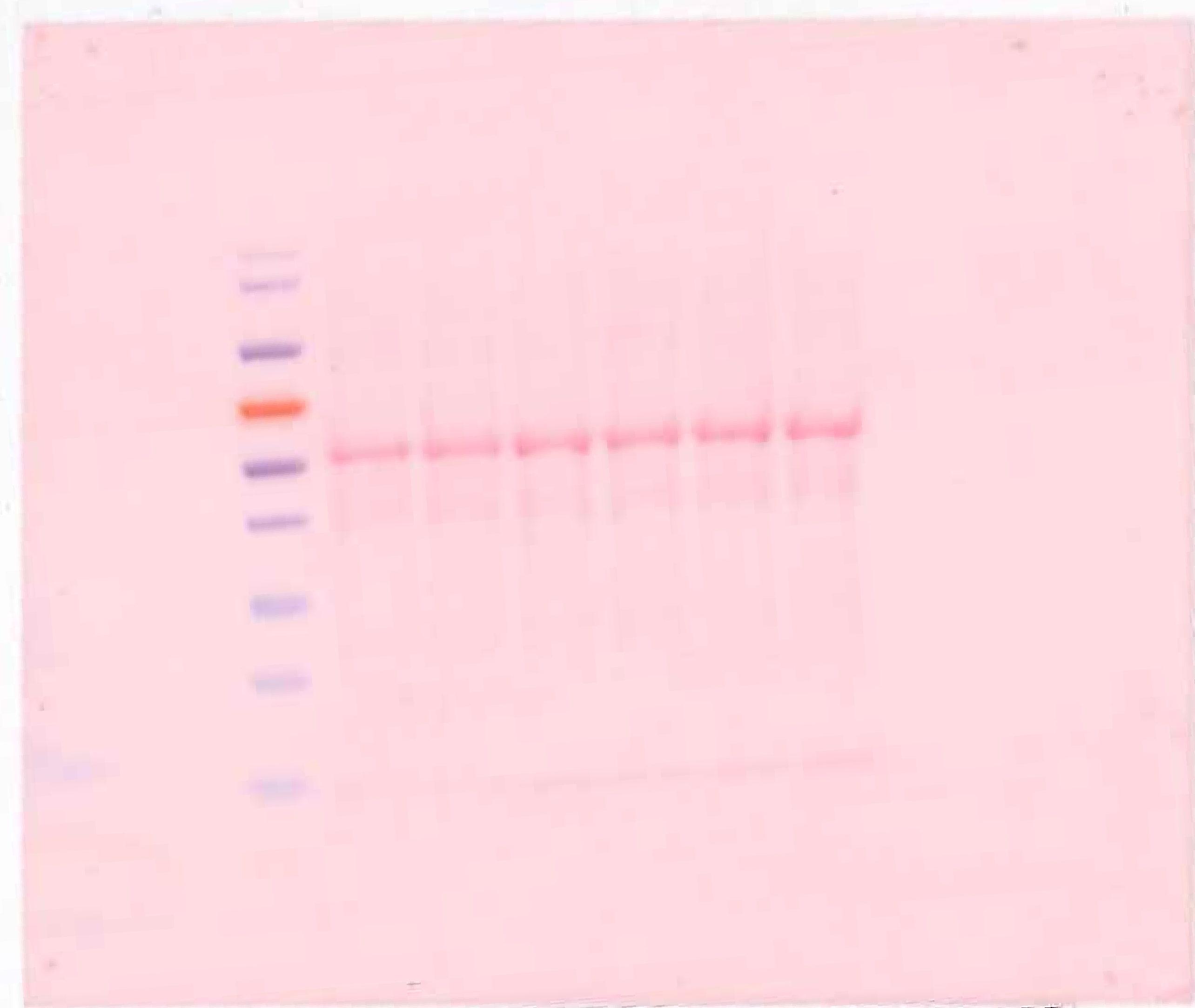

Supplement: Figure 1—figure supplement 7—source data 24. [file elife-81123-fig1-figsupp7-data24.tiff]

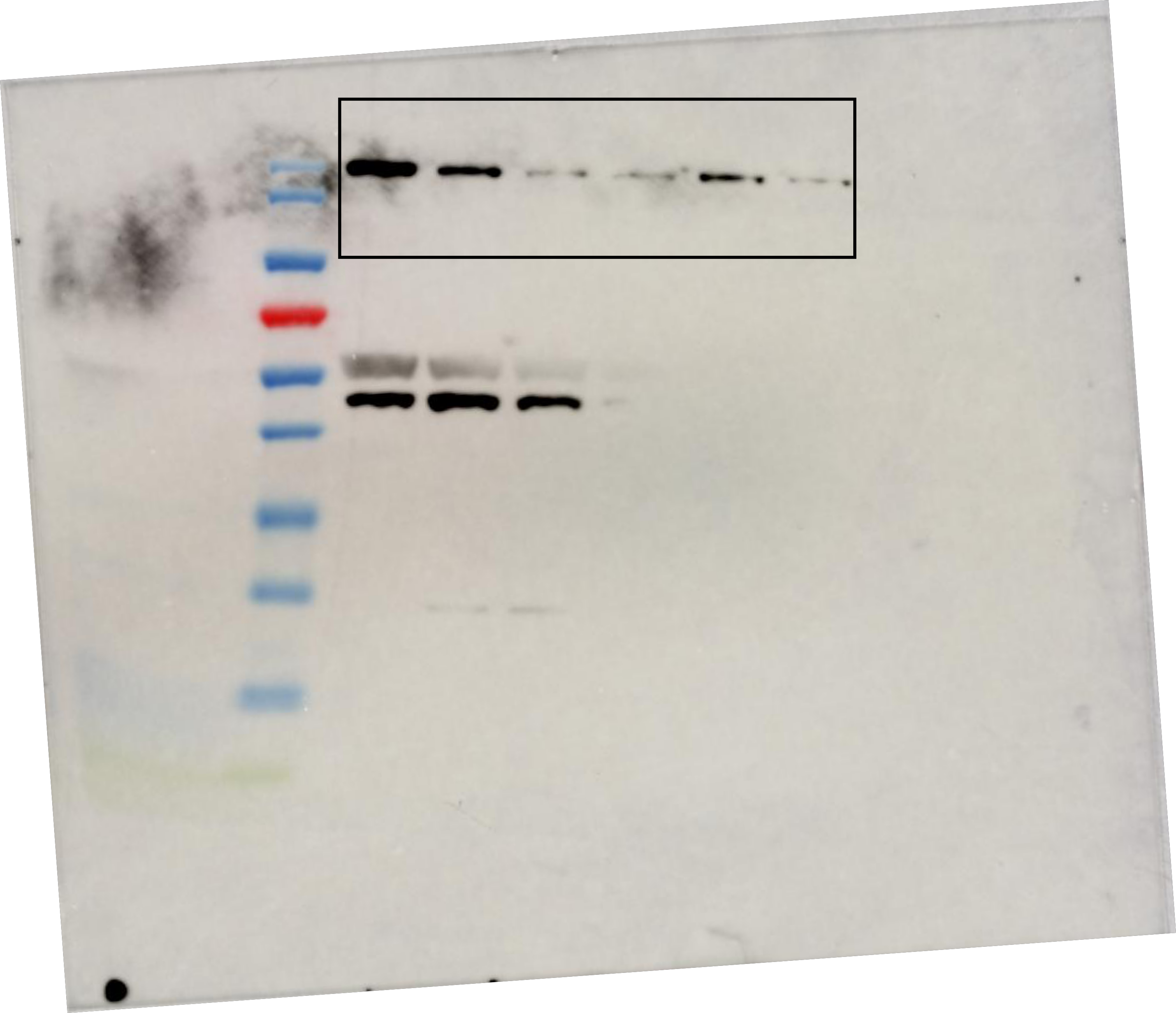

Supplement: Figure 2—figure supplement 1—source data 1. [file elife-81123-fig2-figsupp1-data1.tiff]

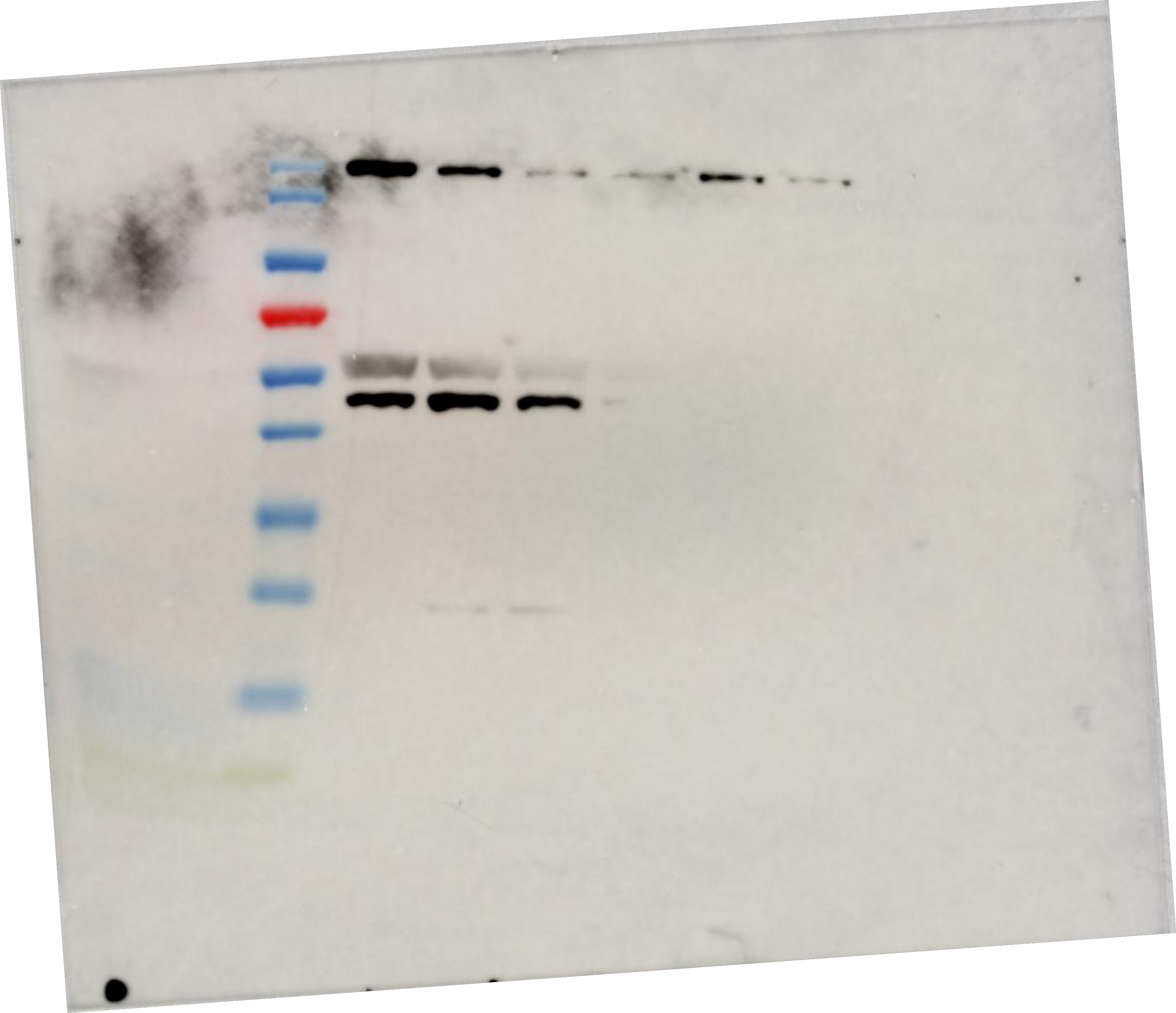

Supplement: Figure 2—figure supplement 1—source data 2. [file elife-81123-fig2-figsupp1-data2.tiff]

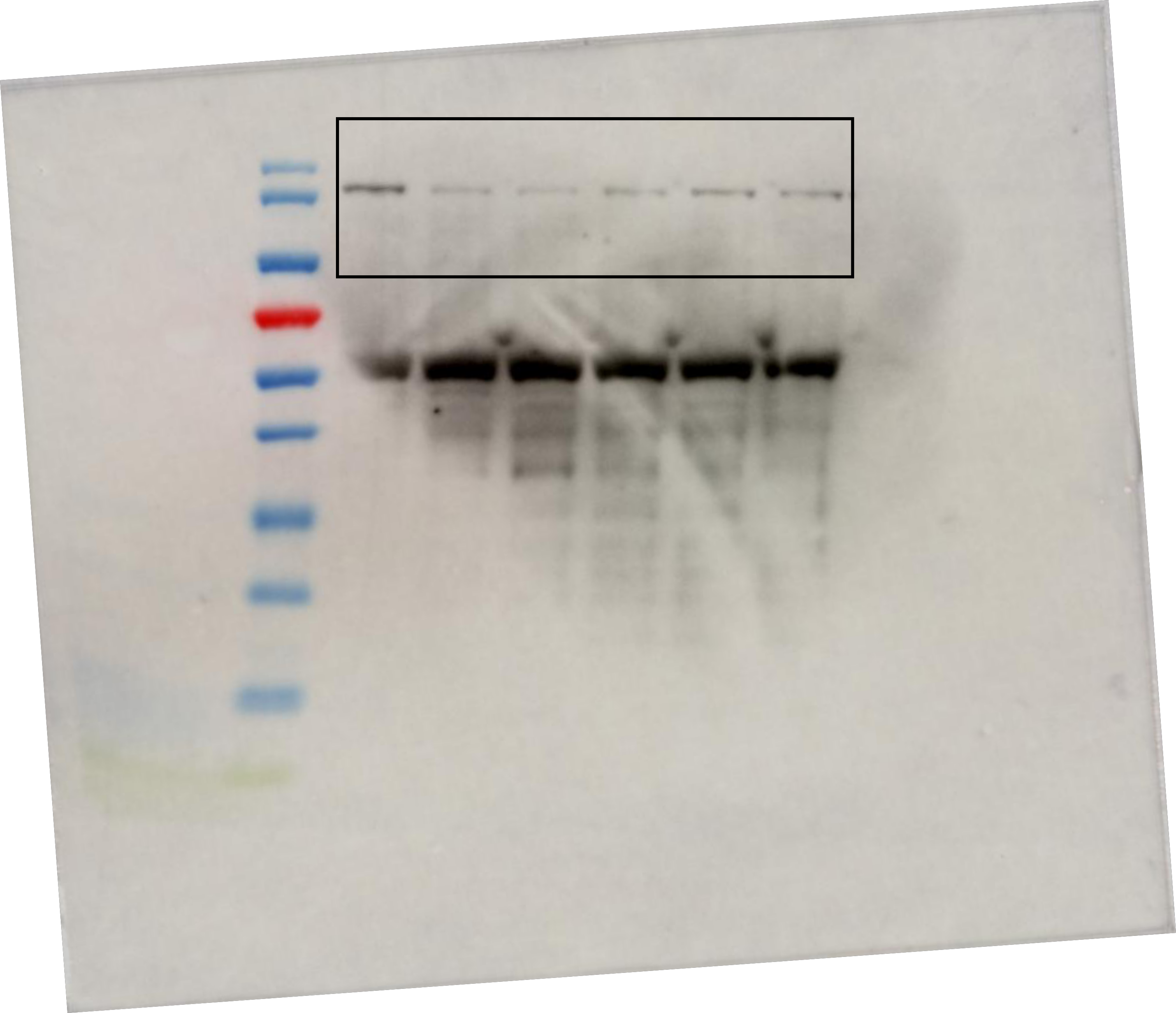

Supplement: Figure 2—figure supplement 1—source data 3. [file elife-81123-fig2-figsupp1-data3.tiff]

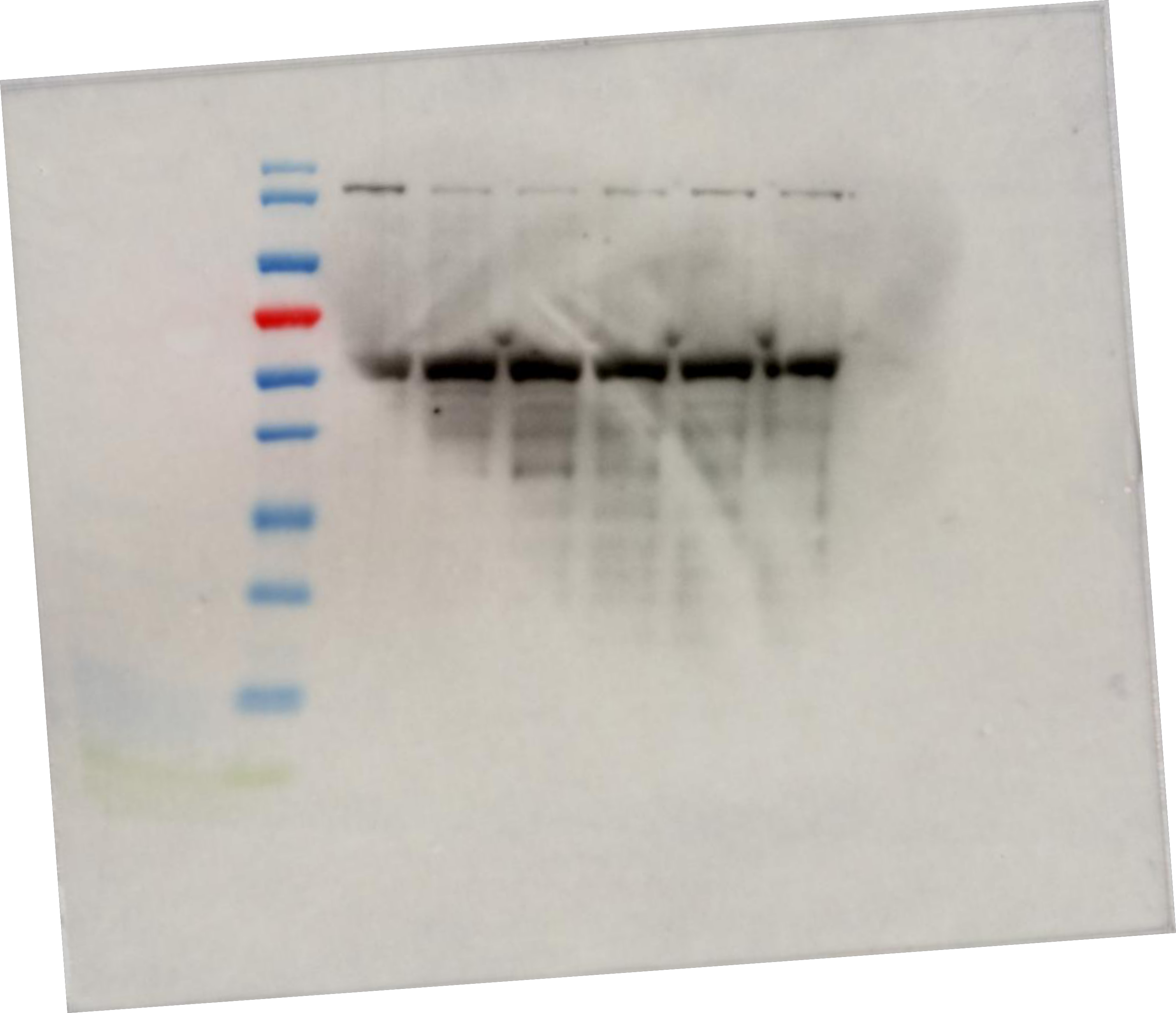

Supplement: Figure 2—figure supplement 1—source data 4. [file elife-81123-fig2-figsupp1-data4.tiff]

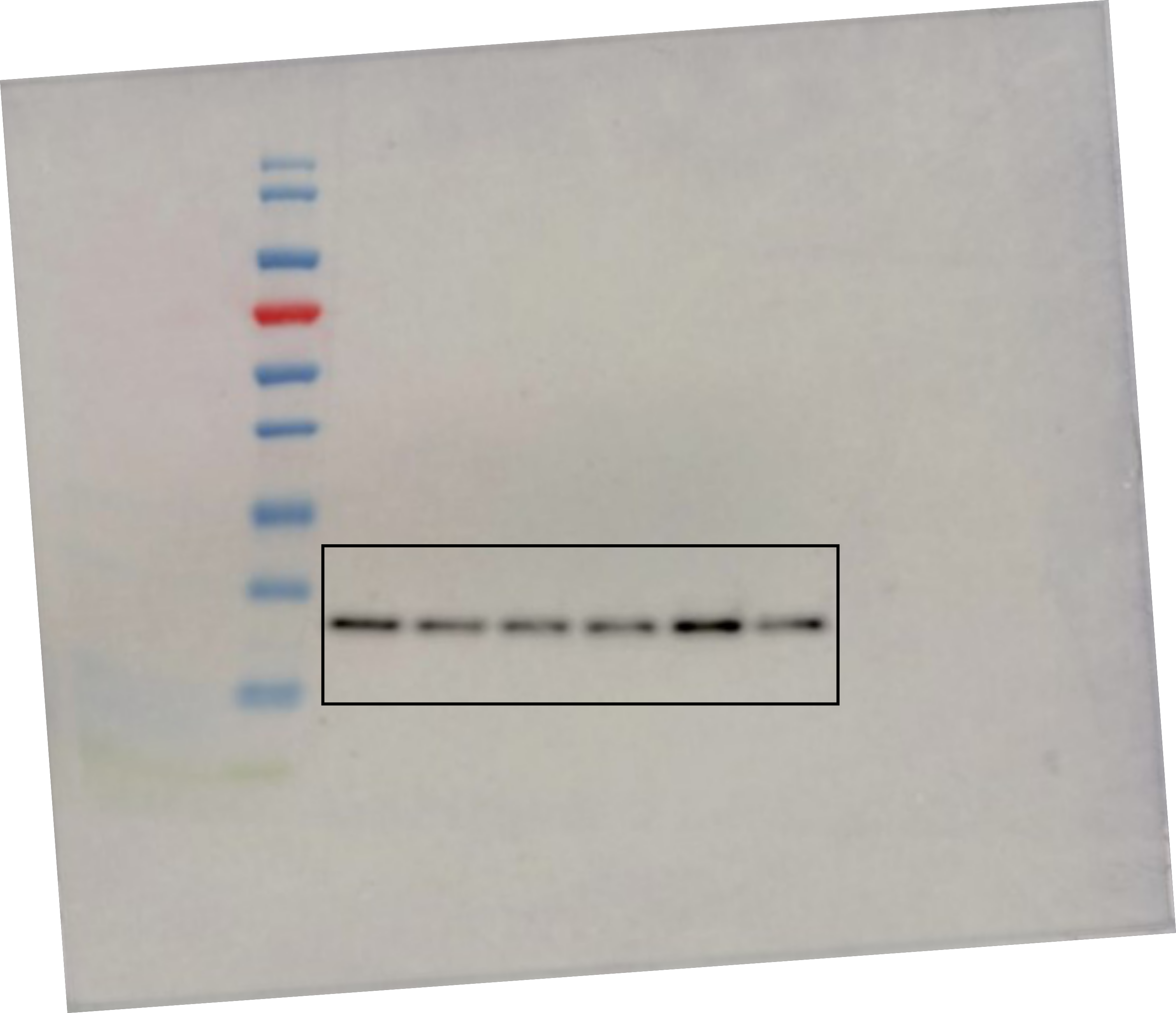

Supplement: Figure 2—figure supplement 1—source data 5. [file elife-81123-fig2-figsupp1-data5.tiff]

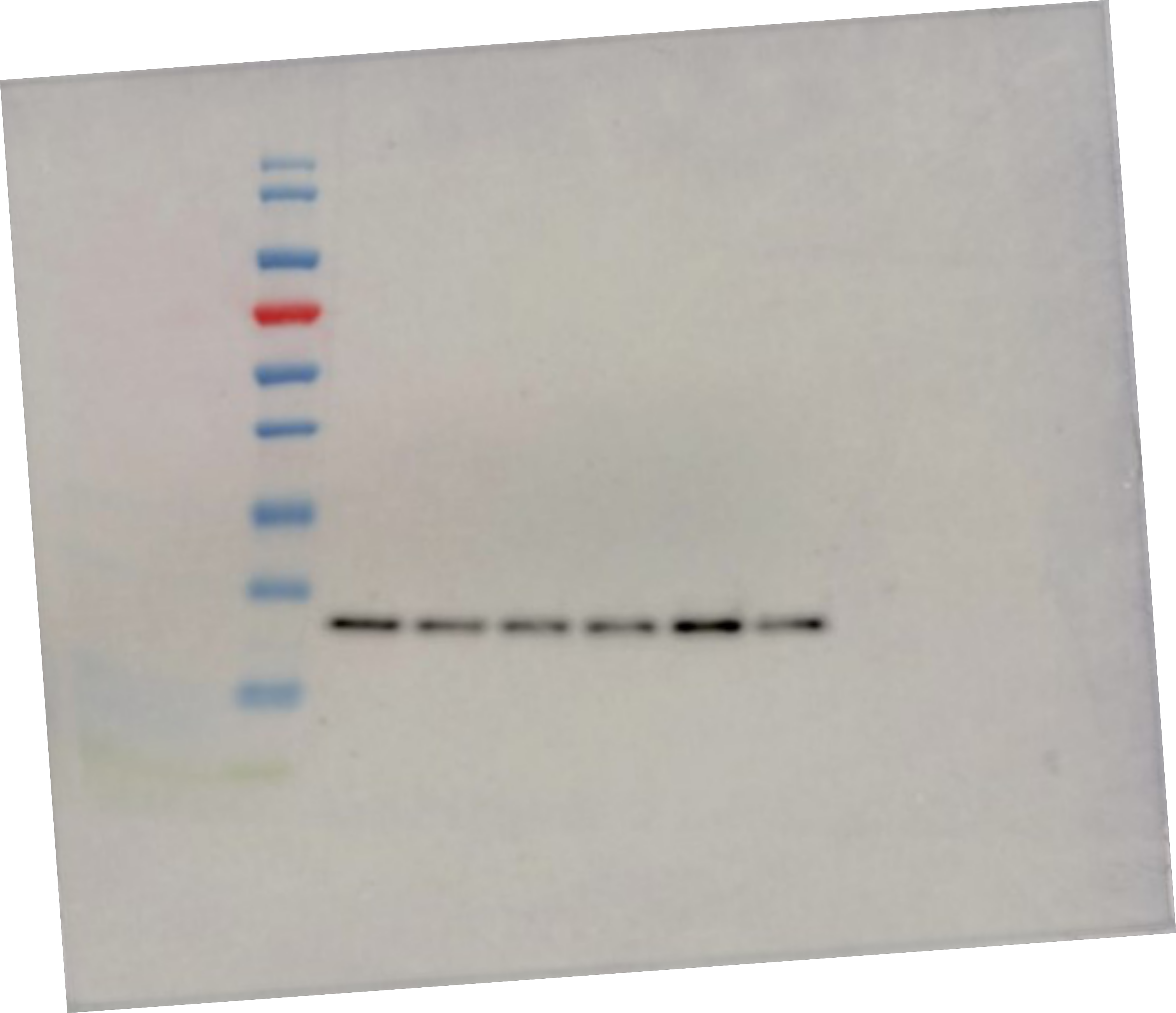

Supplement: Figure 2—figure supplement 1—source data 6. [file elife-81123-fig2-figsupp1-data6.tiff]

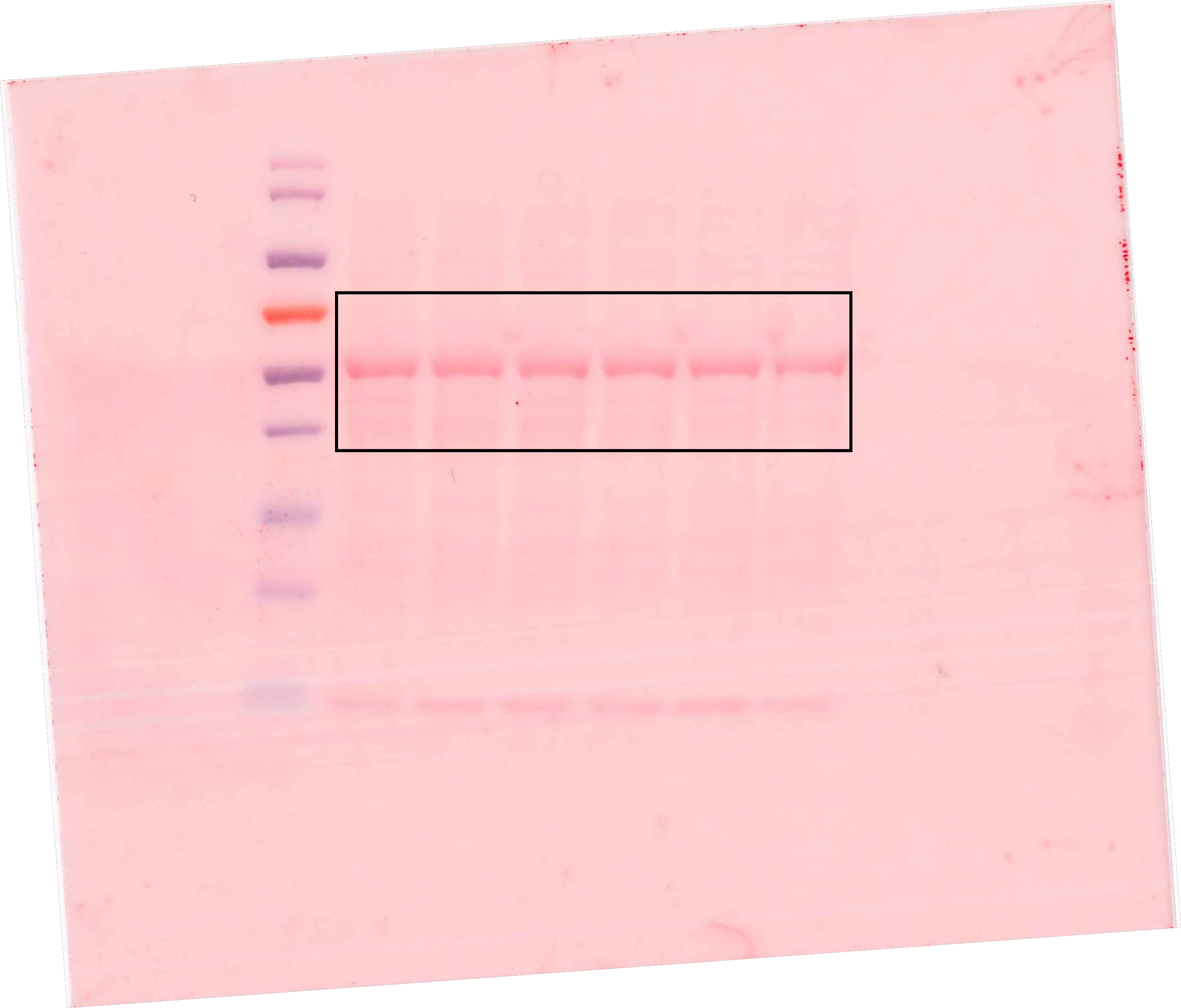

Supplement: Figure 2—figure supplement 1—source data 7. [file elife-81123-fig2-figsupp1-data7.tiff]

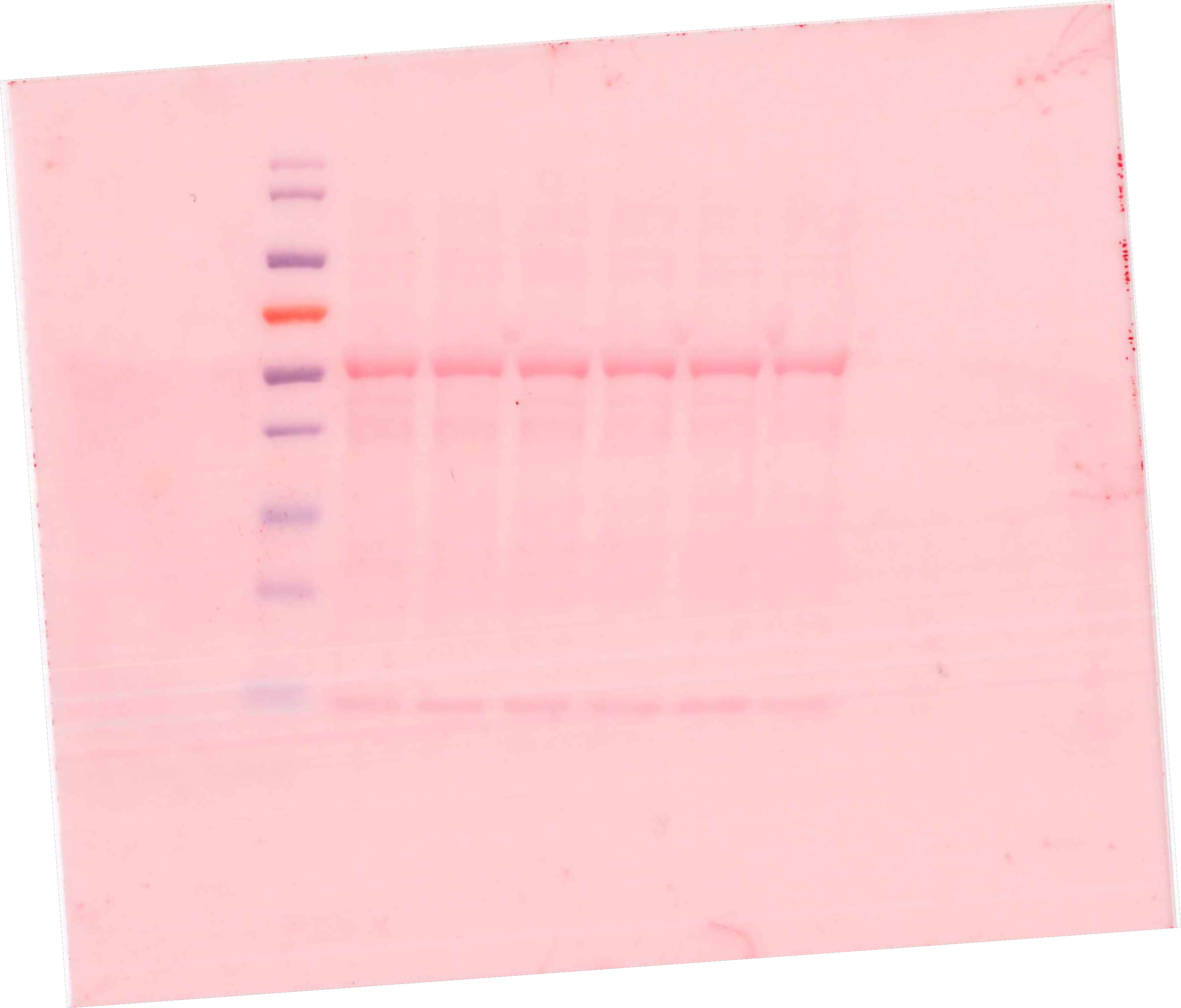

Supplement: Figure 2—figure supplement 1—source data 8. [file elife-81123-fig2-figsupp1-data8.tiff]

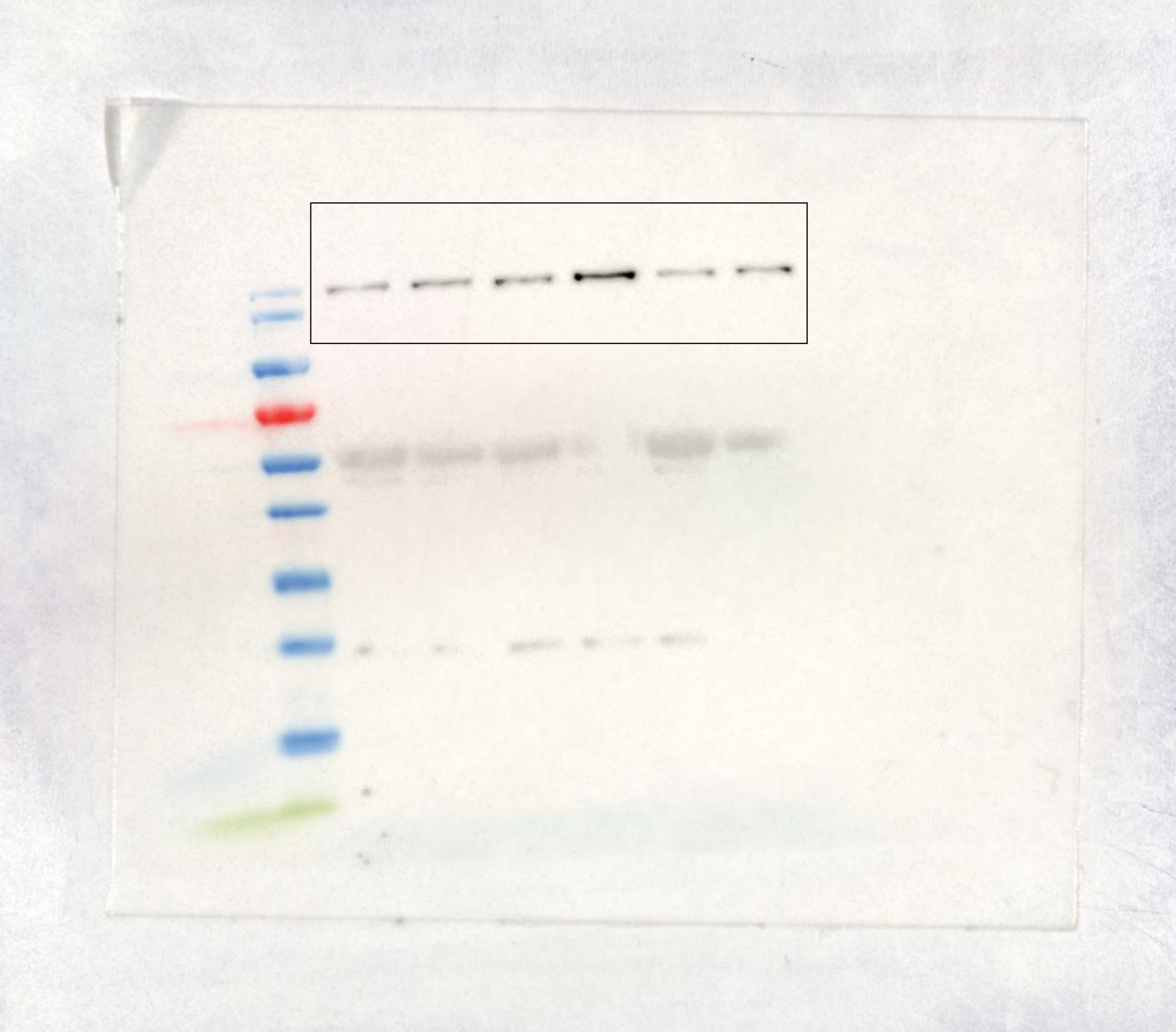

Supplement: Figure 2—figure supplement 5—source data 1. [file elife-81123-fig2-figsupp5-data1.tiff]

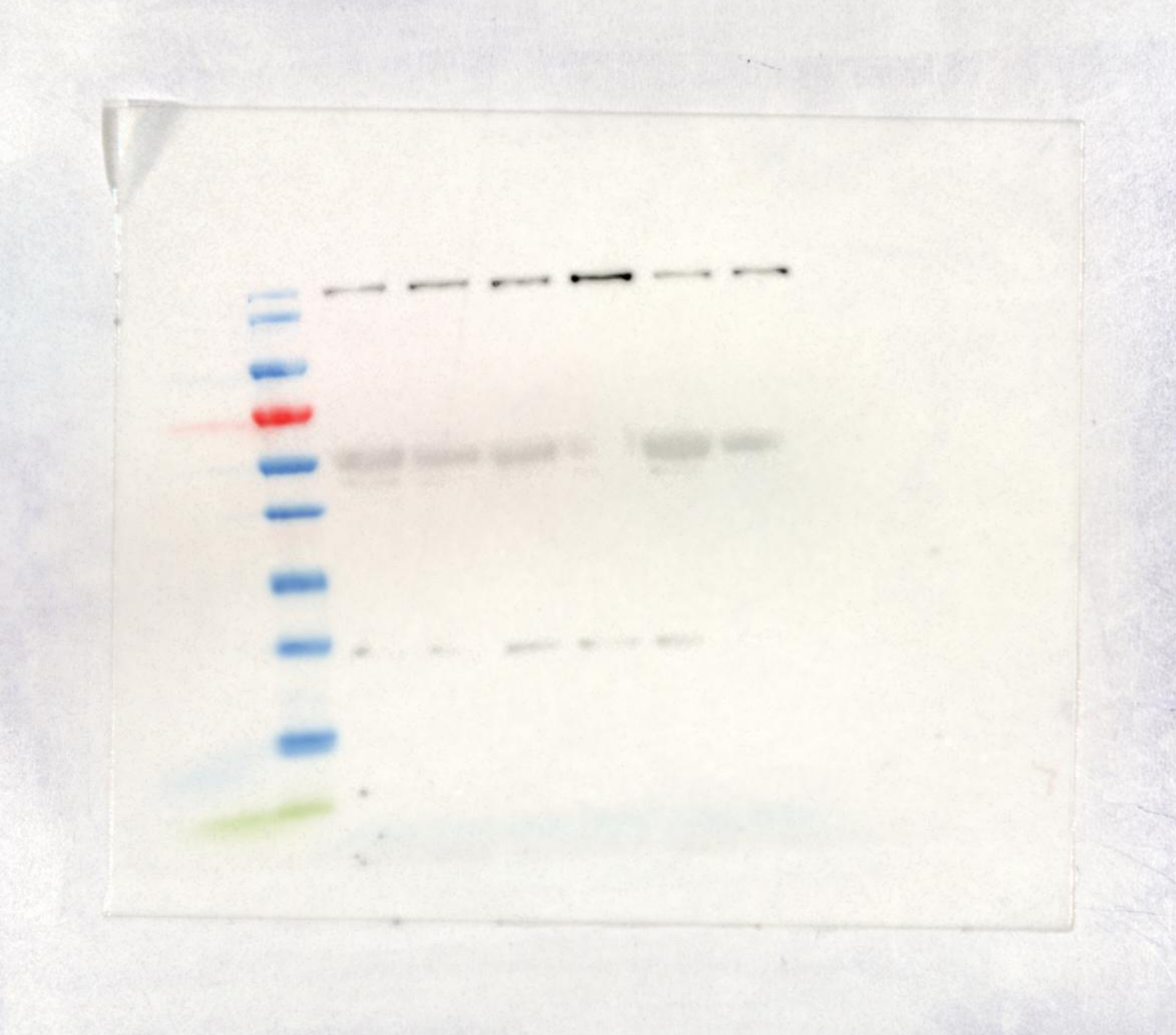

Supplement: Figure 2—figure supplement 5—source data 2. [file elife-81123-fig2-figsupp5-data2.tiff]

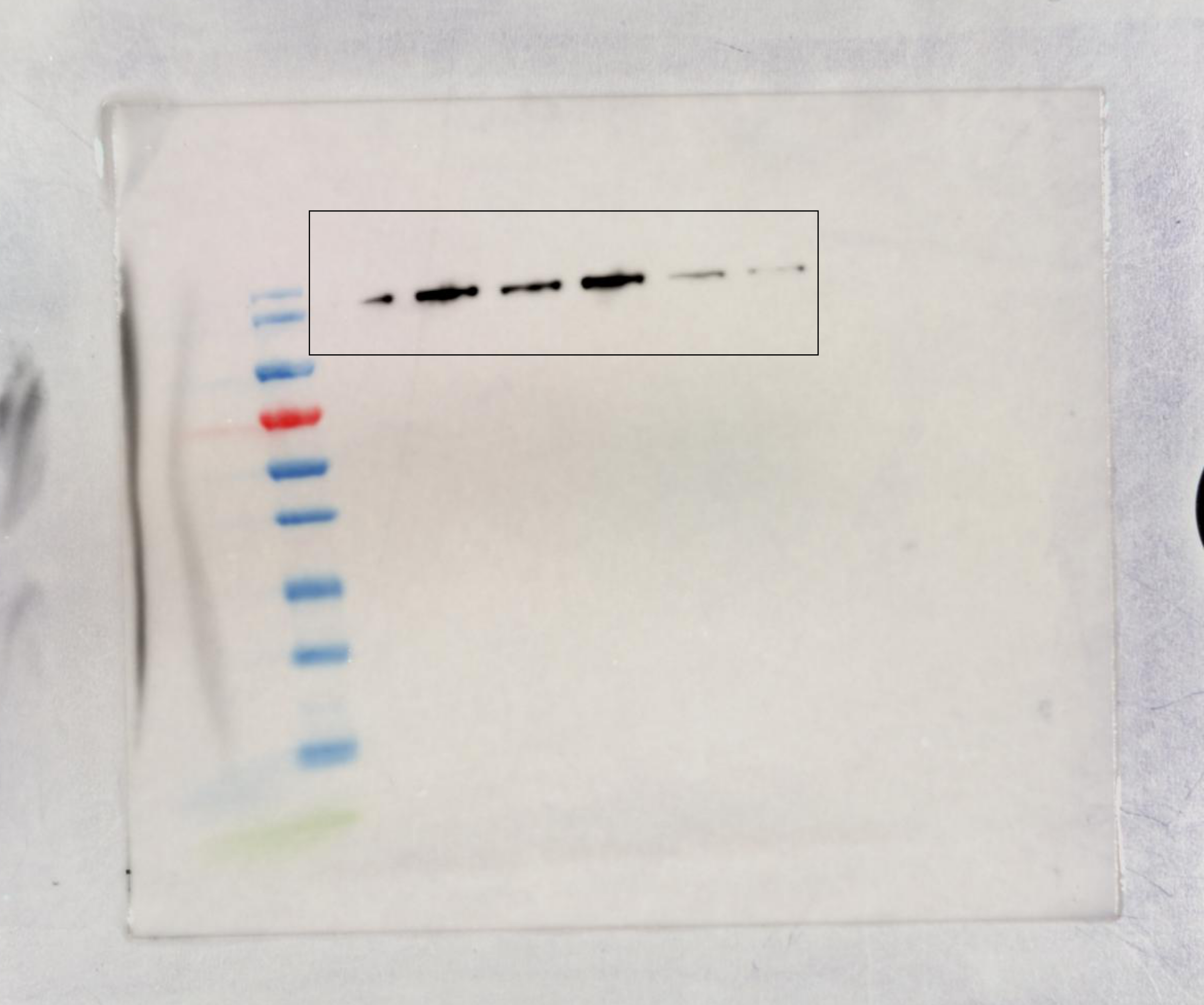

Supplement: Figure 2—figure supplement 5—source data 3. [file elife-81123-fig2-figsupp5-data3.tiff]

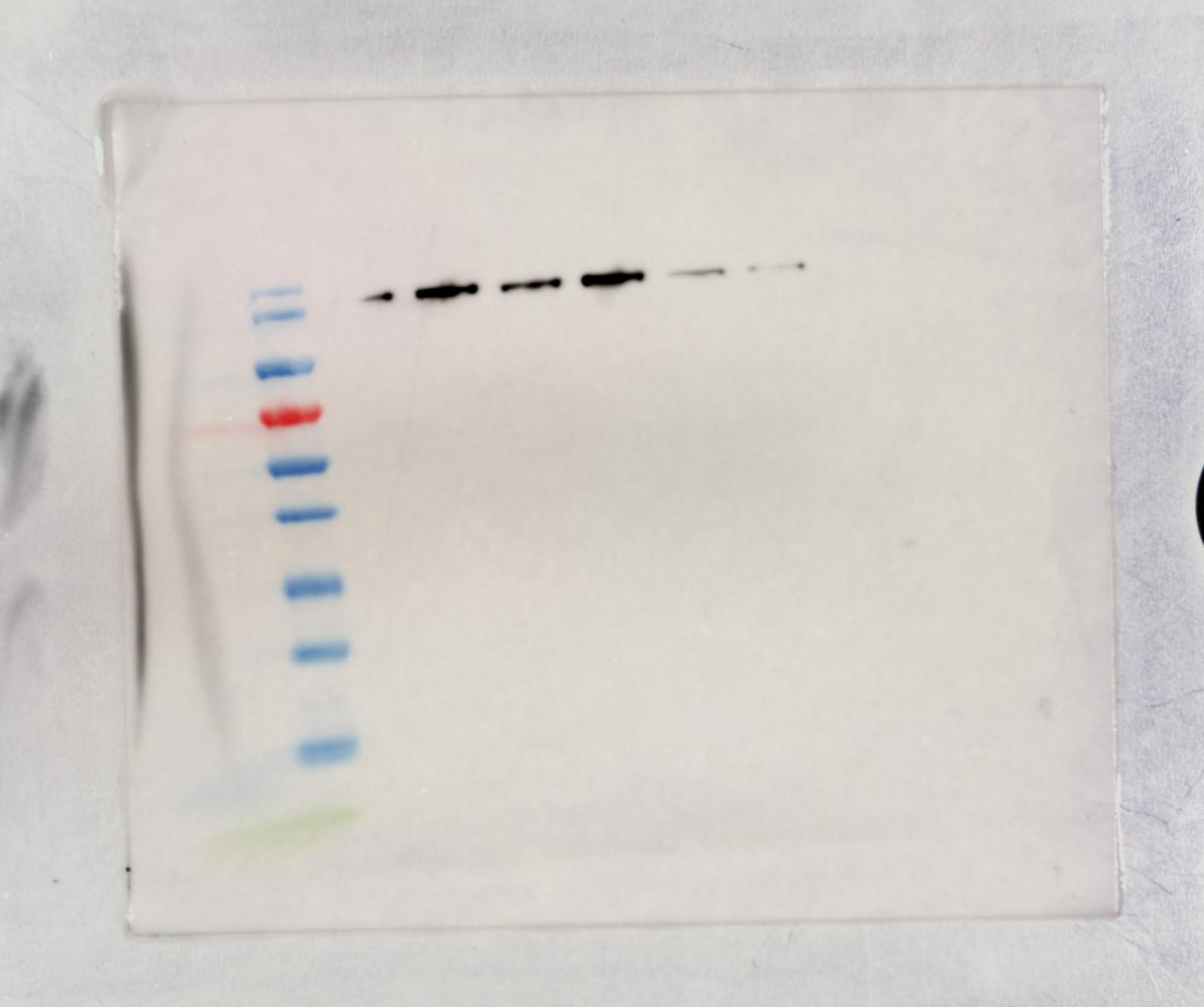

Supplement: Figure 2—figure supplement 5—source data 4. [file elife-81123-fig2-figsupp5-data4.tiff]

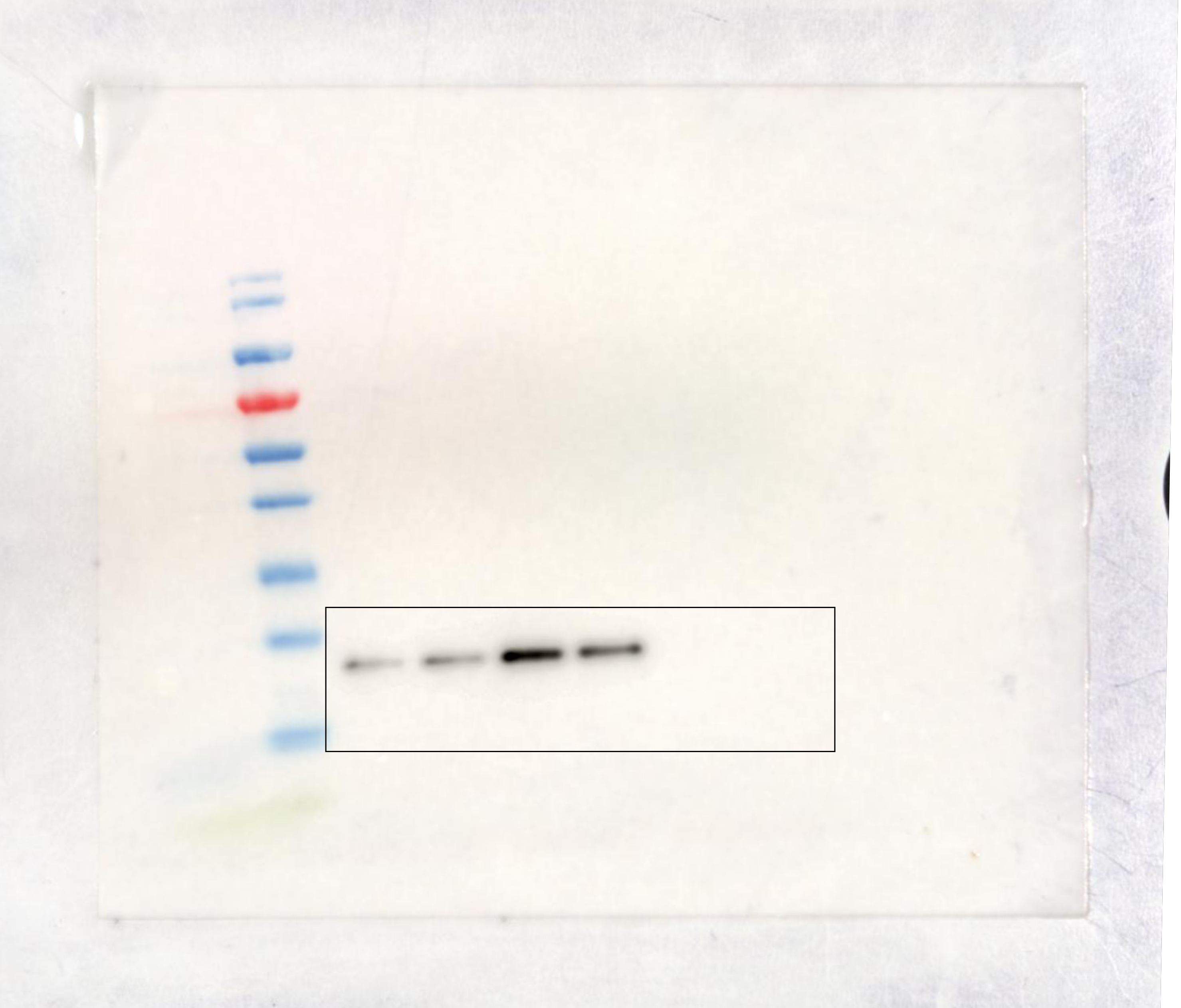

Supplement: Figure 2—figure supplement 5—source data 5. [file elife-81123-fig2-figsupp5-data5.tiff]

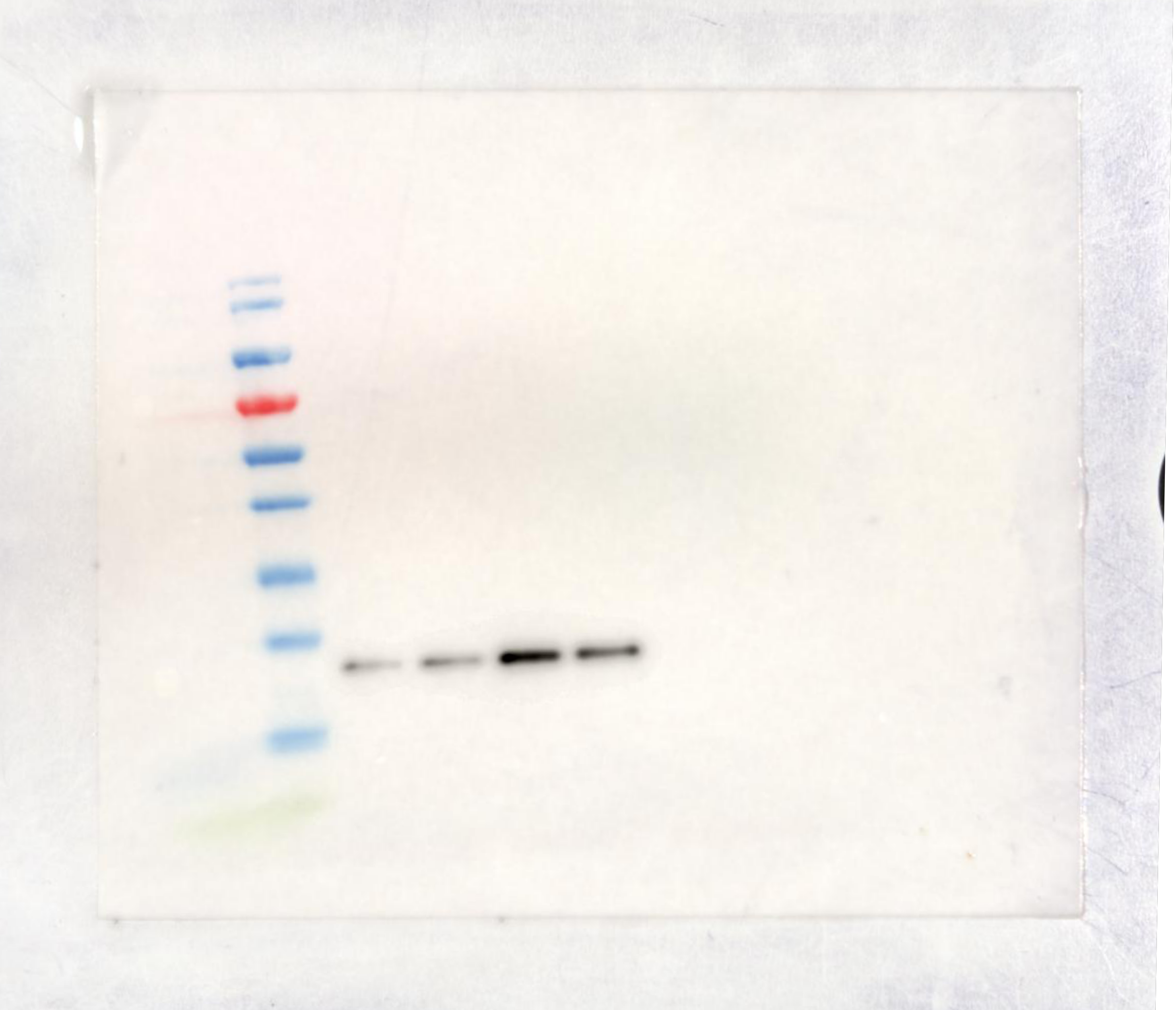

Supplement: Figure 2—figure supplement 5—source data 6. [file elife-81123-fig2-figsupp5-data6.tiff]

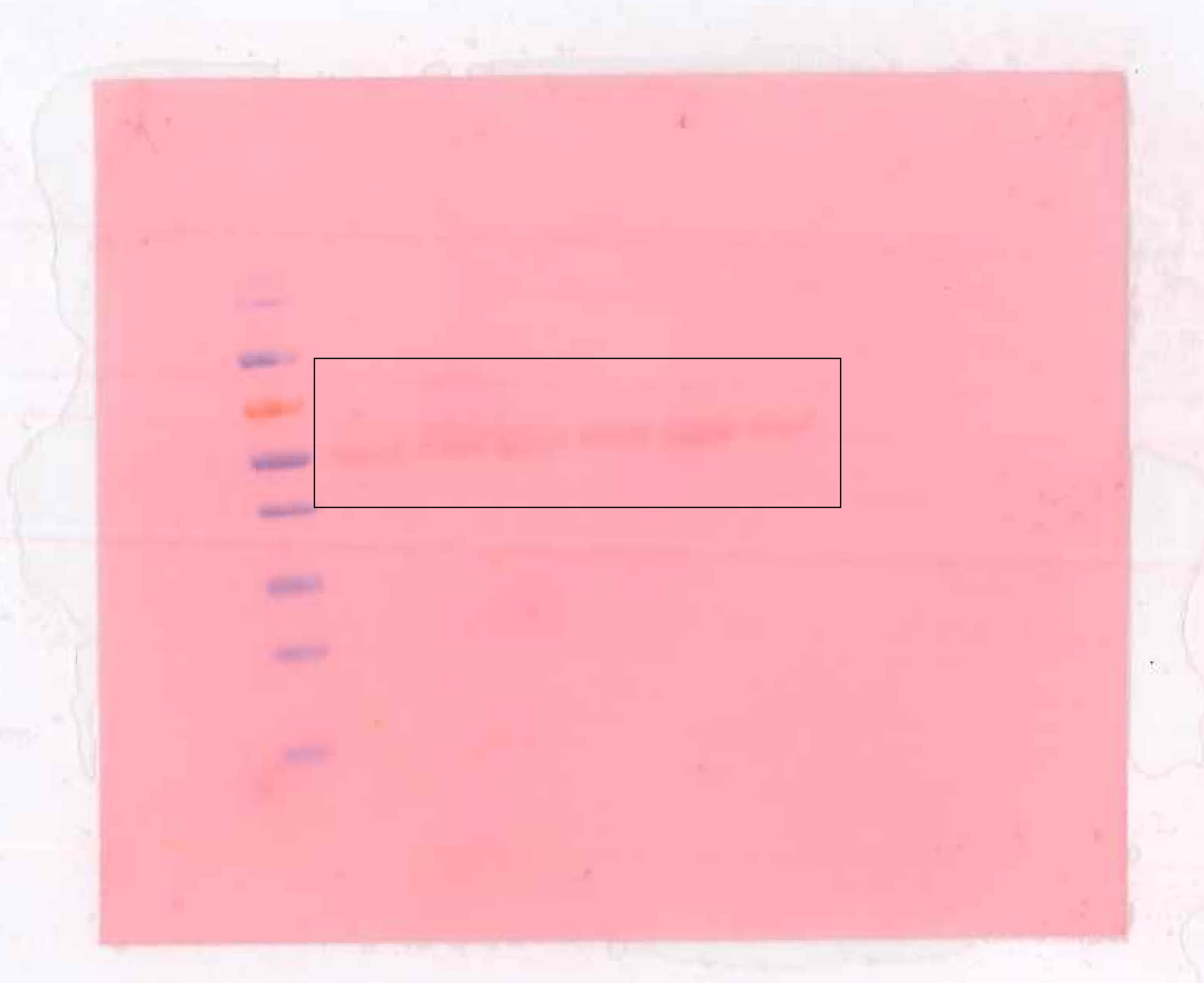

Supplement: Figure 2—figure supplement 5—source data 7. — Ponceau stain, with relevant bands labeled. [file elife-81123-fig2-figsupp5-data7.tiff]

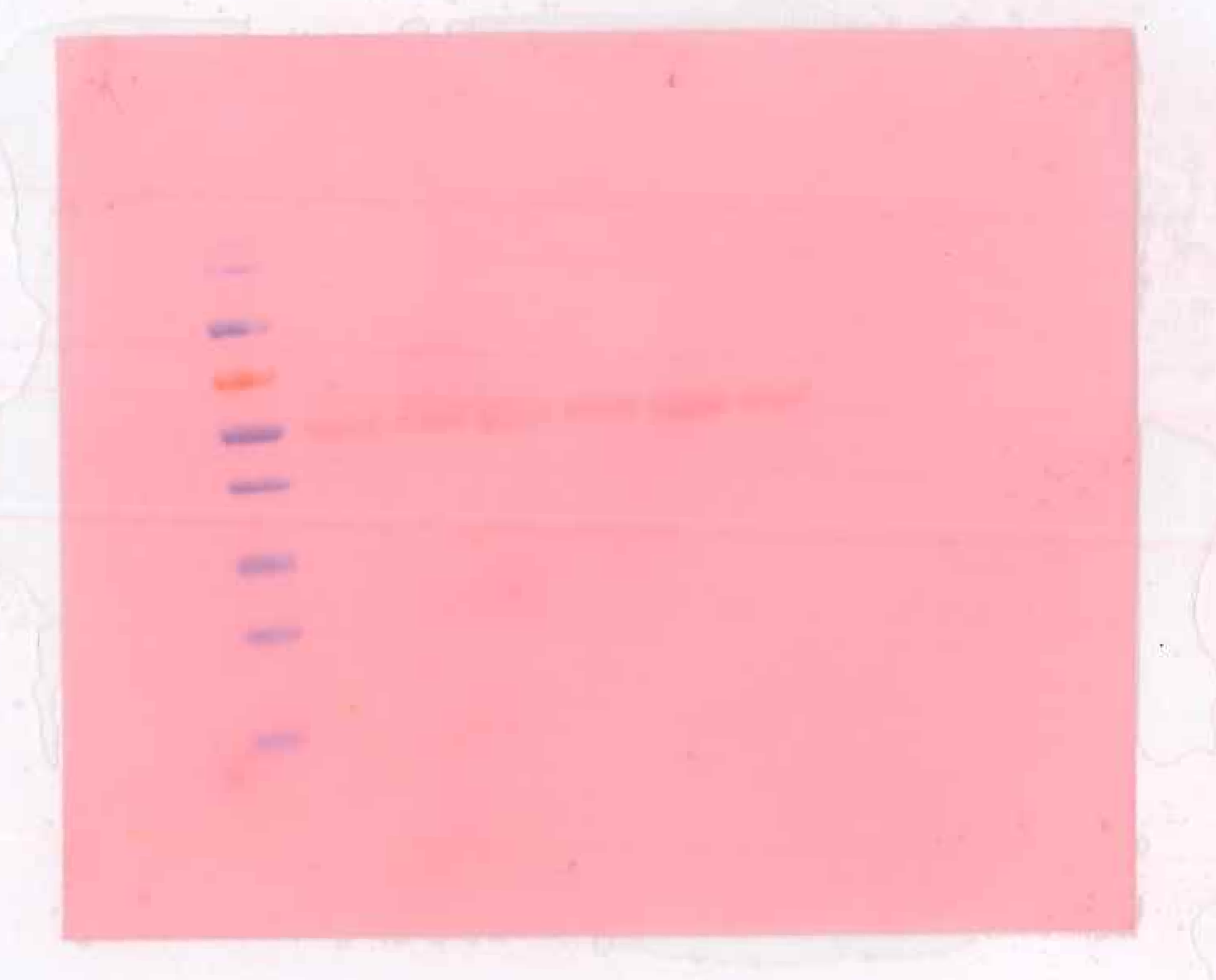

Supplement: Figure 2—figure supplement 5—source data 8. [file elife-81123-fig2-figsupp5-data8.tiff]

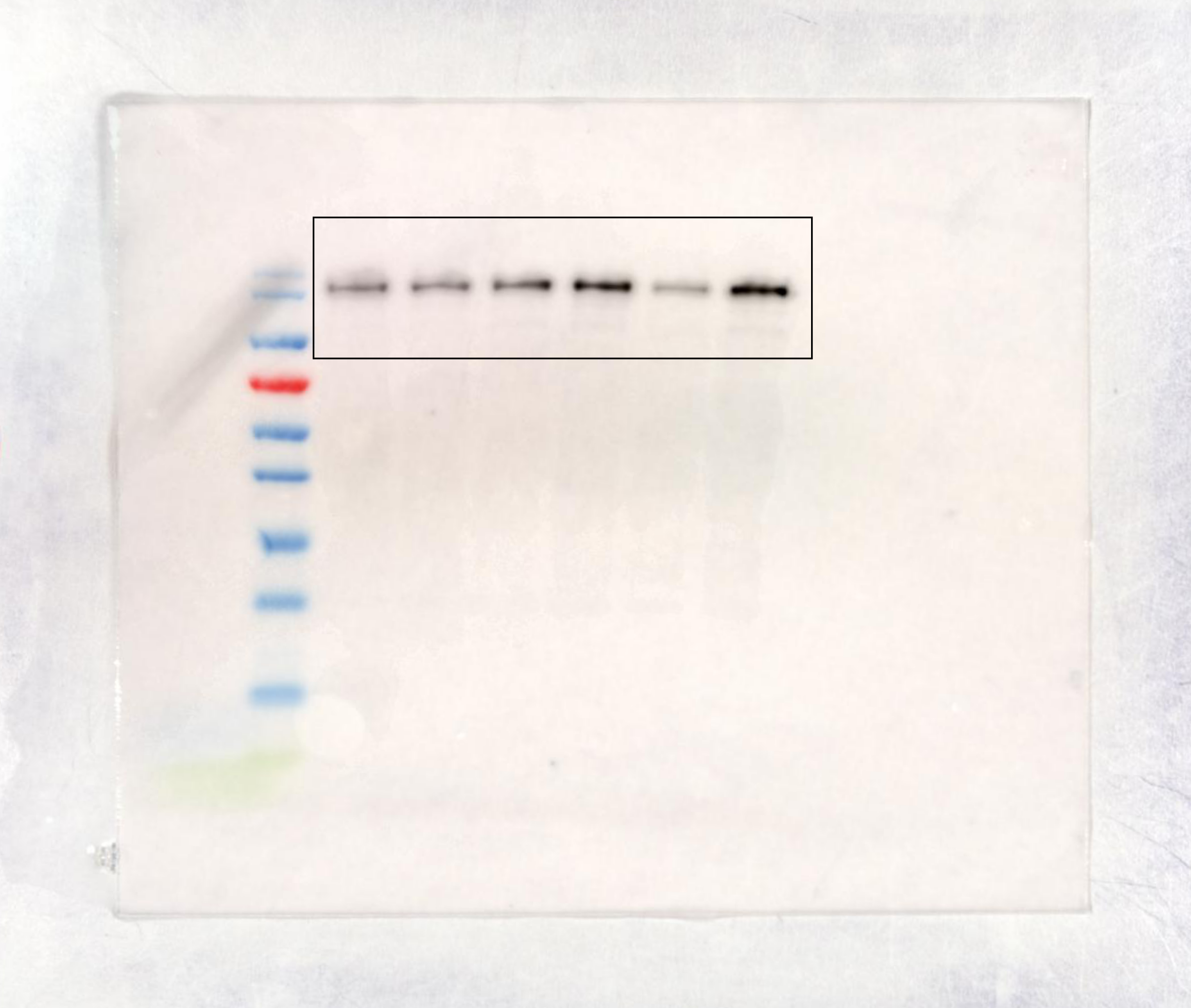

Supplement: Figure 2—figure supplement 5—source data 9. [file elife-81123-fig2-figsupp5-data9.tiff]

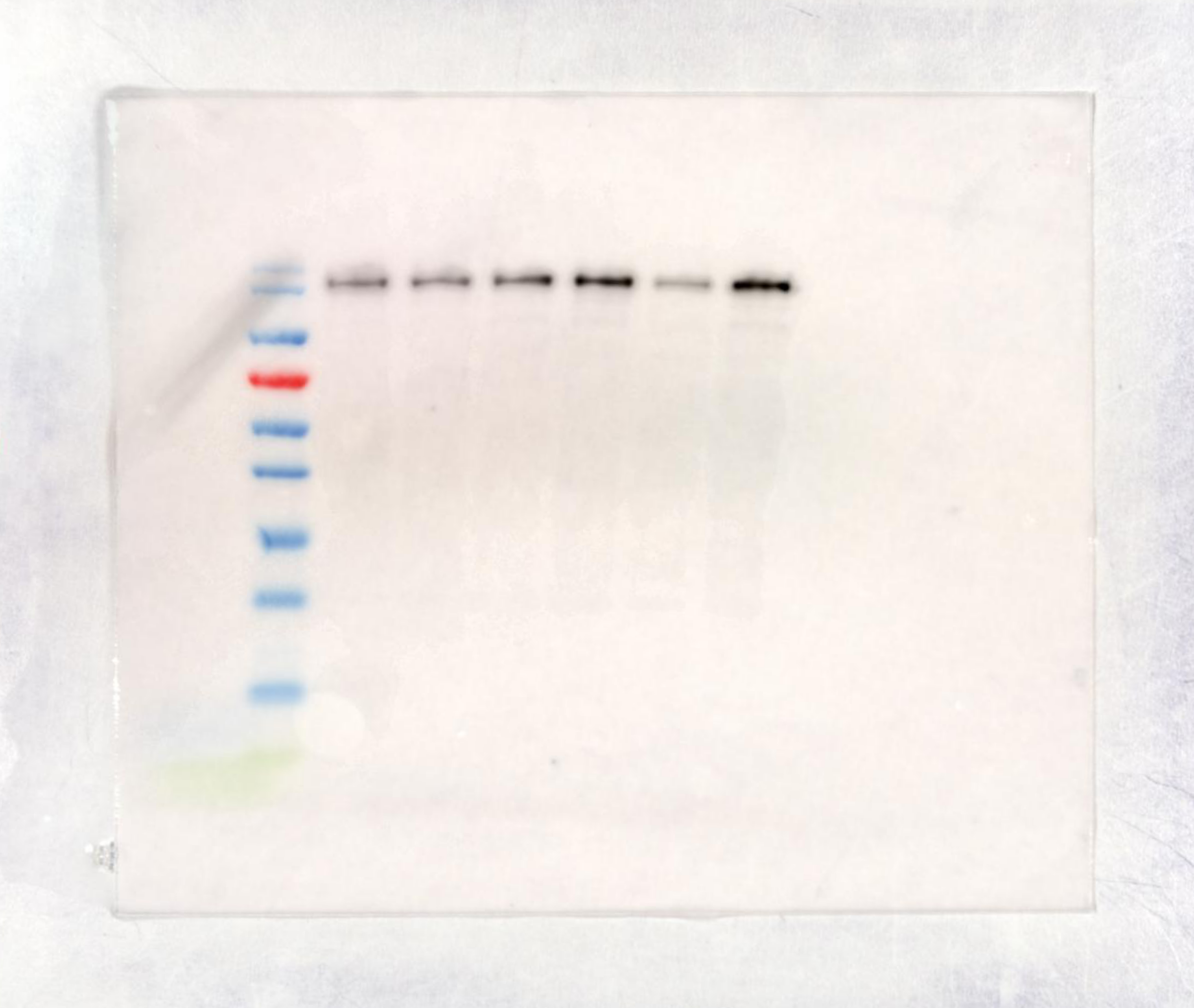

Supplement: Figure 2—figure supplement 5—source data 10. [file elife-81123-fig2-figsupp5-data10.tiff]

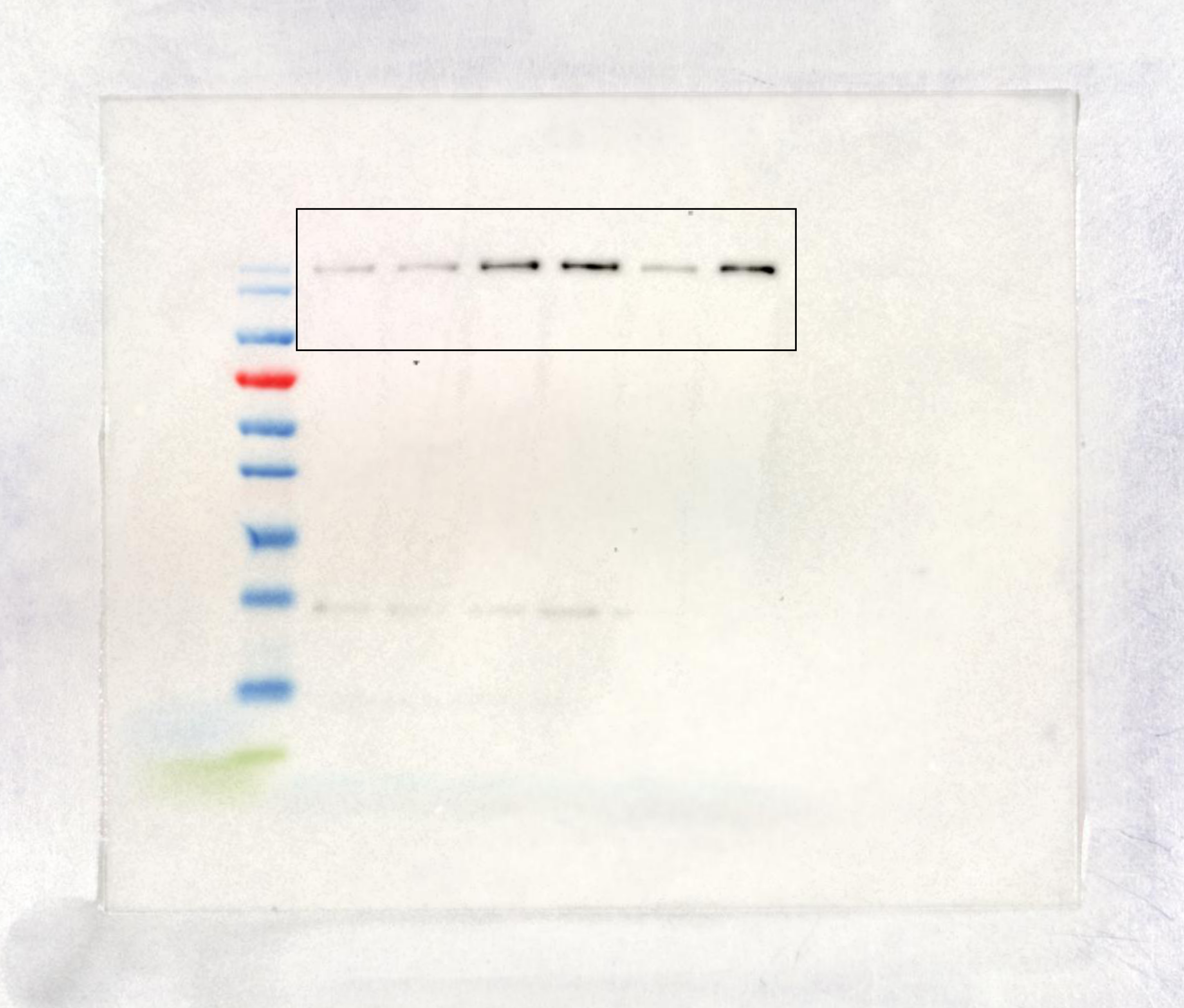

Supplement: Figure 2—figure supplement 5—source data 11. [file elife-81123-fig2-figsupp5-data11.tiff]

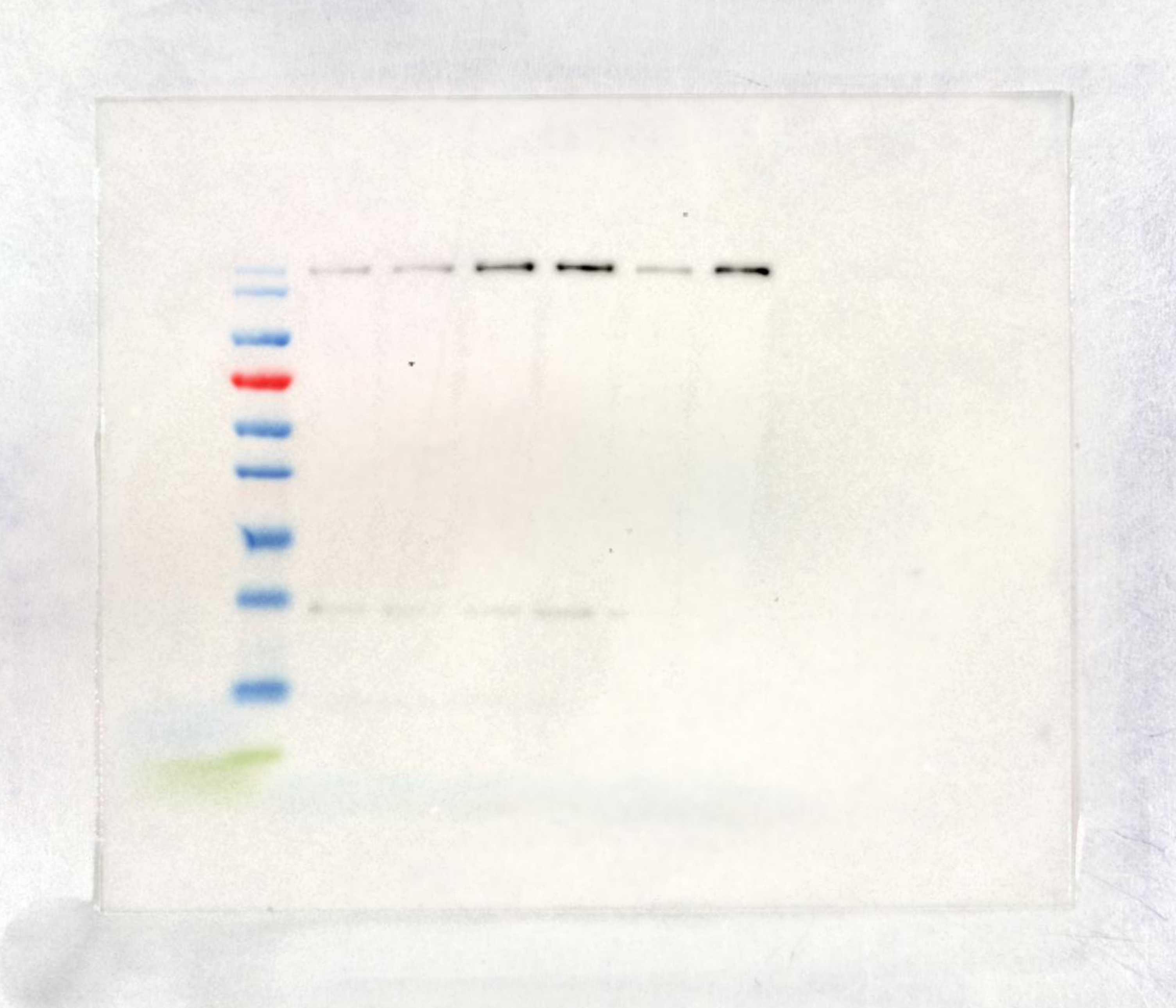

Supplement: Figure 2—figure supplement 5—source data 12. [file elife-81123-fig2-figsupp5-data12.tiff]

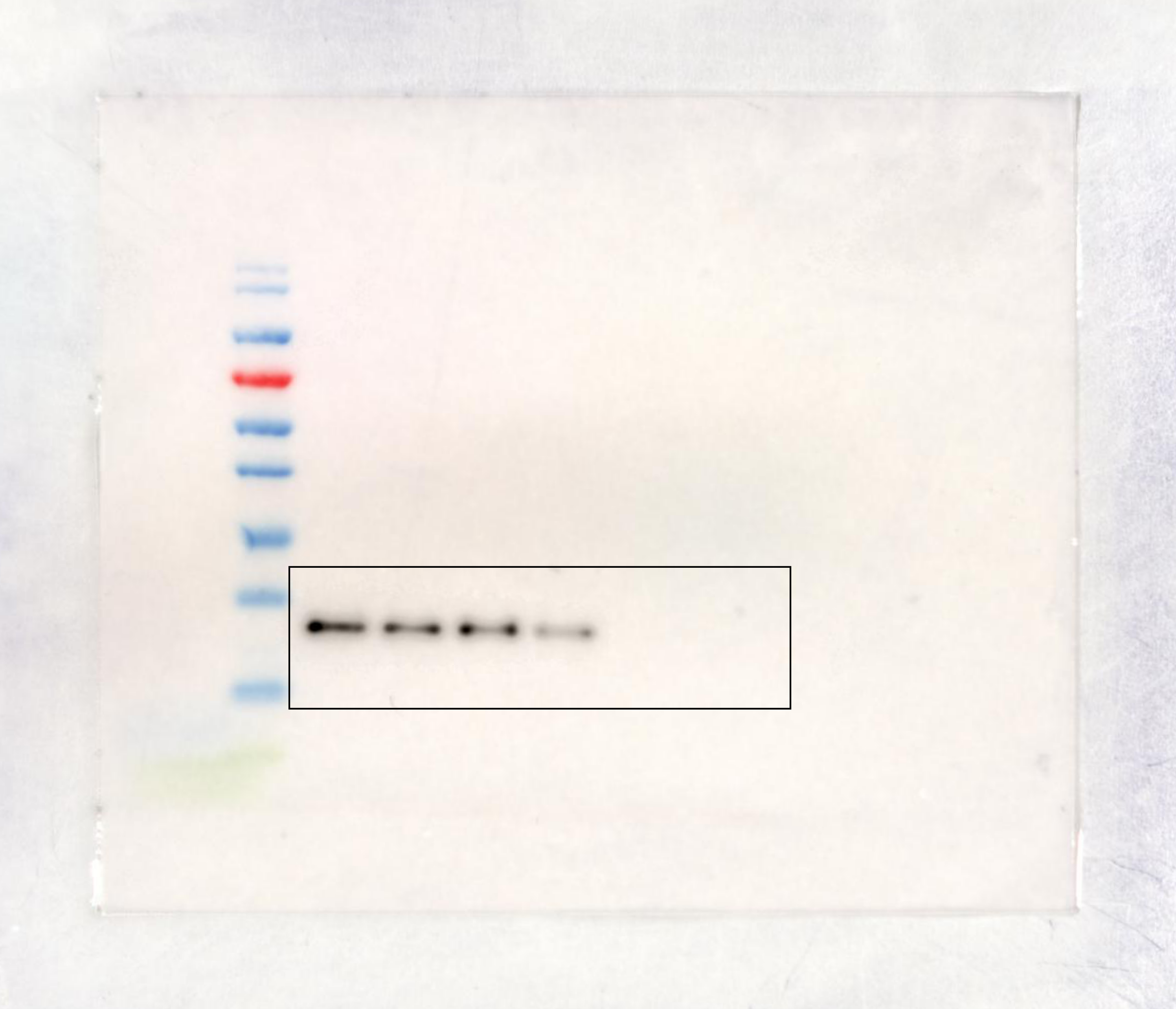

Supplement: Figure 2—figure supplement 5—source data 13. [file elife-81123-fig2-figsupp5-data13.tiff]

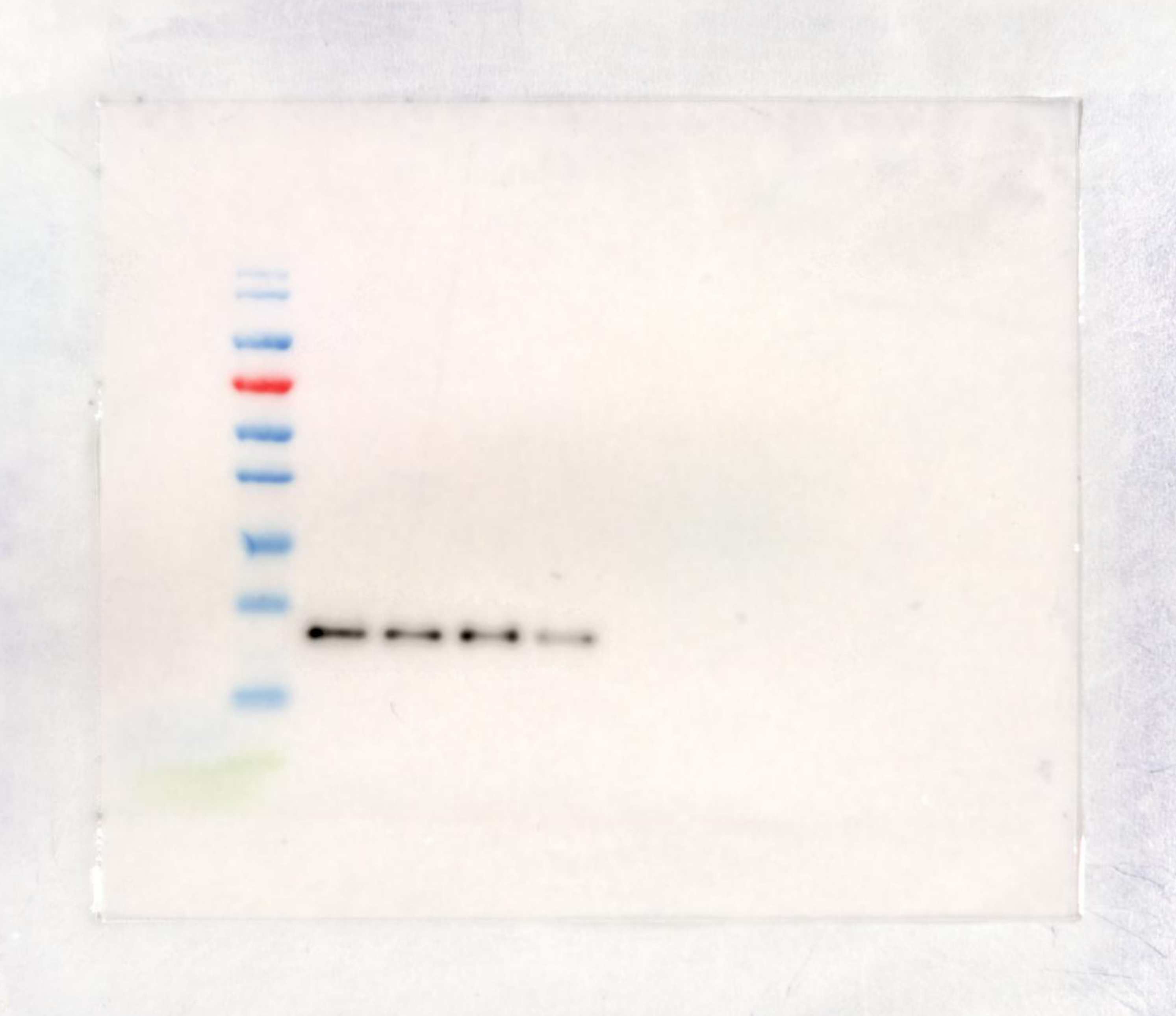

Supplement: Figure 2—figure supplement 5—source data 14. [file elife-81123-fig2-figsupp5-data14.tiff]

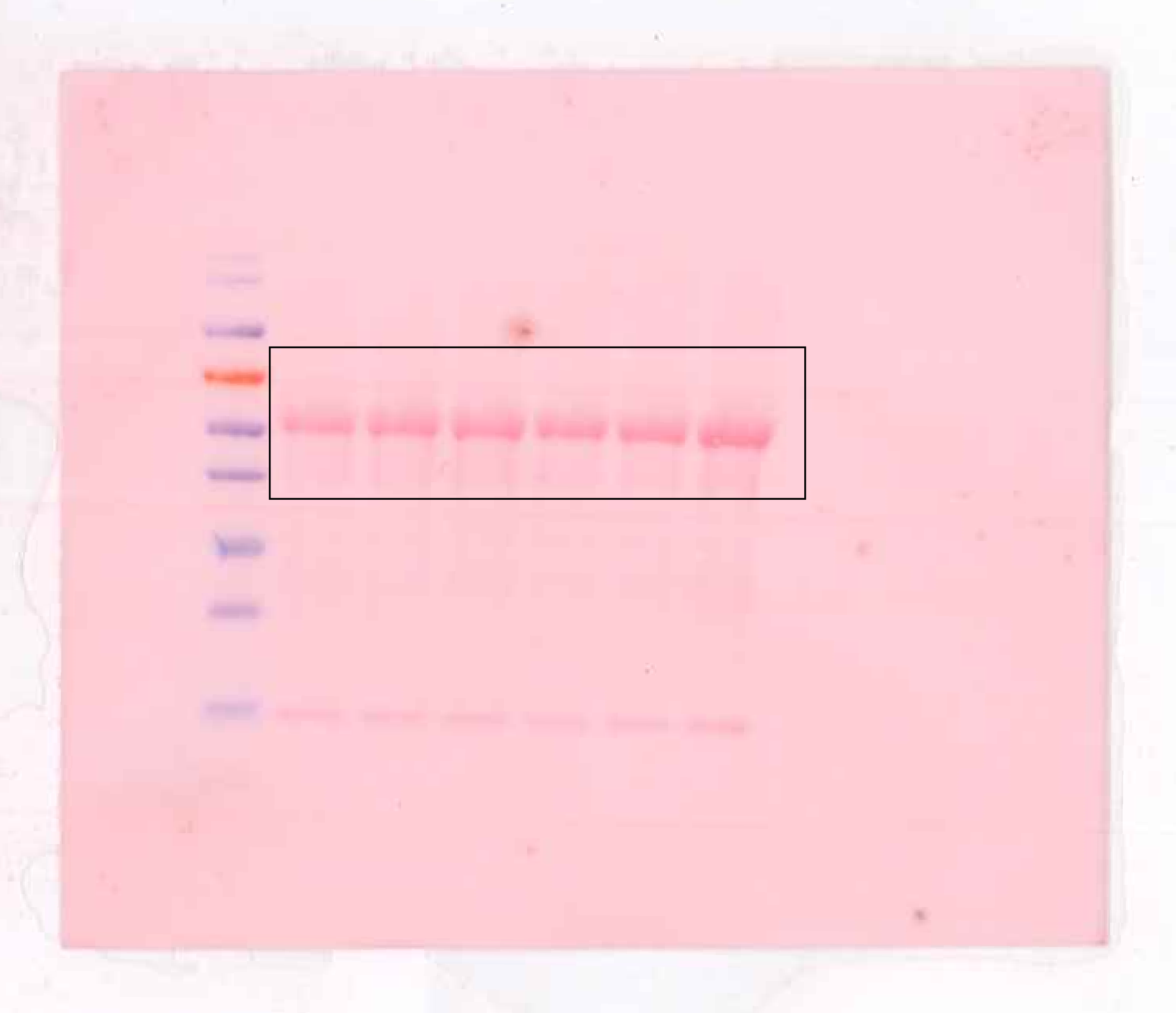

Supplement: Figure 2—figure supplement 5—source data 15. [file elife-81123-fig2-figsupp5-data15.tiff]

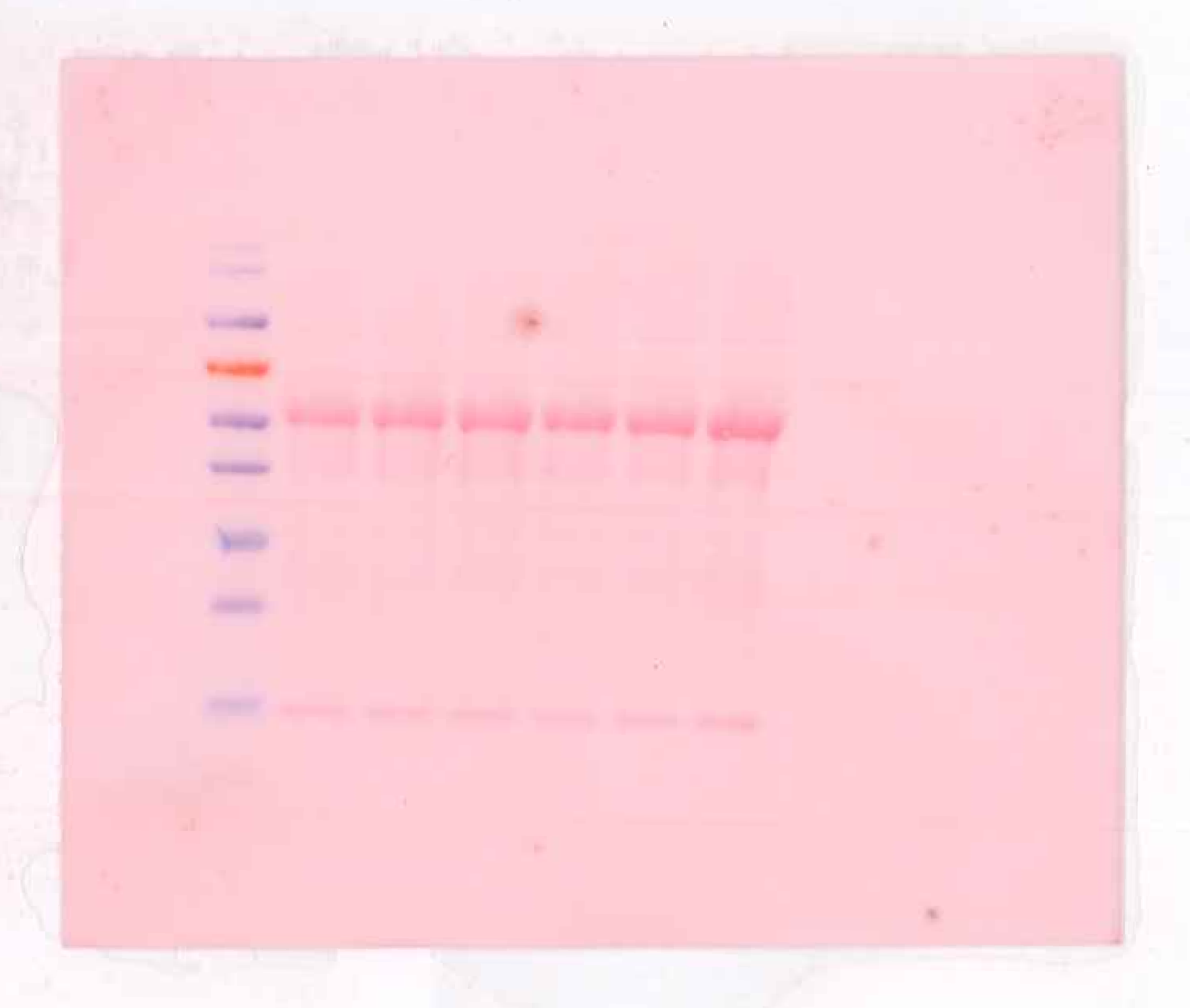

Supplement: Figure 2—figure supplement 5—source data 16. [file elife-81123-fig2-figsupp5-data16.tiff]

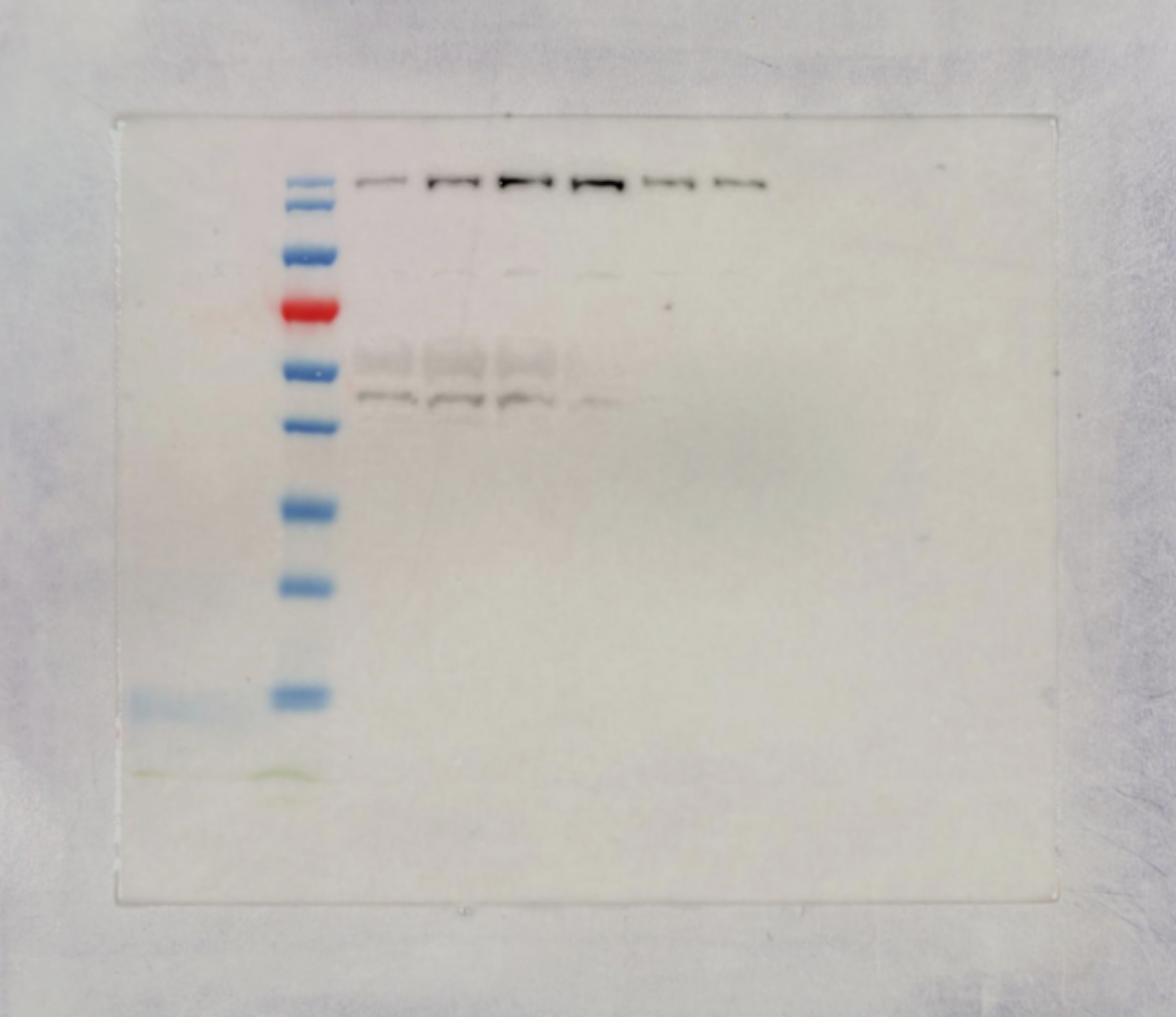

Supplement: Figure 2—figure supplement 8—source data 2. [file elife-81123-fig2-figsupp8-data2.tiff]

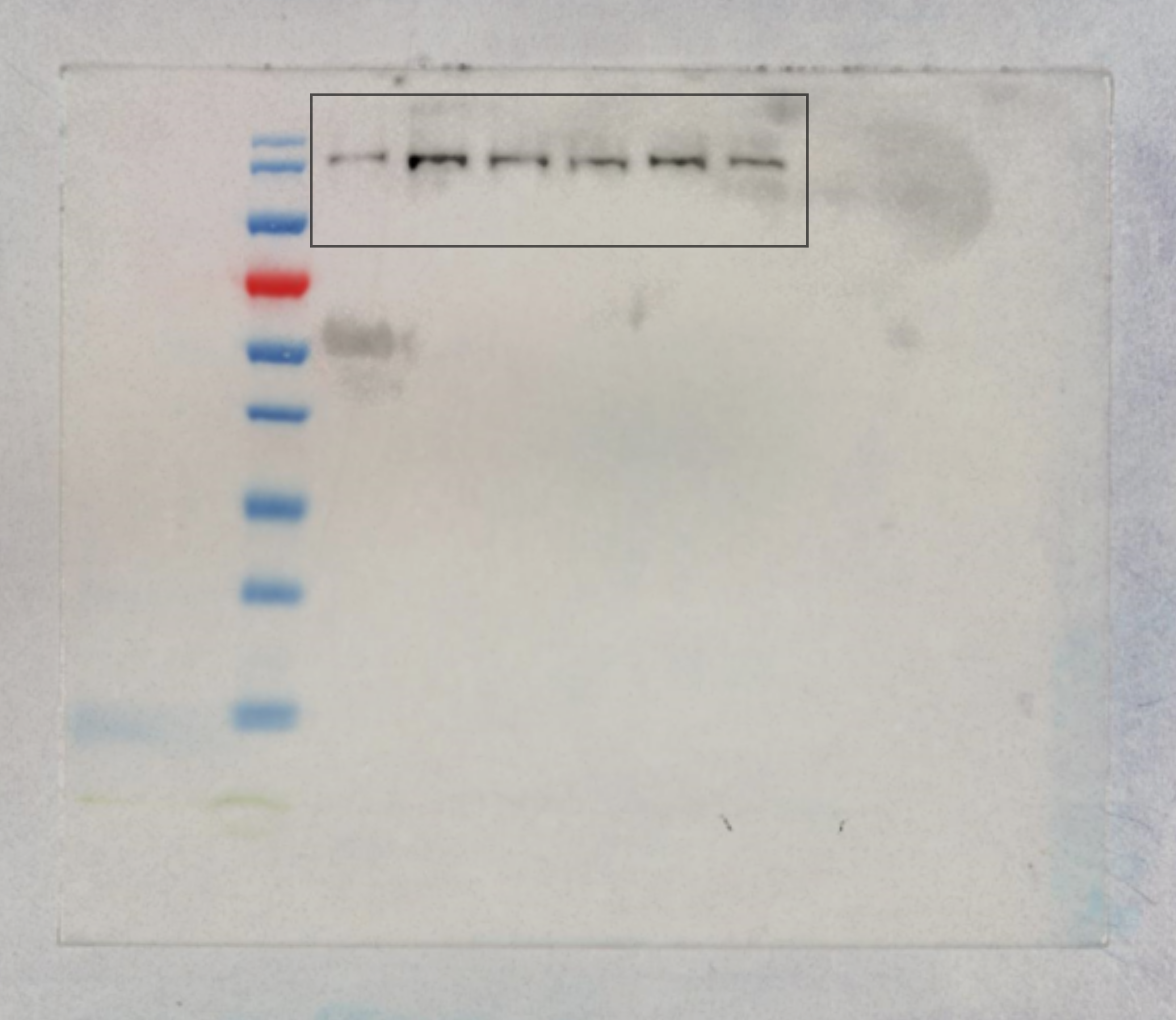

Supplement: Figure 2—figure supplement 8—source data 3. [file elife-81123-fig2-figsupp8-data3.tiff]

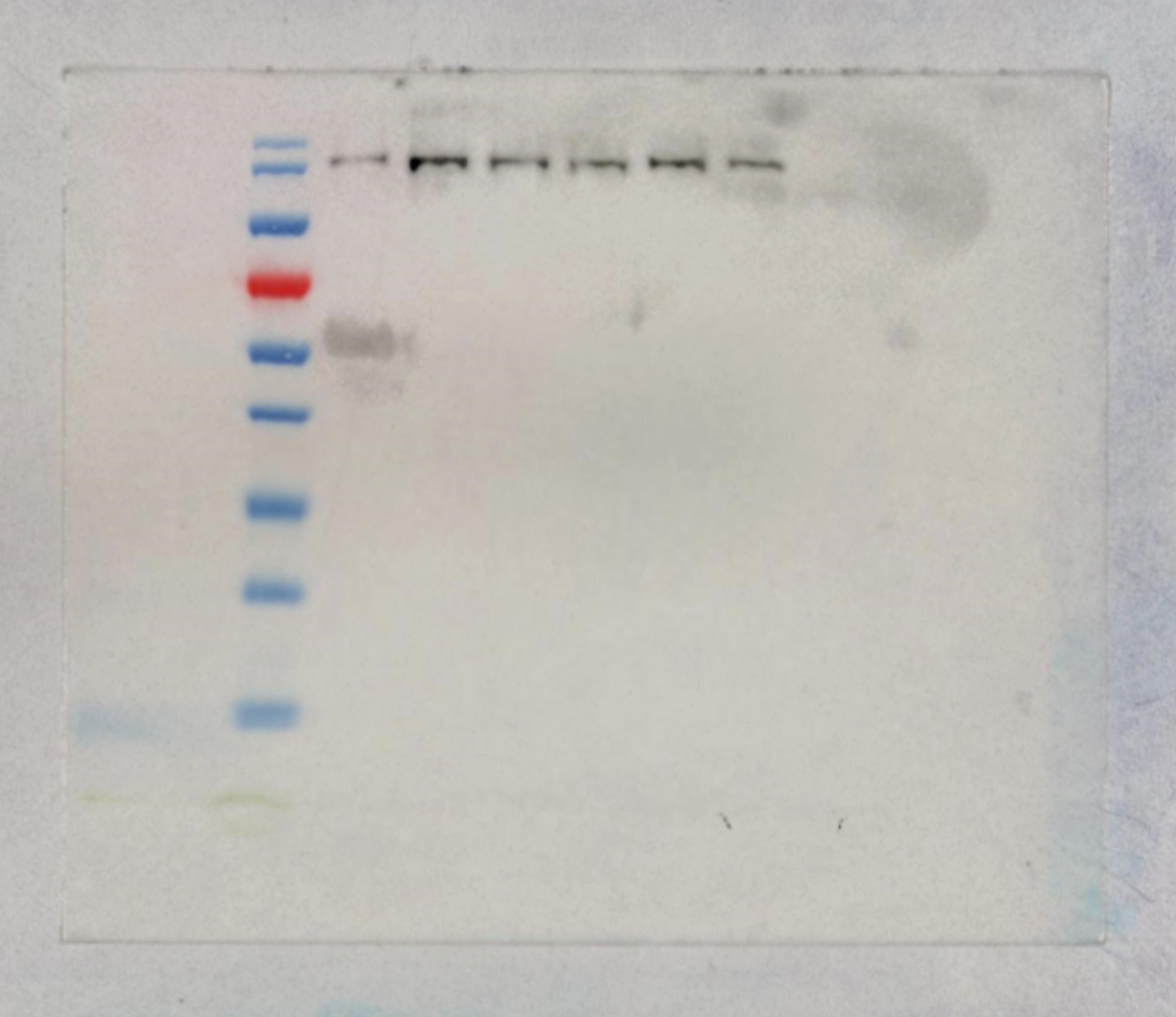

Supplement: Figure 2—figure supplement 8—source data 4. [file elife-81123-fig2-figsupp8-data4.tiff]

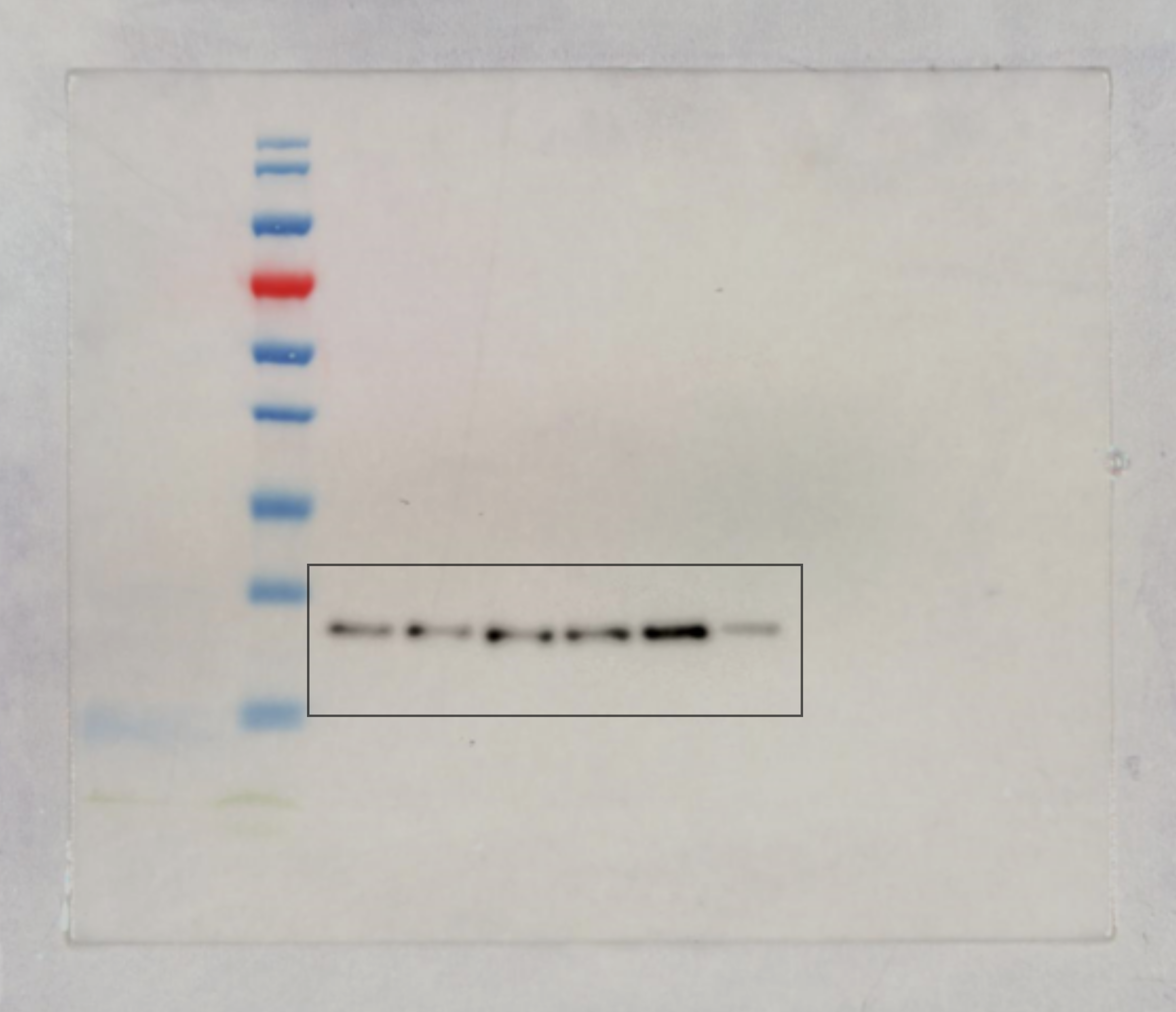

Supplement: Figure 2—figure supplement 8—source data 5. [file elife-81123-fig2-figsupp8-data5.tiff]

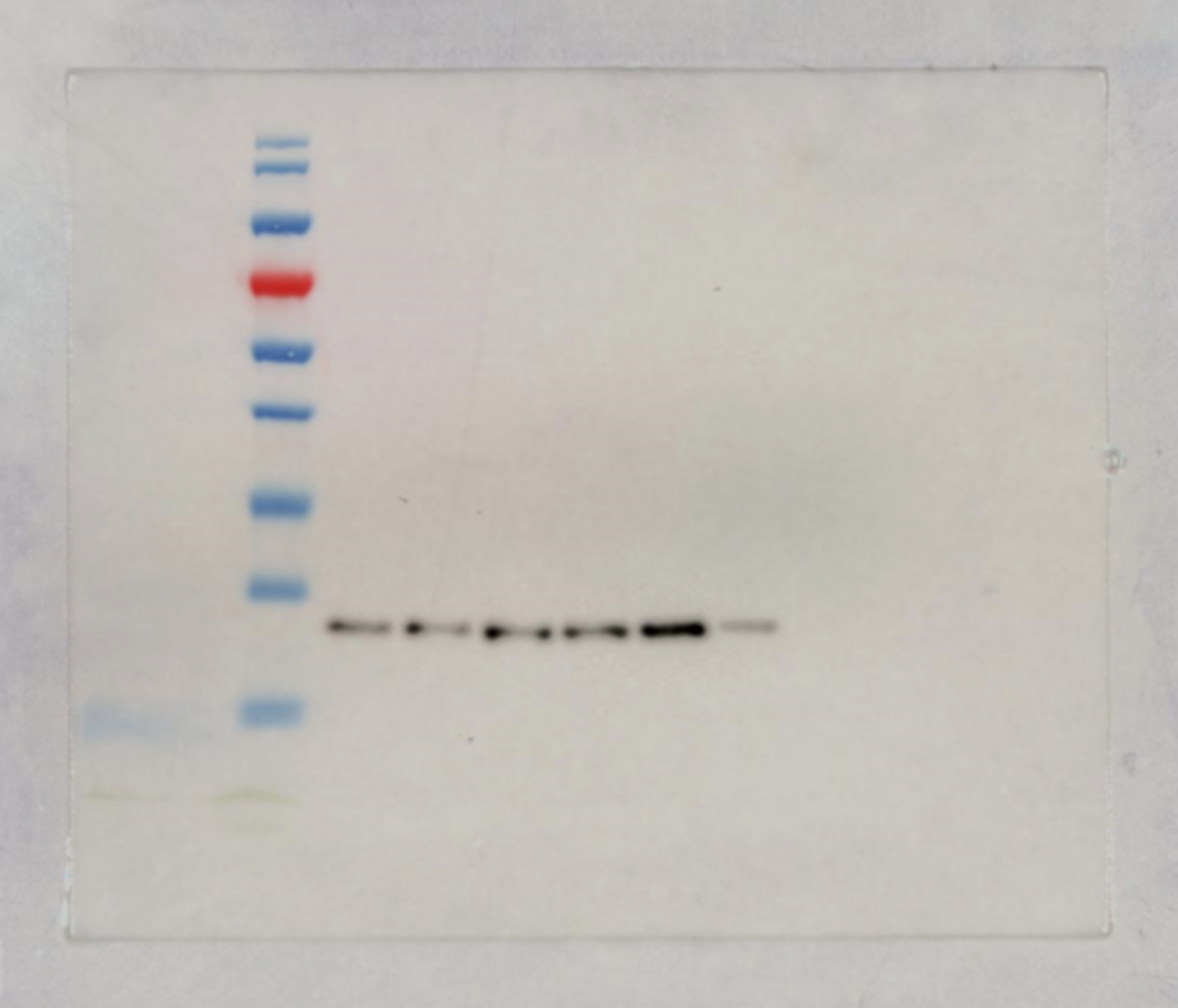

Supplement: Figure 2—figure supplement 8—source data 6. [file elife-81123-fig2-figsupp8-data6.tiff]

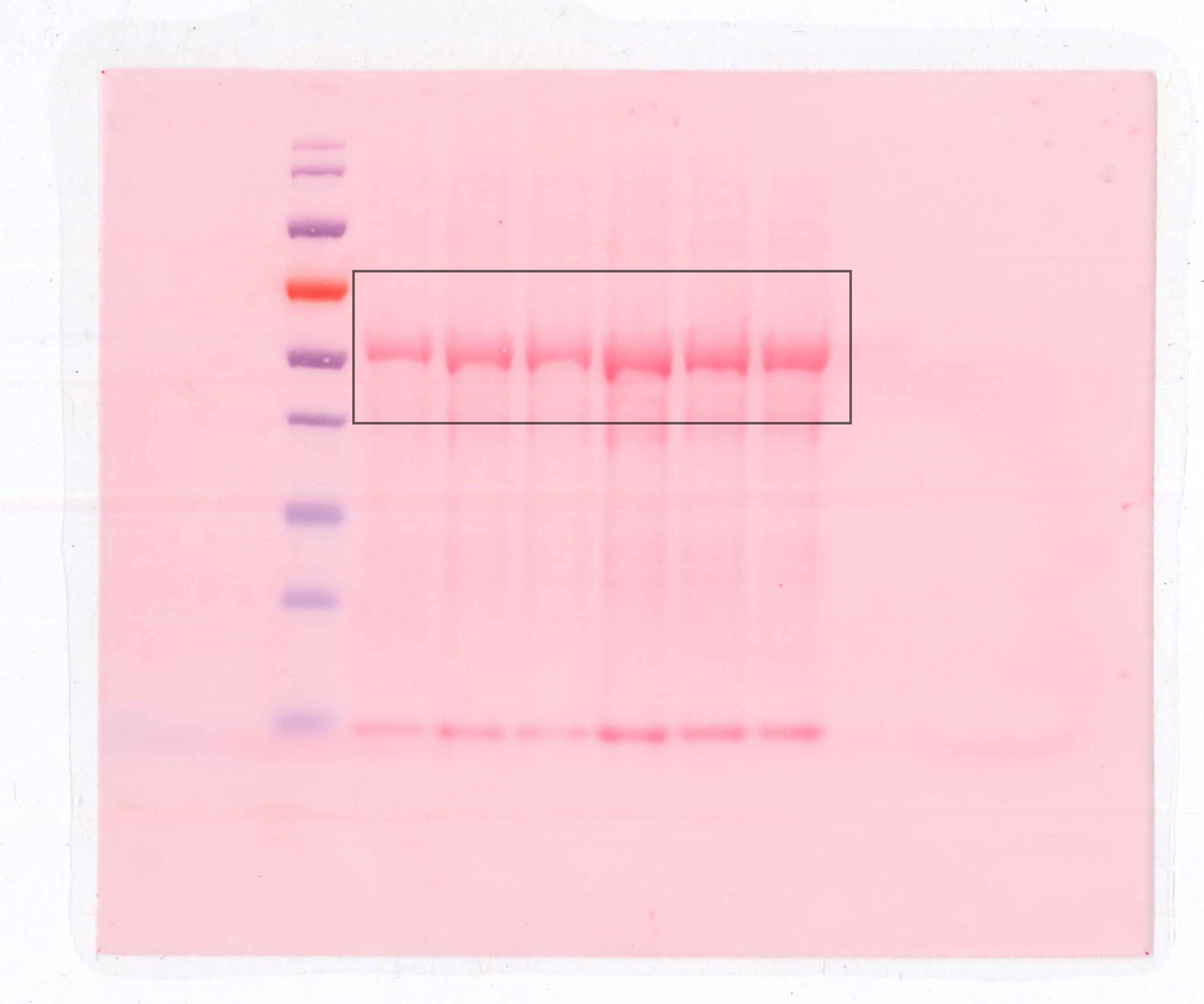

Supplement: Figure 2—figure supplement 8—source data 7. [file elife-81123-fig2-figsupp8-data7.tiff]

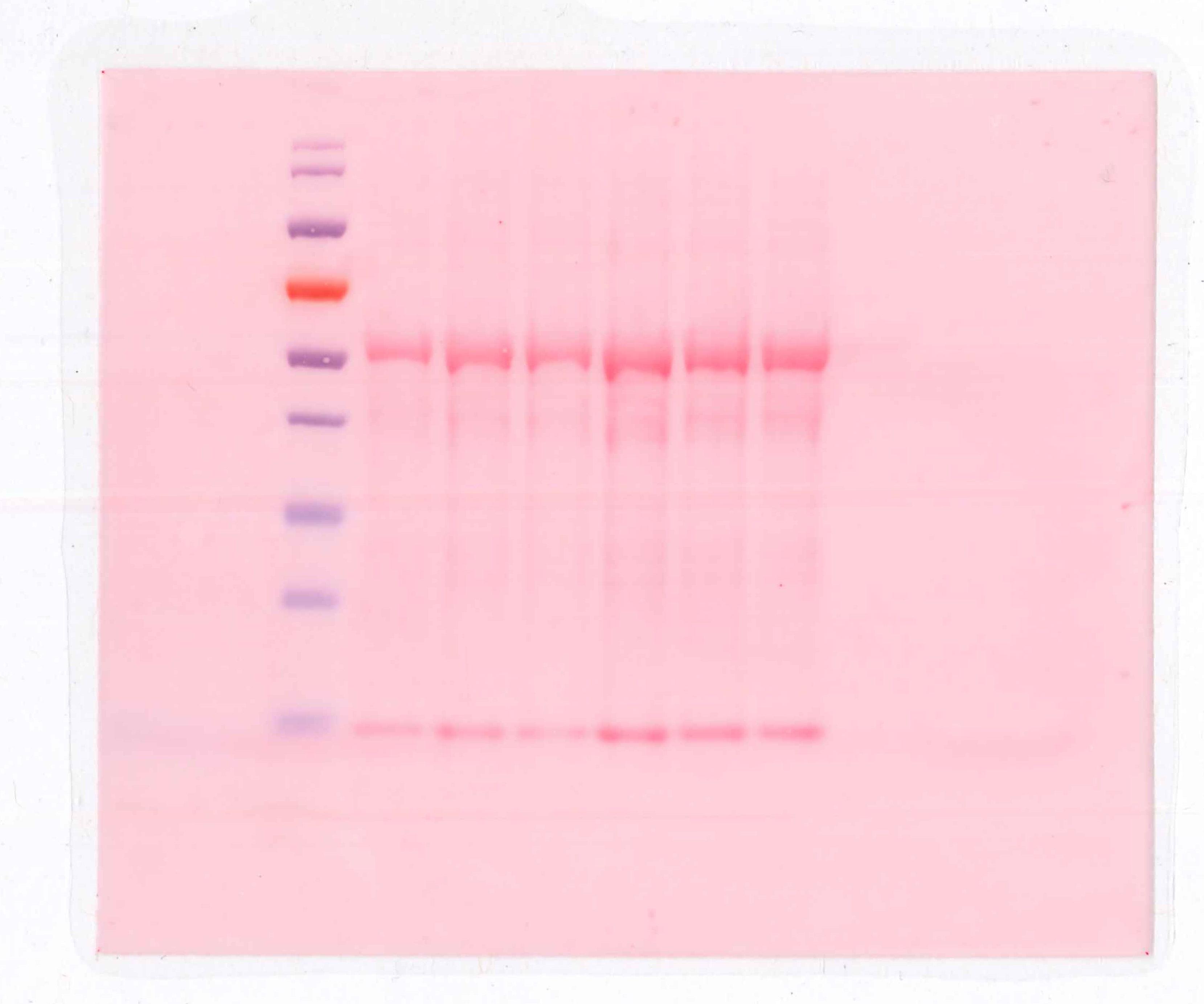

Supplement: Figure 2—figure supplement 8—source data 8. [file elife-81123-fig2-figsupp8-data8.tiff]

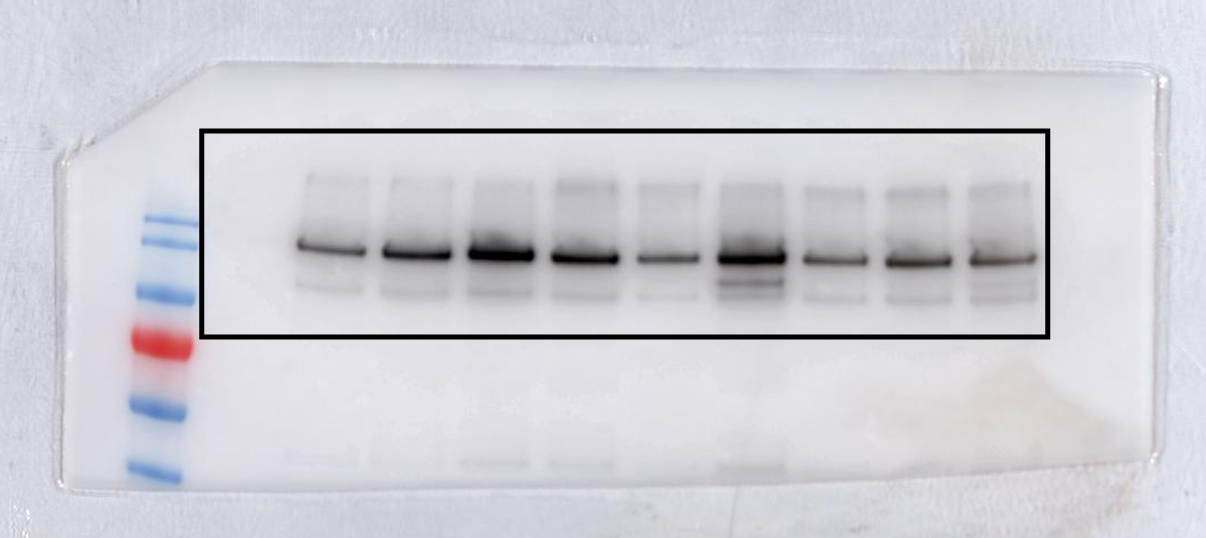

Supplement: Figure 3—source data 1. [file elife-81123-fig3-data1.tiff]

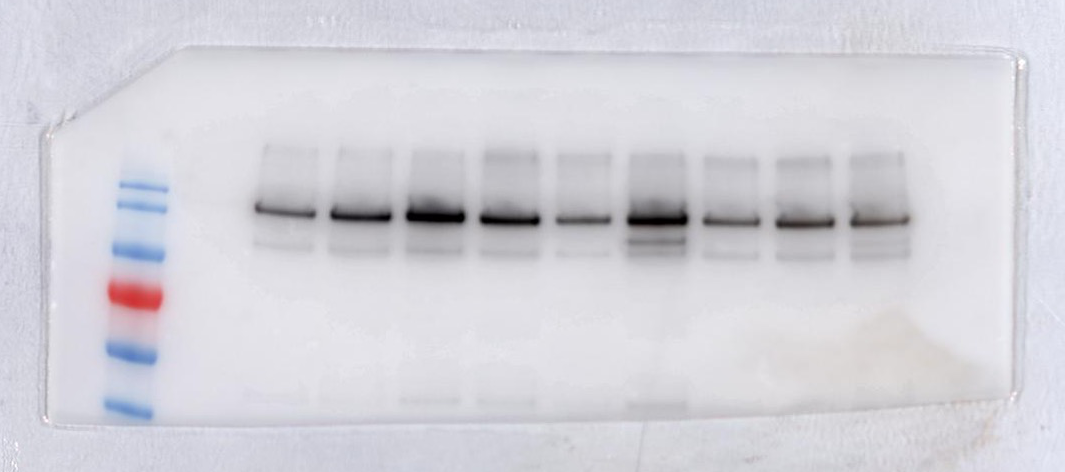

Supplement: Figure 3—source data 2. [file elife-81123-fig3-data2.tiff]

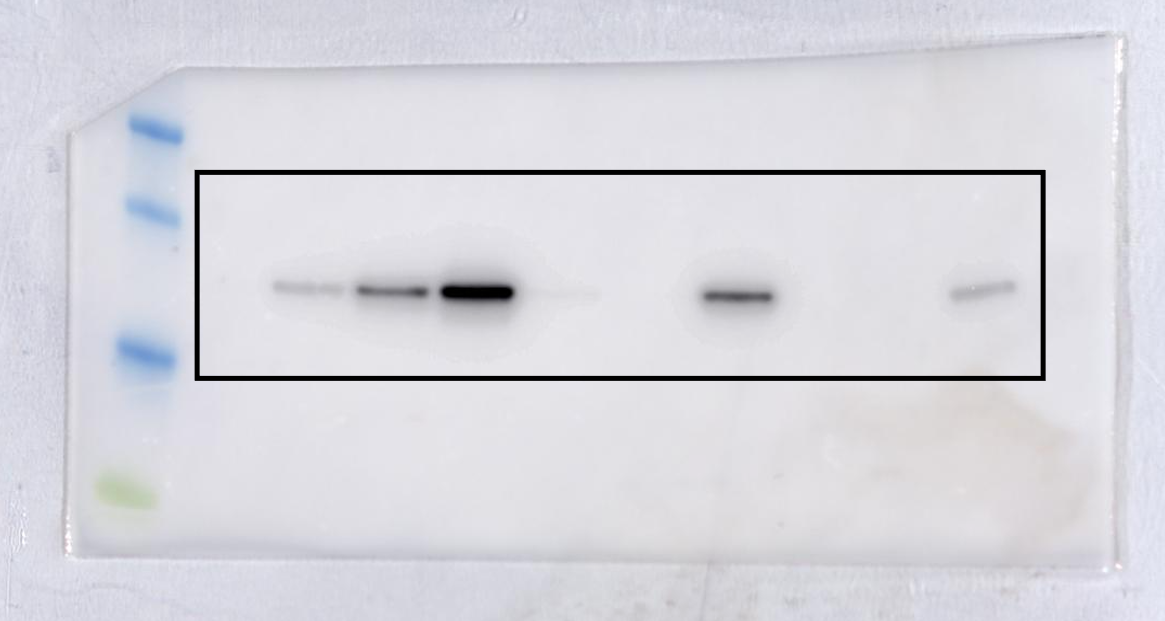

Supplement: Figure 3—source data 3. [file elife-81123-fig3-data3.tiff]

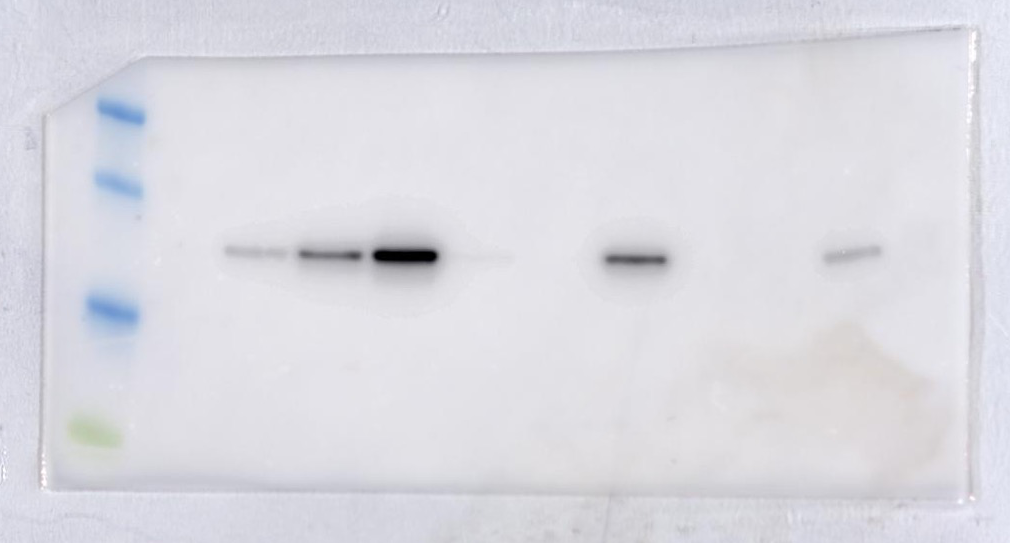

Supplement: Figure 3—source data 4. [file elife-81123-fig3-data4.tiff]

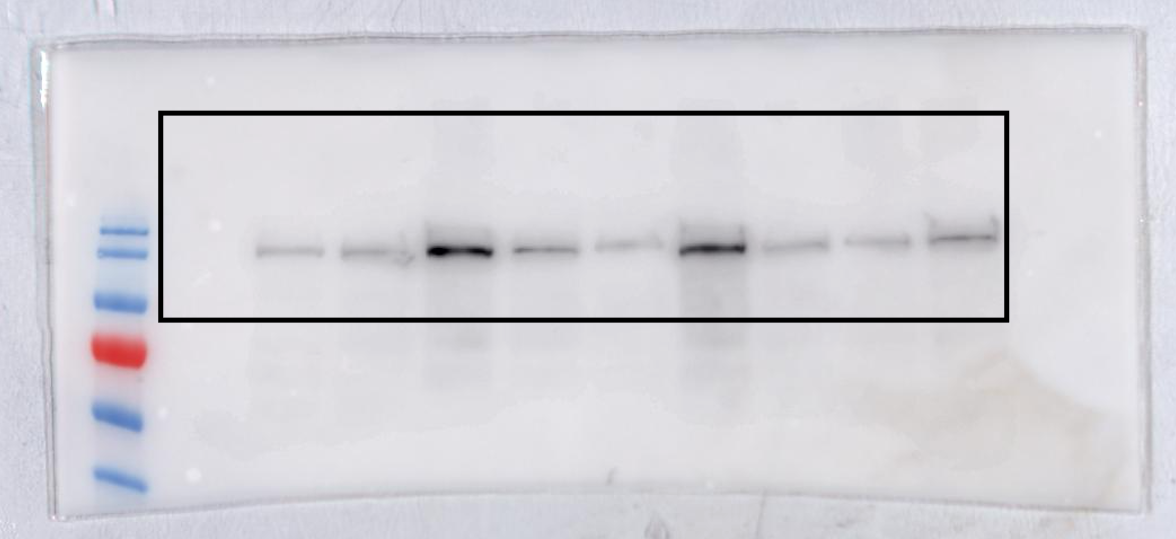

Supplement: Figure 3—source data 5. [file elife-81123-fig3-data5.tiff]

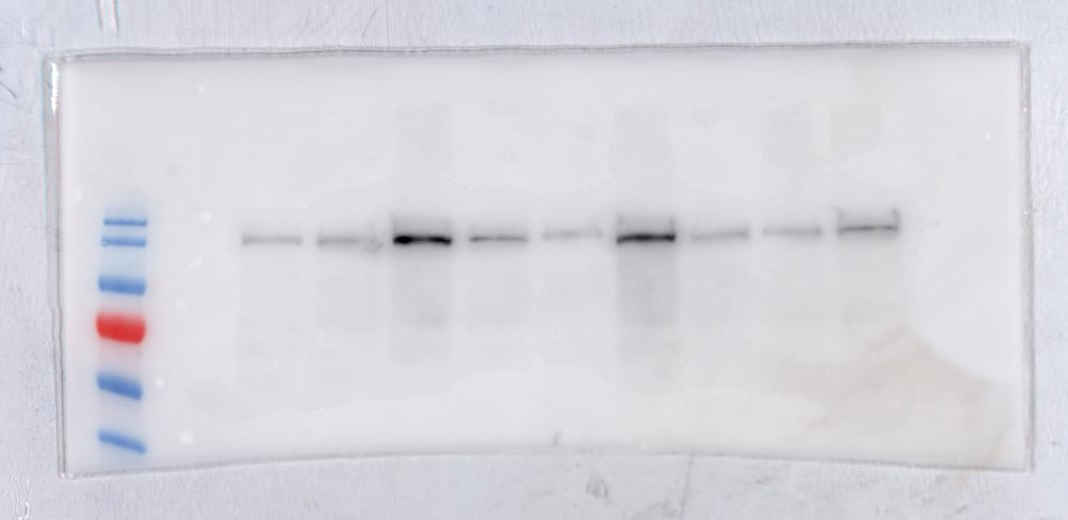

Supplement: Figure 3—source data 6. [file elife-81123-fig3-data6.tiff]

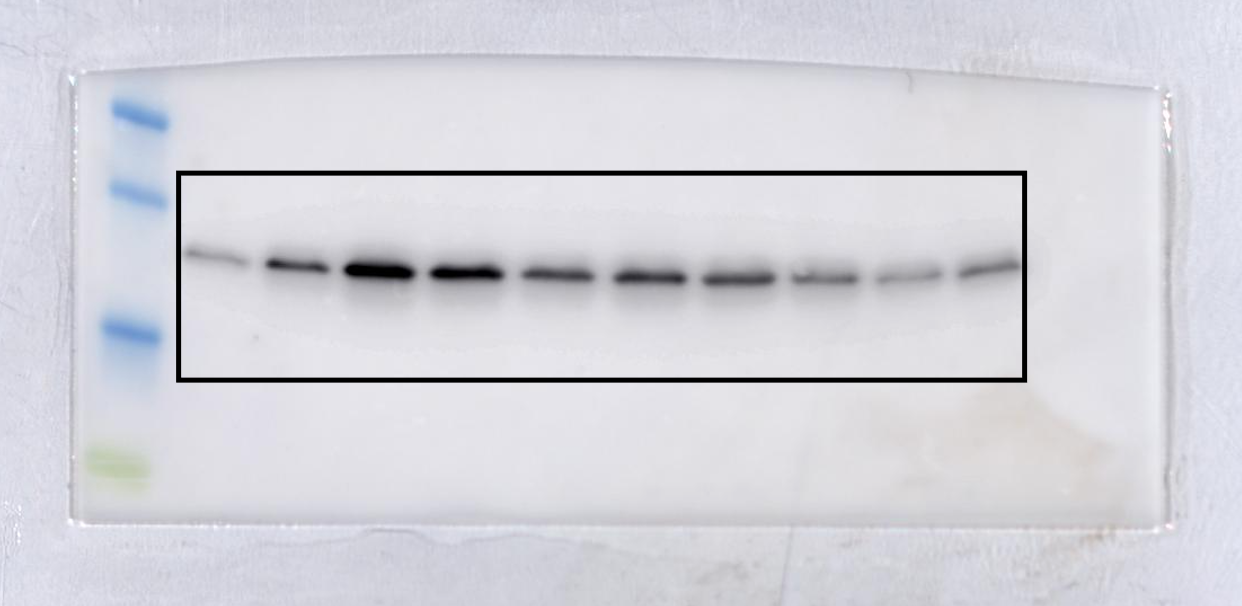

Supplement: Figure 3—source data 7. [file elife-81123-fig3-data7.tiff]

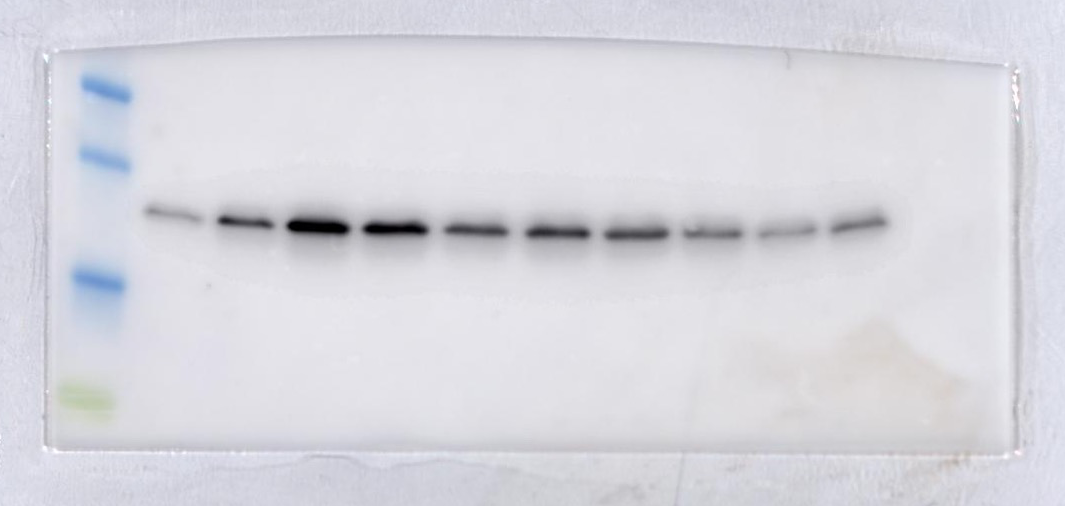

Supplement: Figure 3—source data 8. [file elife-81123-fig3-data8.tiff]

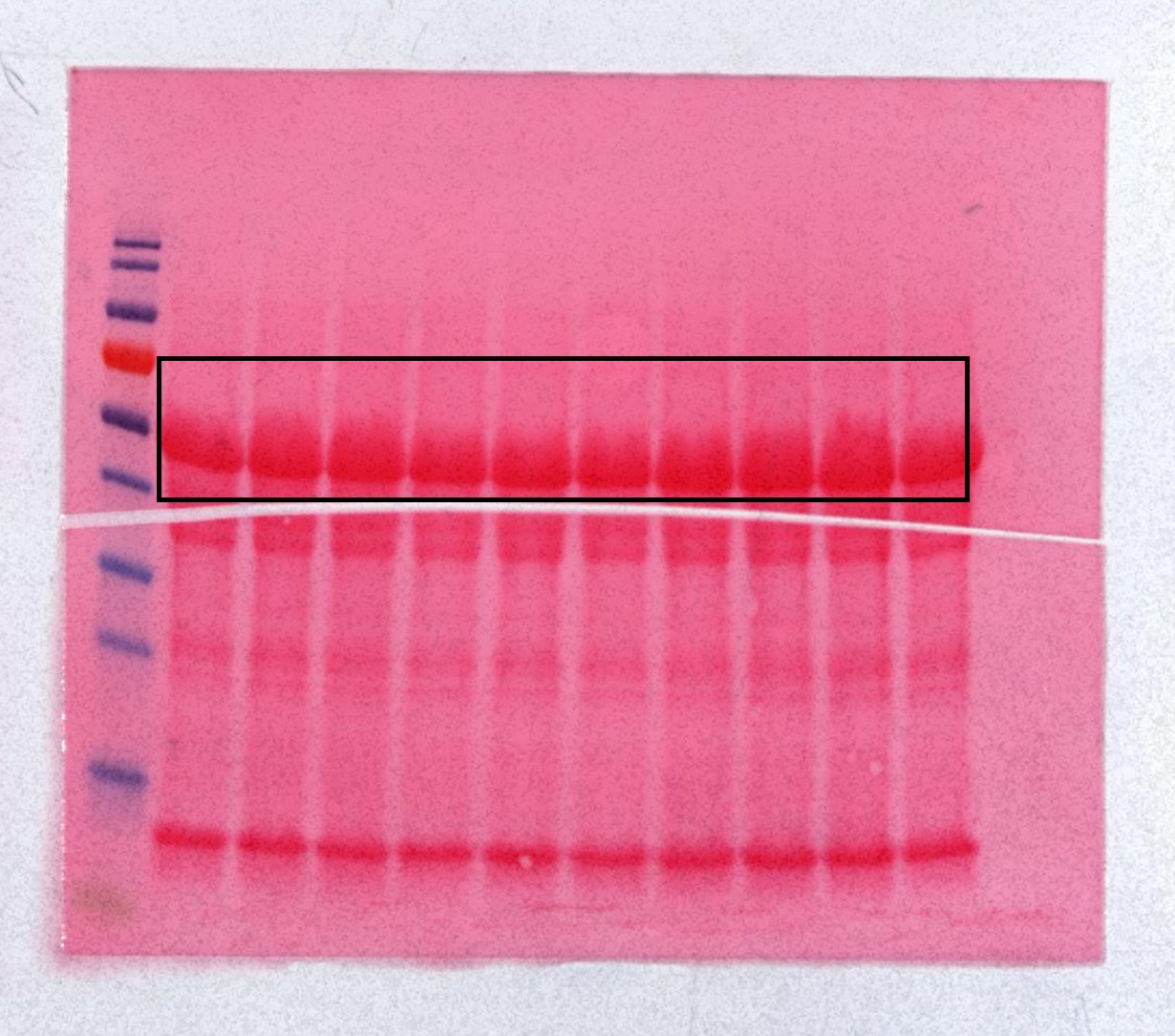

Supplement: Figure 3—source data 9. [file elife-81123-fig3-data9.tiff]

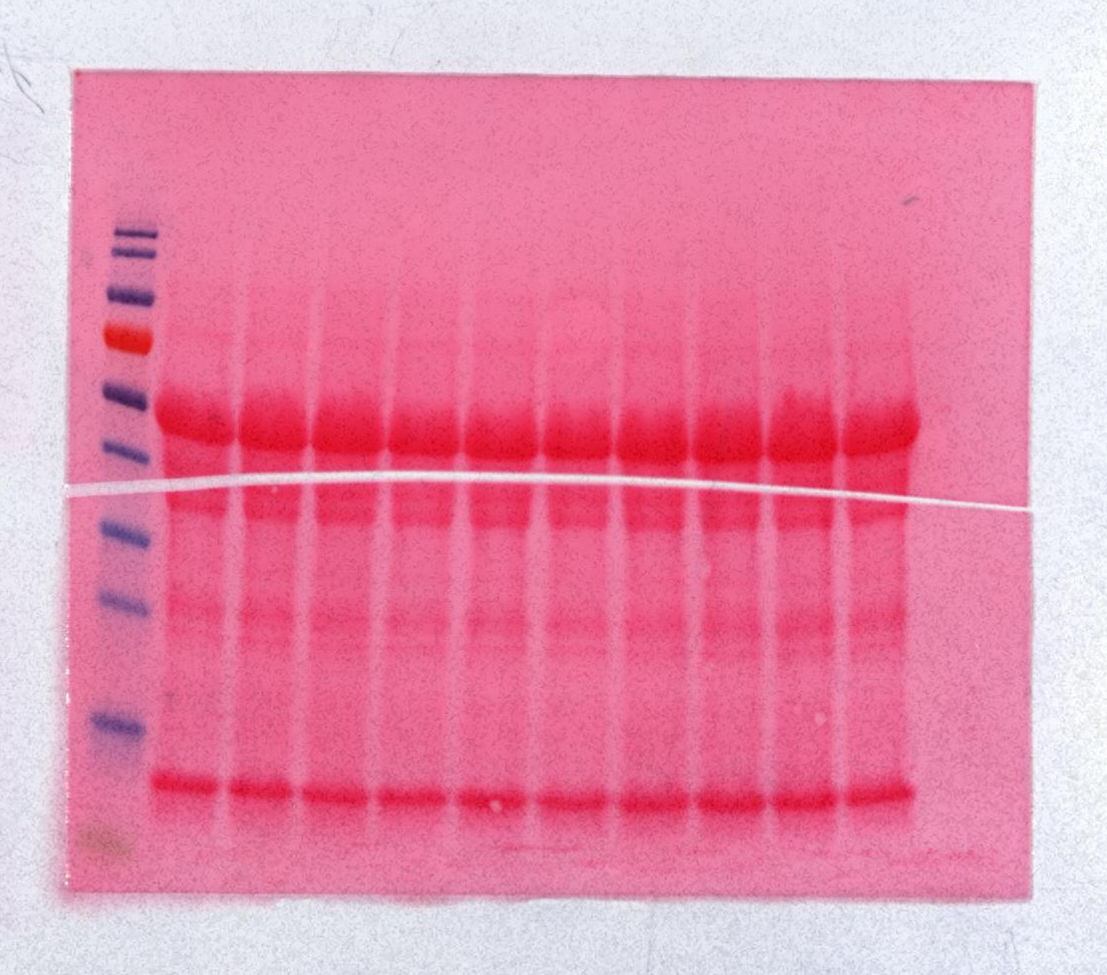

Supplement: Figure 3—source data 10. [file elife-81123-fig3-data10.tiff]
