# Supplementary material for: Effector target-guided engineering of an integrated domain expands the disease resistance profile of a rice NLR immune receptor
Source: eLife. 2023 May 18;12:e81123. doi: 10.7554/eLife.81123 (PMC10195085; doi:10.7554/eLife.81123)
Supplement: Supplementary file 1. — X-ray data collection and refinement statistics for Pikp-HMANK-KE/AVR-PikC (PDB entry 7A8W), Pikp-HMASNK-EKE/AVR-PikC (PDB entry 7QPX), and Pikp-HMASNK-EKE/AVR-PikF (PDB entry 7QZD). [file elife-81123-supp1.docx]

**Table S1** – X-ray data collection and refinement statistics for Pikp-HMA^NK-KE^/AVR-PikC (PDB entry 7A8W), Pikp-HMA^SNK-EKE^/AVR-PikC (PDB entry 7QPX), and Pikp-HMA^SNK-EKE^/AVR-PikF (PDB entry 7QZD).

|  | **Pikp^NK-KE^:AVR-PikC** | **Pikp^SNK-EKE^:AVR-PikC** | **Pikp^SNK-EKE^:AVR-PikF** |
| --- | --- | --- | --- |
| **Data collection statistics** |  |  |  |
| Wavelength (Å) | 0.9795 | 0.9795 | 0.9795 |
| Space group | *P* 2_1_ 2_1_ 2_1_ | *P* 2_1_ 2_1_ 2_1_ | *P* 2_1_ 2_1_ 2_1_ |
| Cell dimensions: |  |  |  |
| *a*, *b*, *c* (Å) | 66.78, 80.21, 105.68 | 66.35, 83.13, 107.04 | 67.38, 80.78, 103.80 |
| α, β, γ (°) | 90.00, 90.00, 90.00 | 90.00, 90.00, 90.00 | 90.00, 90.00, 90.00 |
| Resolution (Å)* | 46.17-2.15 (2.22-2.15) | 46.67-2.05 (2.11-2.05) | 103.80-2.20 (2.27-2.20) |
| *R*_merge_ (%)^#^ | 5.3 (99.6) | 8.1 (104.8) | 9.8 (75.1) |
| *I*/σ*I*^#^ | 23.9 (2.3) | 15.2 (2.0) | 11.5 (2.2) |
| Completeness (%)^#^ | 99.9 (99.8) | 99.8 (99.4) | 96.8 (98.6) |
| Unique reflections^#^ | 31604 (2694) | 37795 (2886) | 27072 (2013) |
| Redundancy^#^ | 13.2 (13.7) | 9.9 (10.1) | 6.9 (6.7) |
| CC^(1/2)^ (%)^#^ | 100.0 (92.5) | 99.9 (83.8) | 99.9 (84.8) |
| **Refinement/model statistics** | | | |
| Resolution (Å) | 44.16-2.15 (2.21-2.15) | 46.71-2.05 (2.10-2.05) | 63.83-2.20 (2.26-2.20) |
| *R*_work_/*R*_free_ (%)^^^ | 22.2/27.1 (36.1/34.1) | 20.4/22.7 (27.7/29.5) | 23.1/28.5 (36.3/37.2) |
| No. atoms (Protein) | 6959 | 7016 | 3526 |
| No. atoms (Water) | 78 | 203 | 109 |
| B-factors (Protein) | 64.0 | 46.7 | 44.6 |
| B-factors (Water) | 57.4 | 46.8 | 36.7 |
| R.m.s. deviations:^^^ |  |  |  |
| Bond lengths (Å) | 0.008 | 0.008 | 0.009 |
| Bond angles (º) | 1.479 | 1.519 | 1.597 |
| Ramachandran plot (%): ** |  |  |  |
| Favoured | 97.2 | 98.6 | 97.1 |
| Allowed | 2.8 | 1.4 | 2.7 |
| Outliers | 0.00 | 0.00 | 0.2 |
| MolProbity Score | 1.87 (89^th^ percentile) | 1.60 (95^th^ percentile) | 1.93 (89^th^ percentile) |

*The highest resolution shell is shown in parenthesis.

^#^As calculated by Aimless, ^^^As calculated by Refmac5, **As calculated by MolProbity
