# Supplementary material for: Effector target-guided engineering of an integrated domain expands the disease resistance profile of a rice NLR immune receptor
Source: eLife. 2023 May 18;12:e81123. doi: 10.7554/eLife.81123 (PMC10195085; doi:10.7554/eLife.81123)
Supplement: Supplementary file 2. — Summary of interface analysis by QtPISA for Pikp-HMANK-KE/AVR-PikC (PDB entry 7A8W), Pikp-HMASNK-EKE/AVR-PikC (PDB entry 7QPX), and Pikp-HMASNK-EKE/AVR-PikF (PDB entry 7QZD). Protein chains used for the analysis in each complex (as defined in the PDB entries) are: PikpNK-KE:AVR-PikC (E and F); PikpSNK-EKE:AVR-PikC (E and F); PikpSNK-EKE:AVR-PikF (F and G). [file elife-81123-supp2.docx]

**Table S2** – Summary of interface analysis by QtPISA for Pikp-HMA^NK-KE^/AVR-PikC (PDB entry 7A8W), Pikp-HMA^SNK-EKE^/AVR-PikC (PDB entry 7QPX), and Pikp-HMA^SNK-EKE^/AVR-PikF (PDB entry 7QZD). Protein chains used for the analysis in each complex (as defined in the PDB entries) are: Pikp^NK-KE^:AVR-PikC (E and F); Pikp^SNK-EKE^:AVR-PikC (E and F); Pikp^SNK-EKE^:AVR-PikF (F and G).

|  |  | **Pikp^NK-KE^:AVR-PikC** | **Pikp^SNK-EKE^:AVR-PikC** | **Pikp^SNK-EKE^:AVR-PikF** |
| --- | --- | --- | --- | --- |
| AVR-Pik | B.S.A. (Å) | 975.4 | 979.2 | 974.1 |
|  | % B.S.A. of total | 18.0 | 18.2 | 18.0 |
| HMA | B.S.A. (Å) | 1019.0 | 1047.0 | 1037.6 |
|  | % B.S.A. of total | 21.5 | 22.4 | 21.4 |
| Total interface area* (Å) | | 997.2 | 1013.1 | 1005.9 |
| Solvation energy (kcal/mol) | | -5.2 | -3.2 | -2.4 |
| Binding energy (kcal/mol) | | -13.6 | -13.2 | -13.6 |
| Hydrophobic p-value | | 0.4969 | 0.5560 | 0.6137 |
| Hydrogen bonds | | 12 | 15 | 16 |
| Salt bridges | | 8 | 9 | 11 |
| Disulphide bonds | | 0 | 0 | 0 |

*Total interface area is the total B.S.A. (Buried Surface Area) of each component divided by two.
